# Enantiospecific Three‐Component Alkylation of Furan and Indole

**DOI:** 10.1002/chem.201800527

**Published:** 2018-02-28

**Authors:** Mattia Silvi, Raffael Schrof, Adam Noble, Varinder K. Aggarwal

**Affiliations:** ^1^ School of Chemistry University of Bristol Cantock's Close BS8 1TS Bristol United Kingdom

**Keywords:** alkylation, boron, heterocycles, metallate rearrangement, photoredox catalysis

## Abstract

Furan‐ and indole‐derived boronate complexes react with alkyl iodides under radical (photoredox) or polar (S_N_2) conditions to generate three‐component alkylation products with high efficiency and complete stereospecificity. The methodology allows the incorporation of versatile functional groups such as nitriles, ketones, esters, sulfones, and amides, providing rapid access to complex chiral heteroaromatic molecules in enantioenriched form. Interestingly, while indolyl boronate complexes react directly with alkyl halides in a polar pathway, furyl boronates require photoredox catalysis. Careful mechanistic analysis revealed that the boronate complex not only serves as a substrate in the reaction but also acts as a reductive quencher for the excited state of the photocatalyst.

Heteroaromatic compounds are ubiquitous in natural products and bioactive compounds and play a central role in organic chemistry. In particular, the furan ring is a versatile moiety for organic synthesis, readily undergoing oxidations, acid‐catalyzed rearrangements and cycloaddition reactions,^1^ while the indole ring is one of the most common motifs in alkaloids and drug candidates.^2^ Our group recently introduced an enantiospecific sp^3^–sp^2^ coupling between aromatic rings and chiral boronic esters **1** to access a variety of enantioenriched monofunctionalized aromatic molecules (e.g. **3**, Scheme 1 a).^3^ Furthermore, inspired by the intriguing reactivity of unsaturated boronate complexes,^4^ we showed that nucleophilic furyl‐derived boronate complexes **2** react with electrophiles in an enantiospecific three‐component coupling reaction (leading to **4**, Scheme 1 a).^5^ Key to the success of this process was the use of highly activated electrophiles, such as the Umemoto trifleoromethylating agent^6^ or carbocationic species. Interestingly, the trifluoromethylation reaction was found to proceed through a self‐initiated radical chain mechanism. Independently, Studer and our group have shown that electrophilic radicals add to vinyl boronate complexes **5** (Scheme 1 b).^7^ The resulting α‐boronate radicals **7** undergo facile single electron oxidation to trigger 1,2‐migration and form boronic ester adducts **8**.

**Scheme 1:**
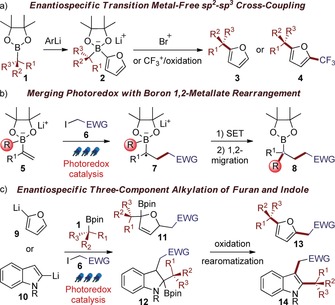
a) Enantiospecific cross‐coupling and three‐component trifluoromethylation of furans. b) Photoredox‐mediated three‐component alkylation of vinyl boronic esters. c) Planned strategy: photoredox‐mediated enantiospecific three‐component coupling of furan and indole. SET: single electron transfer, EWG: electron‐withdrawing group, pin: pinacolato.

We envisioned extending this merger of photoredox catalysis^8^ with boron 1,2‐metallate rearrangements to aromatic boronate systems (Scheme 1 c).^9^, ^10^ Photoredox radical‐mediated reaction of alkyl halides **6** 11 with boronate complexes derived from chiral boronic esters **1** and aryllithiums **9** and **10** should lead to dearomatized intermediates **11** and **12**. As 1,2‐migration is well‐known to be a stereospecific process^12^ the stereochemistry within boronic ester **1** should be conserved in the process. In situ oxidation/rearomatization of **11** and **12** would then provide chiral aromatic compounds **13** and **14** in enantioenriched form. Such a transformation would constitute a novel enantiospecific three‐component alkylation protocol, providing valuable routes to complex chiral heteroaromatic structures. Herein, we describe the successful development of this new methodology, which takes advantage of the versatility and mild conditions of photoredox catalysis to introduce a suite of synthetically valuable functional groups.

Attracted by the versatility of nitriles in organic synthesis,^13^ we commenced our studies by investigating the reaction of furan with iodoacetonitrile (**6 a**) (Table 1).^14^ Boronate complex **2 a** was formed by addition of cyclohexyl boronic acid pinacol ester (**1 a**) to 2‐furyllithium, generated by lithiation of furan with *n‐*butyllithium in THF. Solutions of iodoacetonitrile (**6 a**) and a photocatalyst were then added and the mixture irradiated with blue LEDs for 1 h. Gratifyingly, in a preliminary experiment carried out using acetonitrile/THF as solvent (entry 1), we found that the reaction proceeded smoothly to give the desired dearomatized intermediate **11 a** in 50 % yield using Ru(bpy)_3_^2+^ as a photocatalyst. A solvent screen led to the identification of a mixture of DMF/THF (2:1) as optimal, giving **11 a** in 73 % yield (entry 4). The use of the more reducing photocatalyst Ir(ppy)_3_ gave similar results (entry 5). In contrast to the photochemical three‐component alkylation of vinyl boronate complexes,7a we observed that the use of a photocatalyst was crucial for obtaining high yields in this reaction (compare entries 4 and 6). A control experiment carried out in the dark did not give any product (entry 7), showing the photochemical nature of this transformation. Intermediate **11 a** could be oxidized by addition of iodine and potassium acetate^15^ to the reaction vessel to give the desired three‐component alkylation product **13 a** in 71 % isolated yield (entry 4).

**Table 1:** Reaction optimization.

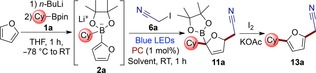			
Entry^[a]^	Solvent^[b]^	Photocatalyst [PC]	Yield of **11** **a** [%]^[c]^
1	CH_3_CN/THF	Ru(bpy)_3_Cl_2_⋅6 H_2_O	50
2	DMSO/THF	Ru(bpy)_3_Cl_2_⋅6 H_2_O	66
3	DMI/THF	Ru(bpy)_3_Cl_2_⋅6 H_2_O	70
4	DMF/THF	Ru(bpy)_3_Cl_2_⋅6 H_2_O	73 (71)
5	DMF/THF	Ir(ppy)_3_	71
6^[d]^	DMF/THF	–	26
7^[e]^	DMF/THF	Ru(bpy)_3_Cl_2_⋅6 H_2_O	0

[a] All the reactions were carried out using 1.2 equiv of furan, 1.15 equiv of *n‐*butyllithium, 1.0 equiv of **1 a** and 1.5 equiv of iodoacetonitrile **6 a** on a 0.2 mmol scale. [b] Mixture of solvents are intended solvent/THF 2:1. DMI: 1,3‐dimethyl‐2‐imidazolidinone. [c] Yield measured through NMR analysis of the crude mixture using dibromomethane as an internal standard. Intermediate **11 a** was obtained as a 1:1 mixture of diastereoisomers. Number in parenthesis is the isolated yield of compound **13 a** after oxidation and chromatographic purification. [d] Photochemical step time: 2 hours. [e] Reaction carried out in the dark.

Having identified the optimum conditions, we explored the scope of our photochemical transformation (Scheme 2). A wide range of synthetically versatile functional groups could be efficiently introduced by varying the alkyl iodide radical precursor. Iodoacetophenones proved to be good substrates, leading to the corresponding ketones in good yields (**13 b** and **13 c**). Ethyl iodoacetate and iodoacetamide provided ester **13 d** and unprotected amide **13 e**. Iodomethyl phenylsulfone led to sulfone **13 g** in 64 % yield.^16^, ^17^ In addition, perfluoroalkyl chains could be connected to the furan scaffold (**13 f**) starting from readily available perfluoroalkyl iodides. The employment of tertiary and primary boronic esters led to products **13 h** and **13 i** in good yields, demonstrating that the process tolerates a wide spectrum of steric demand. Furthermore, starting from enantioenriched chiral boronic esters, compounds **13 j** and **13 k** were obtained with complete stereospecificity.

**Scheme 2:**
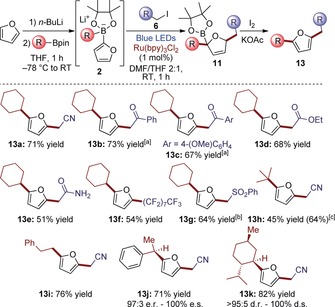
Scope of the enantiospecific three‐component alkylation of furan. All the yields refer to isolated product after chromatographic purification. [a] Intermediate oxidation conditions: NaClO (aq.), DMF, −20 °C. [b] 4 equiv of alkyl iodide were used. [c] Volatile product, number in parenthesis is the NMR yield using CH_2_Br_2_ as internal standard.

Attracted by the importance of the indole ring in synthetic and medicinal chemistry, we sought to extend our enantiospecific three‐component alkylation reaction to *N*‐methyl indole (Scheme 3). Pleasingly, the reaction of boronate **15** with iodoacetonitrile (**6 a**) gave the desired product **14 a** in 70 % yield. However, in control experiments we found that neither light nor the photocatalyst were required for the transformation. Furthermore, addition of the radical inhibitor 1,1‐diphenylethylene7a had no effect on the reaction outcome (see Supporting Information–3.5 for details), ruling out a possible electron‐transfer initiated radical chain process^5^ and supporting a polar S_N_2‐like pathway. The nucleophilic reactivity of indole‐derived borates generated from difficult‐to‐handle trialkyl boranes has been described,^18^ however the difficulties in accessing enantioenriched chiral boranes has prevented the use of this methodology for the synthesis of chiral compounds in enantioenriched form. Surprisingly, reactions of boronates derived from stable boronic esters are rare with only a single report very recently disclosed by Ready et al. (with π‐allyl palladium complexes).^19^

**Scheme 3:**
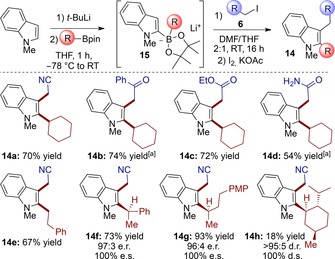
Scope of the enantiospecific three‐component alkylation of indole. All the yields refer to the isolated product after chromatographic purification. [a] Intermediate oxidation conditions: H_2_O_2_(aq.)/NaOH(aq.), 0 °C, DMF/THF 2:1.

Through our simple protocol, indole‐derived boronates **15** could be alkylated to introduce a diverse range of functional groups, including nitriles, ketones, esters and unprotected amides, providing functionalized indoles **14 a**–**d** in good yields. Various other alkyl boronic esters were also applied to the coupling reaction, including primary (**14 e**) and enantioenriched chiral secondary (**14 f** and **14 g**) examples, all proceeding in high yields and with complete stereospecificity. However, using the bulky menthyl boronic ester provided the desired product **14 h** in only 18 % yield (albeit with excellent stereospecificity) showing that steric hindrance has an impact in this reaction. Finally, a simple control experiment showed that *N*‐methylindole does not undergo Friedel–Crafts alkylation with iodoacetonitrile **6 a** under our reaction conditions (see Supporting Information–3.5 for details), thus highlighting the importance of the boronate σ‐donation to the indolyl π‐system for the nucleophilicity of **15**. Indeed, Mayr has shown that a BF_3_K moiety (which is not as electron‐donating as RBpinLi) at the 2 position increases the nucleophilicity of *N*‐Boc indole by >10^5^.^20^

To glean insights into the mechanism of the furan three‐component coupling reaction, selected spectroscopic and electrochemical studies were carried out. Quantum yield measurements gave a value of *Φ*=27.8 (see Supporting Information–3.4 for details), suggesting a radical chain pathway to be operative.^21^ Fluorescence quenching analysis revealed that boronate complex **2 a** was an effective quencher of the excited state photocatalyst, whereas iodoacetonitrile (**6 a**) was ineffective (see Supporting Information–3.2 for details). Based on these results, we propose the mechanism depicted in Scheme 4. The highly reducing Ru^I^ is generated by single electron transfer (SET) from a sacrificial amount of boronate complex **2 a** (*E*_p/2_=+0.26 V vs. SCE, Figure S5) to the excited Ru^II^ catalyst (*E* ^0^[Ru^II*/I^]*=*+0.77 V vs. SCE).^22^, ^23^ Since one electron oxidation of analogous boronate complexes has been shown to lead to the generation of alkyl radicals through C−B bond fragmentation,^24^ this reductive quenching phenomenon is expected to lead to by‐product **16**, which was indeed observed in the crude reaction mixtures. Once formed, the electron‐rich Ru^I^ species (*E* ^0^[Ru^I/II^]=−1.33 V vs. SCE) undergoes single electron transfer with iodoacetonitrile (**6 a**, *E*_p/2_=−1.24 V vs. SCE, Figure S6) leading to the formation of the reactive electrophilic radical **17** and the regeneration of the Ru^II^ catalyst. Radical **17** then adds to the furyl system of **2 a** generating radical anion **18**. The electron‐rich radical anion is expected to undergo SET with another molecule of iodoacetonitrile (**6 a**),^7^, ^25^ forming radical **17** and a zwitterionic species (not shown), which undergoes 1,2‐migration to release intermediate **11 a**.^26^, ^27^ In this process, the excited Ru^II^* photocatalyst operates a smart initiation,^28^ with the Ru^I^ species being regenerated by reductive quenching of Ru^II^* either by intermediate **18** or by another molecule of boronate **2 a**. Considering the high bimolecular quenching rate constant found for boronate **2 a** (*k*_q_=1.98×10^9^ m ^−1^ s^−1^, close to the diffusion limit, see Supporting Information–3.2 for details) and the expected low concentration of reactive intermediate **18** in solution, we believe that the second option is more likely. In this scenario a sacrificial amount of boronate **2 a** is required, rather like a “tax” that has to be paid, to sustain the radical chain and balance undesired termination events.

**Scheme 4:**
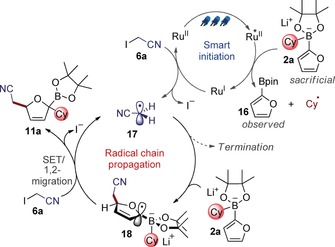
Proposed reaction mechanism for the three‐component alkylation of furan.

In conclusion, we have developed a novel stereospecific three‐component alkylation reaction of furans and indoles with boronic esters and electron‐deficient alkyl iodides. Mechanistically, the more electron‐rich indole boronates are sufficiently nucleophilic to react directly with alkyl iodides through a polar pathway. Conversely, alkylation of the less reactive furyl boronates proceeded through a radical pathway induced by photoredox catalysis. Careful mechanistic analysis showed that the furyl boronate complex **2** plays a dual role, acting both as sacrificial reductive quencher for Ru^II^* (giving the reductant Ru^I^) and as reactant for the three‐component alkylation reaction.

## Conflict of interest

The authors declare no conflict of interest.

## Supporting information

As a service to our authors and readers, this journal provides supporting information supplied by the authors. Such materials are peer reviewed and may be re‐organized for online delivery, but are not copy‐edited or typeset. Technical support issues arising from supporting information (other than missing files) should be addressed to the authors.

Supplementary
