# Supplementary material for: Enantiospecific Three‐Component Alkylation of Furan and Indole
Source: Chemistry. 2018 Feb 28;24(17):4279–82. doi: 10.1002/chem.201800527 (PMC5900874; doi:10.1002/chem.201800527)

CHEMISTRY
A European Journal

Supporting Information

Enantiospecific Three-Component Alkylation of Furan and Indole

Mattia Silvi, Raffael Schrof, Adam Noble, and Varinder K. Aggarwal*[a]

chem_201800527_sm_miscellaneous_information.pdf

Contents

1. General Experimental 2

1.1 Solvents, Reagents and Starting Materials 2

1.2 Chromatography and Instrumental Analysis 2

1.3 Naming of Compounds 3

2. Procedures & Product Characterization 4

2.1 General Procedure for Furan Three Component Couplings 4

2.2 General Procedure for Indole Three Component Couplings 5

2.3 Reaction Products and Characterization 6

3. Mechanistic Studies 19

3.1 Synthesis and Characterization of Boronate Complex 2a 19

3.2 Fluorescence Quenching Experiments 19

3.3 Electrochemical Experiments 22

3.4 Quantum Yield Measurements 24

3.5 Control Experiments for Indole Three Component Couplings 27

4. References 29

5. HPLC Chromatograms 30

6. NMR Spectra 33

1. General Experimental

1.1 Solvents, Reagents and Starting Materials

All air and water-sensitive reactions were carried out in oven-dried glassware under a nitrogen atmosphere using standard Schlenk manifold technique. Bulk solutions were evaporated under reduced pressure using a Büchi rotary evaporator. All solvents were commercially supplied or provided by the communal stills of the School of Chemistry, University of Bristol. *n*-BuLi was purchased from Acros. *tert*-BuLi was purchased from Sigma-Aldrich. The molarity of organolithium solutions was determined by titration using *N*-benzyl benzamide or diphenylacetic acid as an indicator. Iodoacetonitrile, iodoacetamide, ethyl iodoacetate were purchased from Sigma Aldrich and used as received. Iodoacetophenone[1] and 2-iodo-1-(4-methoxyphenyl)ethan-1-one[2] are known and were synthesized in one step through Finkelstein reaction starting from the corresponding bromides following procedures reported in the literature. Iodophenyl sulfone was synthesized following a literature procedure.[3] Boronic esters **1c**,[4] **1d**,[5] **1e**,[6] **1f**[7] are known and were synthesized according to literature procedures. All other reagents were purchased from commercial sources and used as sold, unless noted.

1.2 Chromatography and Instrumental Analysis

Flash column chromatography (FCC) was carried out using Sigma-Aldrich silica gel LC60A-40 (63 µm). All reactions were followed by thin-layer chromatography (TLC) when practical, using Merck Kieselgel 60 F_{254} fluorescent treated silica which was visualised under UV light or by staining with aqueous basic potassium permanganate.

^{1}H and ^{13}C NMR spectra were recorded using Jeol ECP (Eclipse) 300 MHz, Jeol ECS 400 MHz, Varian VNMR 400 MHz, Bruker 400 MHz, Varian VNMR 500 MHz and Bruker Cryo 500 MHz spectrometers. Chemical shifts (δ) are given in parts per million (ppm), and coupling constants (*J*) are given in Hertz (Hz). The ^{1}H NMR spectra are reported as follows: ppm (multiplicity, coupling constants, number of protons).

High resolution mass spectra (**HRMS**) were recorded on a Bruker Daltonics MicrOTOF II by Electrospray Ionisation (ESI). **IR spectra** were recorded on a Perkin Elmer Spectrum One FT-IR as a thin film. Only selected absorption maxima (ν_{max}) are reported in wavenumbers (cm^{-1}). **Melting points** were recorded in degrees Celsius (°C), using a Kofler hot-stage

microscope apparatus and are reported uncorrected. **Fluorescence spectra** were recorded using a Varian Cary Eclipse fluorimeter, equipped with a Xenon pulse lamp, pulsed frequency 80 Hz, pulse width at half peak height ~ 2 μs, peak power equivalent to 75 kW. **Electrochemical experiments** were carried out using a Basi Epsilon EC potentiostat offering compliance voltage up to ± 100 V (available at the counter electrode), ± 10 V scan range and ± 2 A current range. A glassy carbon working electrode, a platinum wire counter electrode, and a 3 M NaCl Ag/AgCl reference electrode were used to perform all electrochemical analysis. For **quantum yield** experiments commercially available potassium ferrioxalate trihydrate (Alfa Aesar) was used for actinometry, and all the absorption experiments were carried out using a Perkin Elmer Lambda 25 UV/Vis Spectrophotometer.

1.3 Naming of Compounds

Compound names are those generated by ChemBioDraw 13.0 software (PerkinElmer), following the IUPAC nomenclature.

2. Procedures & Product Characterization

2.1 General Procedure for Furan Three Component Couplings

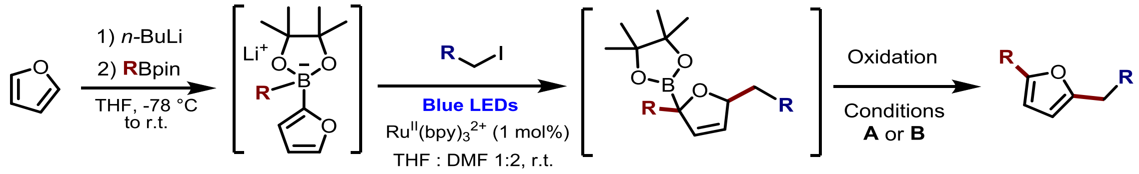

To a -78 °C stirred solution of furan (17.5 µL, 0.240 mmol, 1.20 equiv.) in THF (0.35 mL) under N_2 atmosphere was added dropwise a 1.6 M solution of *n*-butyllithium in hexanes (145 µL, 0.230 mmol, 1.15 equiv.). The solution was left to warm to r.t. under vigorous stirring over 30 minutes, after which it was cooled to -78 °C. The corresponding boronic ester was placed in a separate oven-dried test tube and dissolved in dry THF (0.3 mL) under a nitrogen atmosphere. The boronic ester solution was added dropwise to the cold reaction mixture under vigorous stirring. To ensure quantitative transfer of the material the test tube was washed twice with 0.2 mL of THF followed by dropwise transfer to the reaction mixture. The resulting solution was then left to warm to r.t. and stirred for 30 minutes. The reaction vessel was placed into a crystallizing basin equipped with 1.5 m length blue LED strip. To the boronate solution, a degassed solution (15 minutes of N_2 sparging)[i] of the alkyl iodide (0.30 mmol, 1.5 equiv.) and Ru(bpy)$_3$·6H_2O (1.5 mg, 0.0020 mmol, 1.0 mol%) in DMF (2.0 mL) was added under irradiation, and the mixture was stirred vigorously for 1 h under constant irradiation. Oxidation procedures A or B were then carried out.

Oxidation Procedure **A**

Iodine (265 mg, 1.00 mmol, 5.00 equiv.) and potassium acetate (195 mg, 2.00 mmol, 10.0 equiv.) were added to the reaction mixture under vigorous stirring conditions. The reaction mixture was stirred at r.t. until full conversion was achieved (as judged from TLC analysis), typically 10 – 60 minutes. The reaction mixture was then diluted with EtOAc (150 mL) and washed with a 20% solution of sodium thiosulfate (50 ml), twice with water (100 mL + 4 mL brine to induce phase separation), and finally with brine (100 mL). The resulting organic phase

[i]In case of volatile alkyl iodides (ethyl iodoacetate, iodoacetonitrile, perfluoroalkyl iodides), N_2 sparging was performed on the photocatalyst solution prior to the addition of the alkyl halide under a nitrogen atmosphere.

was dried ($MgSO_4$), filtered and concentrated *in vacuo*. The crude product was purified by FCC.

Oxidation Procedure **B** – only for aryl ketones

The reaction mixture was diluted with EtOAc (150 mL) and washed with water (100 mL + 4 mL brine to induce phase separation) and brine (100 mL). The resulting organic phase was dried ($MgSO_4$), filtered and concentrated *in vacuo*. The reaction crude product was transferred to a 50 mL flask, diluted with DMF (2.6 mL) and cooled down to -20 °C. To this solution, 550 μL of a sodium hypochlorite solution (>8% available chlorine) was added dropwise and the resulting solution was stirred until full conversion was achieved (as judged from TLC analysis), typically 10 – 30 minutes. The reaction mixture was removed from the cold bath, and 40 mL of a saturated sodium thiosulfate solution (at r.t.) was quickly poured into the reaction mixture. The mixture was then diluted with a 20% solution of sodium thiosulfate (50 ml) and extracted twice with EtOAc (2 x 80 mL). The organic phases were combined and washed twice with water (100 mL + 4 mL brine to induce phase separation), and finally with brine (100 mL). The resulting organic phase was dried ($MgSO_4$), filtered and concentrated *in vacuo*. The crude product was purified by FCC.

2.2 General Procedure for Indole Three Component Couplings

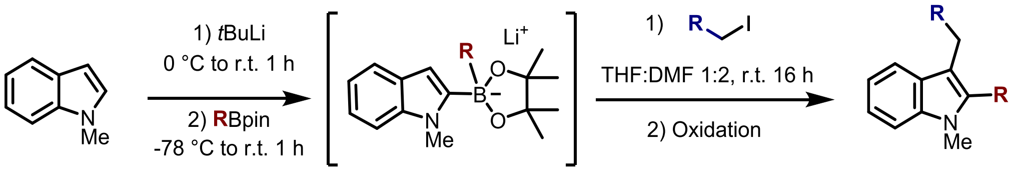

To a 0 °C stirred solution of *N*-methylindole (30 μL, 0.24 mmol, 1.2 equiv.) in THF (0.35 mL) under N_2 atmosphere was added dropwise a 1.7 M solution of *tert*-butyllithium in pentane (158 μL, 0.270 mmol, 1.35 equiv.). The solution was left to warm to r.t. and stirred for 60 minutes, resulting in the formation of a white precipitate. The reaction mixture was then cooled to -78 °C. The corresponding boronic ester was placed in a separate oven-dried test tube and dissolved in dry THF (0.3 mL) under a nitrogen atmosphere. The boronic ester solution was added dropwise to the cold reaction mixture under vigorous stirring. To ensure quantitative transfer of the material the test tube was washed twice with 0.2 mL of THF followed by dropwise transfer to the reaction mixture. The reaction was then left to warm at r.t. and stirred for 30 minutes, resulting in a clear solution. Afterwards, a solution of the alkyl iodide (0.30 mmol,

1.5 equiv.) in DMF (2.0 mL) was added quickly by syringe, and the mixture was stirred vigorously for 16 h. Oxidation procedures **A** (see previous section, reaction time was normally 60 minutes) or **C** were then carried out.

Oxidation Procedure **C**

The reaction mixture was cooled down to 0 °C. 300 μL of an aq. 2M NaOH solution and 200 μL of an aq. solution of H_2O_2 (30% W/V) were simultaneously added dropwise to the reaction mixture under vigorous stirring and left at the same temperature for 1 hour. The reaction mixture was then diluted with EtOAc (150 mL) and washed with a 20% solution of sodium thiosulfate (50 ml), twice with water (100 mL + 4 mL brine to induce phase separation), and finally with brine (100 mL). The resulting organic phase was dried ($MgSO_4$), filtered and concentrated *in vacuo*. The crude product was purified by FCC.

Reaction set-up for irradiation of mixture with blue LEDs:

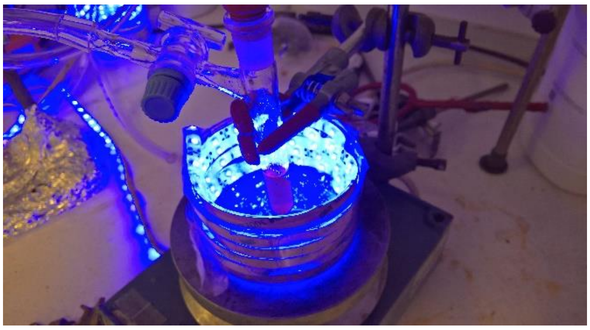

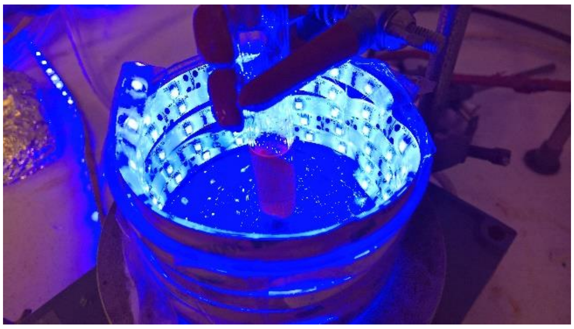

LED strip: Fluxia DIY-B60 LED kit. 60 LED per meter, 5 cm cut intervals, 5.76 W per meter, 130° – 150° beam angle.

2.3 Reaction Products and Characterization

2-(5-Cyclohexylfuran-2-yl)acetonitrile (13a)

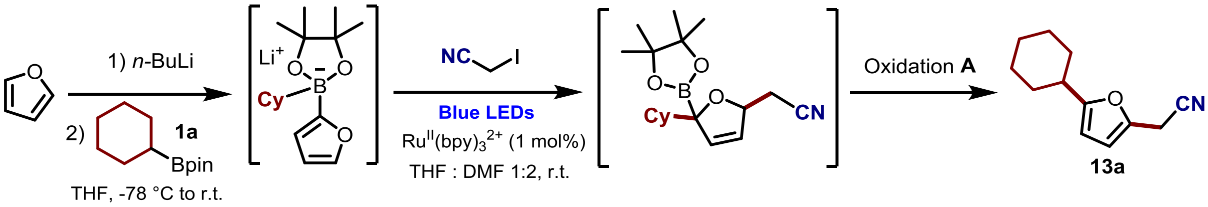

According to the General Procedure, using cyclohexyboronic acid pinacol ester (45 μL, 0.20 mmol, 1.0 equiv.) and iodoacetonitrile (50 mg, 22 μL, 0.30 mmol, 1.5 equiv.), following

oxidation procedure **A**. The crude product was purified by FCC (pentane/Et_2O = 100/0 to 96/4) to afford the title compound **13a** as a colourless oil in 71% yield (27 mg).

R$_f$ (85/15 *n*-hexane/EtOAc): 0.5; **IR** (film) ν_{max}/cm^{-1}: 2927, 2854, 2256, 1559, 1449, 1172, 1016, 973, 785; **^{1}H NMR** ($CDCl_3$, 500 MHz) δ (ppm): 6.19 (dt, J = 3.1, 1.0 Hz, 1H), 5.90 (dd, J = 3.1, 1.0 Hz, 1H), 3.71 (d, J = 1.0 Hz, 2H), 2.64 – 2.51 (m, 1H), 2.05 – 1.94 (m, 2H), 1.85 – 1.74 (m, 2H), 1.74 – 1.64 (m, 1H), 1.41 – 1.29 (m, 4H), 1.29 – 1.17 (m, 1H); **^{13}C NMR** ($CDCl_3$, 125 MHz) δ (ppm): 162.0, 140.6, 116.0, 108.9, 104.0, 37.3, 31.6, 26.2, 26.0, 17.7; **HRMS** (ESI) mass calculated for $[M+Na]^+$ ($C_{12}H_{15}NNaO^+$) requires *m/z* 212.1046, found *m/z* 212.1052.

2-(5-Cyclohexylfuran-2-yl)-1-phenylethan-1-one (13b)

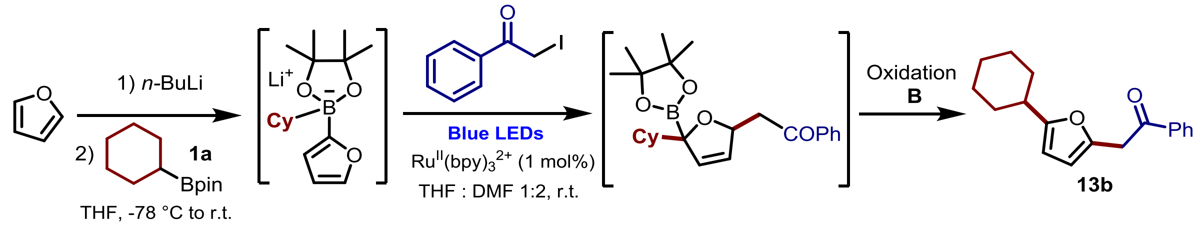

According to the General Procedure, using cyclohexyboronic acid pinacol ester (45 µL, 0.20 mmol, 1.0 equiv.) and iodoacetophenone (74 mg, 0.30 mmol, 1.5 equiv.), following oxidation procedure **B**. The crude product was purified by FCC (*n*-hexane/EtOAc = 100/0 to 96/4) to afford the title compound **13b** as a white solid in 73% yield (39 mg).

MP: 27-28 °C (*n*-hexane/EtOAc); **R$_f$** (85/15 *n*-hexane/EtOAc): 0.5; **IR** (film) ν_{max}/cm^{-1}: 2926, 2853, 1686, 1448, 1220, 1015, 781, 688; **^{1}H NMR** ($CDCl_3$, 400 MHz) δ (ppm): 8.05 – 7.96 (m, 2H), 7.61 – 7.52 (m, 1H), 7.52 – 7.42 (m, 2H), 6.10 (d, J = 3.0 Hz, 1H), 5.88 (dd, J = 3.0, 1.0 Hz, 1H), 4.26 (s, 2H), 2.66 – 2.46 (m, 1H), 2.07 – 1.90 (m, 2H), 1.84 – 1.63 (m, 3H), 1.41 – 1.16 (m, 5H); **^{13}C NMR** ($CDCl_3$, 100 MHz) δ (ppm): 195.4, 160.7, 145.9, 136.5, 133.3, 128.8, 128.7, 108.6, 103.8, 38.8, 37.3, 31.6, 26.2, 26.0; **HRMS** (ESI) mass calculated for $[M+Na]^+$ ($C_{20}H_{31}BNaO_3^+$) requires *m/z* 291.1355, found *m/z* 291.1370.

2-(5-Cyclohexylfuran-2-yl)-1-(4-methoxyphenyl)ethan-1-one (13c)

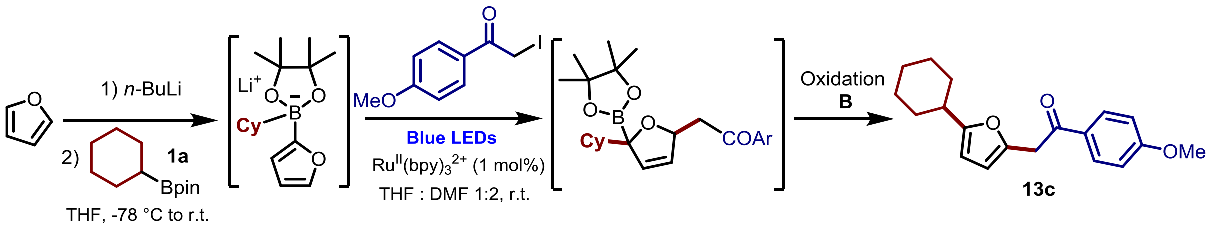

According to the General Procedure, using cyclohexyboronic acid pinacol ester (45 µL, 0.20 mmol, 1.0 equiv.) and 2-iodo-1-(4-methoxyphenyl)ethan-1-one (83 mg, 0.30 mmol, 1.5 equiv.), following oxidation procedure **B**. The crude product was purified by FCC (*n*-hexane/EtOAc = 100/0 to 96/4) to afford the title compound **13c** as a yellow solid in 67% yield (40 mg).

MP: 46-48 °C (*n*-hexane/EtOAc); **R$_f$** (85/15 *n*-hexane/EtOAc): 0.40; **IR** (film) ν_{max}/cm^{-1}: 2920, 2851, 1683, 1597, 1256, 1014, 830, 567; **^{1}H NMR** ($CDCl_3$, 500 MHz) δ (ppm): 8.04 – 7.96 (m, 2H), 6.96 – 6.88 (m, 2H), 6.10 – 6.04 (m, 1H), 5.87 (dd, J = 3.1, 1.0 Hz, 1H), 4.20 (s, 2H), 3.86 (s, 3H), 2.63 – 2.49 (m, 1H), 2.04 – 1.92 (m, 2H), 1.83 – 1.72 (m, 2H), 1.72 – 1.65 (m, 1H), 1.41 – 1.27 (m, 4H), 1.27 – 1.16 (m, 1H); **^{13}C NMR** ($CDCl_3$, 125 MHz) δ (ppm): 194.0, 163.7, 160.6, 146.4, 131.1, 129.5, 113.9, 108.4, 103.8, 55.6, 38.6, 37.3, 31.6, 26.3, 26.0; **HRMS** (ESI) mass calculated for $[M+H]^+$ ($C_{19}H_{23}O_3^+$) requires *m/z* 299.1642, found *m/z* 299.1645.

Ethyl 2-(5-cyclohexylfuran-2-yl)acetate (13d)

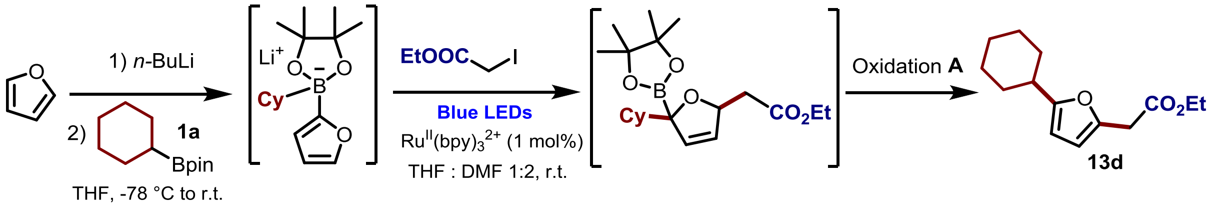

According to the General Procedure, using cyclohexyboronic acid pinacol ester (45 µL, 0.20 mmol, 1.0 equiv.) and ethyl iodoacetate (64 mg, 36 µL, 0.30 mmol, 1.5 equiv.), following oxidation procedure **A**. The crude product was purified by FCC (pentane/Et_2O = 100/0 to 96/4) to afford the title compound **13d** as a colourless oil in 68% yield (32 mg).

R$_f$ (*n*-hexane/EtOAc 95:5): 0.3; **IR** (film) ν_{max}/cm^{-1}: 2927, 2854, 1741, 1141, 1016, 783; **^{1}H NMR** ($CDCl_3$, 500 MHz) δ (ppm): 6.14 – 6.03 (m, 1H), 5.87 (dd, J = 3.1, 1.0 Hz, 1H), 4.17 (q, J = 7.1 Hz, 2H), 3.62 (s, 2H), 2.63 – 2.54 (m, 1H), 2.07 – 1.94 (m, 2H), 1.86 – 1.73 (m, 2H), 1.73 – 1.63 (m, 1H), 1.41 – 1.30 (m, 4H), 1.26 (t, J = 7.1 Hz, 3H), 1.30 – 1.17 (m, 1H); **^{13}C NMR** ($CDCl_3$, 125 MHz) δ (ppm): 169.8, 160.7, 145.6, 108.2, 103.6, 61.2, 37.3, 34.5, 31.7,

26.3, 26.1, 14.3; **HRMS** (ESI) mass calculated for $[M+Na]^+$ ($C_{14}H_{20}NaO_3^+$) requires *m/z* 259.1305, found *m/z* 259.1315.

2-(5-Cyclohexylfuran-2-yl)acetamide (13e)

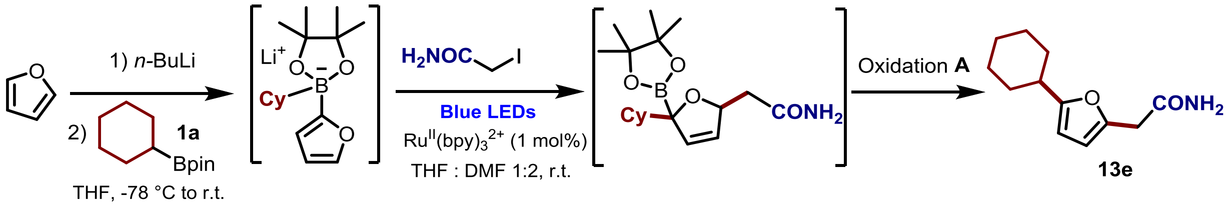

According to the General Procedure, using cyclohexyboronic acid pinacol ester (45 µL, 0.20 mmol, 1.0 equiv.) and iodoacetamide (55 mg, 0.30 mmol, 1.5 equiv.), following oxidation procedure **A**. The crude product was purified by FCC (pentane/EtOAc = 100/0 to 40/60) to afford the title compound **13e** as a white solid in 51% yield (21 mg).

MP: 137-139 °C (*n*-hexane/EtOAc); **R$_f$** (*n*-hexane/EtOAc 70:30): 0.36; **IR** (film) ν_{max}/cm^{-1}: 3354, 3181, 2928, 2851, 1663, 1633, 1413, 1394, 1297, 1017, 972, 891, 779, 649, 404; **^{1}H NMR** ($CDCl_3$, 500 MHz) δ (ppm): 6.11 (d, J = 3.1 Hz, 1H), 5.90 (dd, J = 3.1, 1.0 Hz, 1H), 5.70 (bs, 1H), 5.60 (bs, 1H), 3.57 (s, 2H), 2.66 – 2.50 (m, 1H), 2.07 – 1.93 (m, 2H), 1.87 – 1.65 (m, 3H), 1.43 – 1.29 (m, 4H), 1.27 – 1.23 (m, 1H); **^{13}C NMR** ($CDCl_3$, 125 MHz) δ (ppm): 171.7, 161.4, 146.4, 109.1, 103.9, 37.3, 36.2, 31.6, 26.2, 26.0; **HRMS** (ESI) mass calculated for $[M+H]^+$ ($C_{12}H_{18}NO_2^+$) requires *m/z* 208.1332, found *m/z* 208.1336.

2-Cyclohexyl-5-((phenylsulfonyl)methyl)furan (13f)

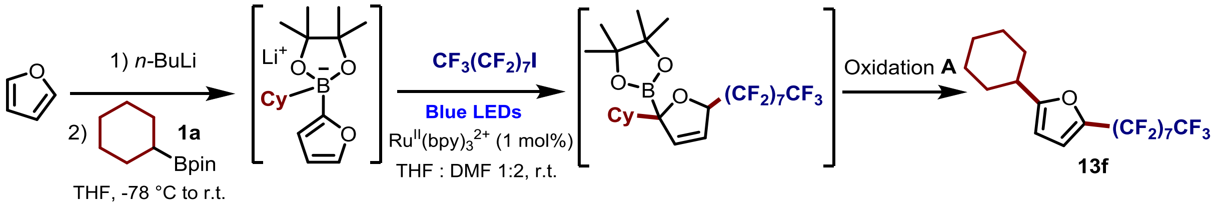

According to the General Procedure, using cyclohexyboronic acid pinacol ester (45 µL, 0.20 mmol, 1.0 equiv.) and perfluorooctyl iodide (164 mg, 79 µL, 0.30 mmol, 1.5 equiv.), following oxidation procedure **A** doubling the load of iodine and potassium acetate. The crude product was purified by FCC (*n*-hexane/EtOAc = 100/0 to 99/1) to afford the title compound **13f** as a colourless oil in 54% yield (61 mg).

R$_f$ (*n*-hexane/EtOAc 95:5): 0.91; **IR** (film) ν_{max}/cm^{-1}: 2933, 2860, 1548, 1200, 1145, 734, 723, 557, 530; **^{1}H NMR** ($CDCl_3$, 500 MHz) δ (ppm): 6.73 (dt, J = 3.4, 1.3 Hz, 1H), 6.07 (dd, J = 3.4, 0.9 Hz, 1H), 2.74 – 2.60 (m, 1H), 2.08 – 1.95 (m, 2H), 1.89 – 1.65 (m, 3H), 1.46 – 1.30

(m, 4H), 1.29 – 1.26 (m, 1H); ^{19}**F NMR** (470 MHz, $CDCl_3$) δ -80.86 (t, J = 10.0 Hz, 3F), -110.7 – -111.7 (m, 2F), -121.8 – -122.1 (m, 6F), -122.5 – -123.2 (m, 4F), -125.9 – -126.6 (m, 2F); ^{13}**C NMR** ($CDCl_3$, 125 MHz) δ (ppm): 165.1, 138.9 (t, J = 33.5 Hz), 114.6, 104.5, 37.3, 31.2, 26.1, 25.9 The signals of the carbons of $(CF_2)_7CF_3$ group could not be detected due to signal splitting and long relaxation delay; **HRMS** (ESI) mass calculated for $[M+H]^+$ ($C_{18}H_{13}OF_{17}^+$) requires *m/z* 569.0768, found *m/z* 569.0777.

2-Cyclohexyl-5-((phenylsulfonyl)methyl)furan (13g)

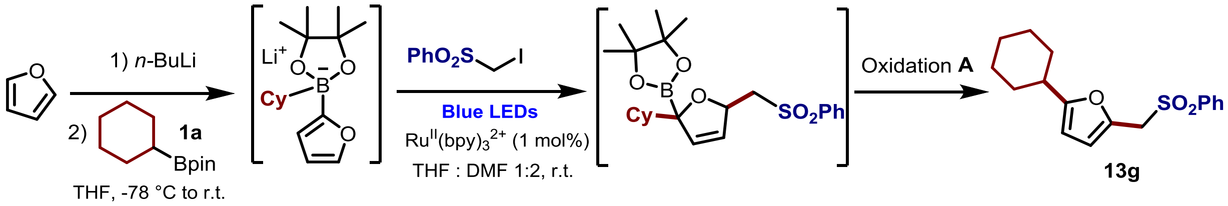

According to the General Procedure, using cyclohexyboronic acid pinacol ester (45 μL, 0.20 mmol, 1.0 equiv.) and iodomethyl phenylsulfone (227 mg, 0.800 mmol, 4.00 equiv.), following oxidation procedure **A**. The crude product was purified by FCC (*n*-hexane/EtOAc = 100/0 to 85/15) to afford the title compound **13g** as a white solid in 64% yield (39 mg).

MP: 47 – 49 °C (*n*-hexane/EtOAc); $\mathbf{R_f}$ (*n*-hexane/EtOAc 70:30): 0.58; **IR** (film) ν_{max}/cm^{-1}: 2934, 2851, 1447, 1304, 1149, 796, 527; ^{1}**H NMR** ($CDCl_3$, 500 MHz) δ (ppm): 7.73 – 7.68 (m, 2H), 7.64 – 7.58 (m, 1H), 7.51 – 7.45 (m, 2H), 6.19 (d, J = 3.2 Hz, 1H), 5.87 (dd, J = 3.2, 1.0 Hz, 1H), 4.37 (s, 2H), 2.42 (tt, J = 10.8, 3.2 Hz, 1H), 1.84 – 1.76 (m, 2H), 1.76 – 1.69 (m, 2H), 1.69 – 1.62 (m, 1H), 1.33 – 1.12 (m, 5H): ^{13}**C NMR** ($CDCl_3$, 125 MHz) δ (ppm): 162.6, 140.0, 138.6, 133.8, 129.1, 128.6, 112.8, 104.5, 56.3, 37.2, 31.4, 26.1, 25.9; **HRMS** (ESI) mass calculated for $[M+Na]^+$ ($C_{17}H_{20}BNaO_3S^+$) requires *m/z* 327.1025, found *m/z* 327.1019.

2-(5-(*tert*-Butyl)furan-2-yl)acetonitrile (13h)

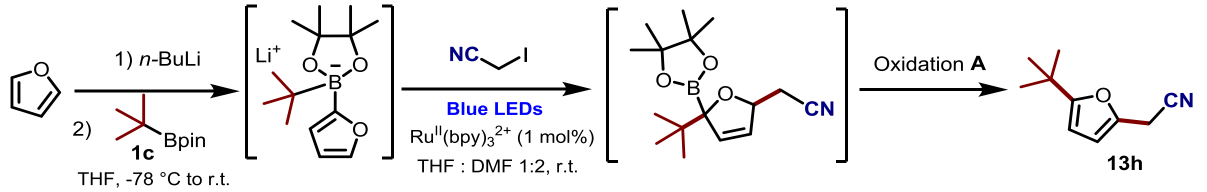

According to the General Procedure, using *tert*-butylboronic acid pinacol ester (44 μL, 0.20 mmol, 1.0 equiv.) and iodoacetonitrile (50 mg, 22 μL, 0.30 mmol, 1.5 equiv.), following oxidation procedure **A**, doubling the loading of iodine and potassium acetate and stirring the reaction for 3 h. The crude product was purified by FCC (pentane/Et_2O = 100/0 to 96/4) to afford the title compound **13h** as a colourless oil in 45% yield (15 mg). Analysis of the crude reaction mixture by NMR using CH_2Br_2 as internal standard (14 μL, 0.20 mmol, 1.0 equiv.) revealed a 64% NMR yield. The discrepancy between NMR yield and isolated yield is due to the volatility of the compound.

R_f (85/15 *n*-hexane/EtOAc): 0.54; **IR** (film) ν_{max}/cm^{-1}: 2968, 2871, 2256, 1557, 1462, 1362, 1277, 1179, 1124, 1016, 973, 787, 749; **^{1}H NMR** ($CDCl_3$, 400 MHz) δ (ppm): 6.17 (dt, J = 3.2, 1.0 Hz, 1H), 5.90 (d, J = 3.2 Hz, 1H), 3.73 (d, J = 1.0 Hz, 2H), 1.26 (s, 9H); **^{13}C NMR** ($CDCl_3$, 100 MHz) δ (ppm): 165.3, 140.7, 116.0, 108.8, 103.2, 32.8, 29.1, 17.7; **HRMS** (ESI) mass calculated for $[M+Na]^+$ ($C_{10}H_{13}NNaO^+$) requires *m/z* 186.0889, found *m/z* 186.0887.

2-(5-Phenethylfuran-2-yl)acetonitrile (13i)

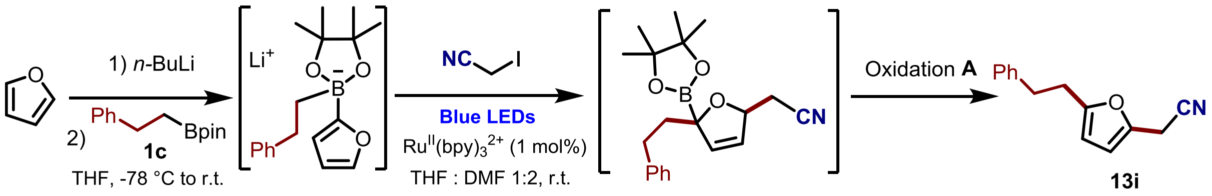

According to the General Procedure, using phenethylboronic acid pinacol ester **1c** (46 mg, 0.20 mmol, 1.0 equiv.) and iodoacetonitrile (50 mg, 22 μL, 0.30 mmol, 1.5 equiv.), following oxidation procedure **A**. The crude product was purified by FCC (*n*-hexane/EtOAc = 100/0 to 94/6) to afford the title compound **13i** as a yellow oil in 76% yield (32 mg).

R_f (85/15 *n*-hexane/EtOAc): 0.33; **IR** (film) ν_{max}/cm^{-1}: 3028, 2927, 2256, 1604, 1563, 1497, 1454, 1018, 788, 745, 698, 496; **^{1}H NMR** ($CDCl_3$, 500 MHz) δ (ppm): 7.33 – 7.27 (m, 2H), 7.24 – 7.16 (m, 3H), 6.19 (dt, J = 3.2, 1.1 Hz, 1H), 5.95 – 5.91 (m, 1H), 3.72 (d, J = 1.1 Hz, 2H), 3.00 – 2.88 (m, 4H); **^{13}C NMR** ($CDCl_3$, 125 MHz) δ (ppm): δ 156.3, 141.1, 141.0, 128.5,

128.4, 126.3, 115.8, 109.2, 106.6, 34.3, 30.0, 17.7; **HRMS** (ESI) mass calculated for $[M+Na]^+$ ($C_{14}H_{13}NNaO^+$) requires *m/z* 234.0889, found *m/z* 234.0896.

(*R*)-2-(5-(1-Phenylethyl)furan-2-yl)acetonitrile (13j)

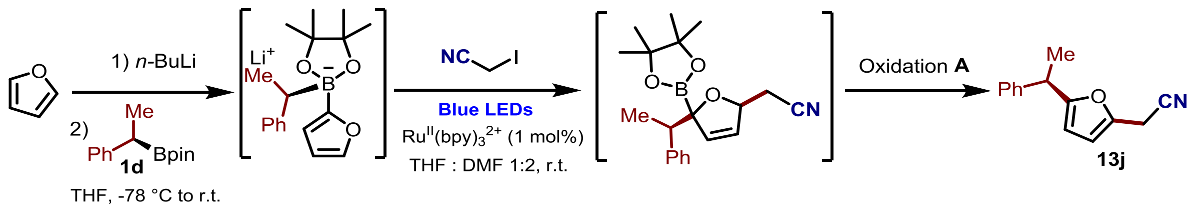

According to the General Procedure, using boronic ester **1d** (46 mg, 0.20 mmol, 1.0 equiv.) and iodoacetonitrile (50 mg, 22 µL, 0.30 mmol, 1.5 equiv.), following oxidation procedure **A**. The crude product was purified by FCC (*n*-hexane/EtOAc = 100/0 to 96/4) to afford the title compound **13j** as a colourless oil in 71% yield (30 mg). The enantiomeric ratio was determined to be 97:3 by HPLC analysis on a Daicel Chiralpak IB column, 98:2 hexane/i-PrOH, flow rate 0.80 mL/min, λ = 240 nm: τ major = 11.65 min, τ minor = 12.4 min. HPLC 97:3 e.r. identical to the one obtained from analysis of the corresponding boronic ester starting material.

R_f (85/15 *n*-hexane/EtOAc): 0.43; **IR** (film) ν_{max}/cm^{-1}: 2973, 2932, 2255, 1558, 1493, 1452, 1177, 1016, 971, 793, 699, 553; $[\alpha]_D^{20}$= -9.6 (c = 0.022 g/ml, $CHCl_3$); **^{1}H NMR** ($CDCl_3$, 500 MHz) δ (ppm): 7.26 – 7.19 (m, 2H), 7.17 – 7.09 (m, 3H), 6.14 (d, J = 3.2 Hz, 1H), 5.91 (d, J = 3.2 Hz, 1H), 4.00 (q, J = 7.2 Hz, 1H), 3.59 (s, 2H), 1.49 (d, J = 7.2 Hz, 3H). **^{13}C NMR** ($CDCl_3$, 125 MHz) δ (ppm): 159.9, 143.7, 141.7, 128.7, 127.4, 126.8, 115.8, 109.0, 106.3, 39.3, 20.5, 17.7; **HRMS** (ESI) mass calculated for $[M+Na]^+$ ($C_{14}H_{13}NNaO^+$) requires *m/z* 234.0889, found *m/z* 234.0897.

2-(5-((1*R*,2*S*,5*R*)-2-Isopropyl-5-methylcyclohexyl)furan-2-yl)acetonitrile (13k)

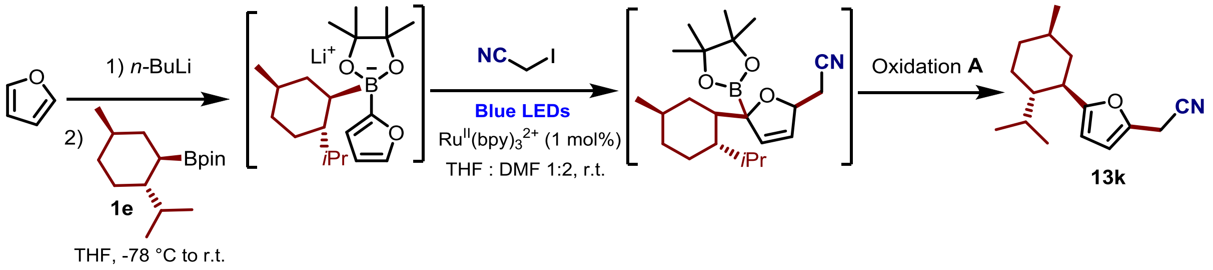

According to the General Procedure, using boronic ester **1e** (53 mg, 0.20 mmol, 1.0 equiv.) and iodoacetonitrile (50 mg, 22 µL, 0.30 mmol, 1.5 equiv.), following oxidation procedure **A**, stirring the reaction for 2 hours. The crude product was purified by FCC (*n*-hexane/EtOAc =

100/0 to 96/4) to afford the title compound **13k** as a yellow oil in 82% yield (40 mg). Only one diastereoisomer was detected by NMR analysis of the crude reaction mixture (> 95:5 d.r.).

R$_f$ (85/15 *n*-hexane/EtOAc): 0.56; **IR** (film) ν_{max}/cm^{-1}: 2954, 2918, 2870, 2256, 1563, 1455, 1368, 1015, 972, 785; $[\alpha]_D^{20}$= –39.5 (c = 0.0076 g/mL, $CHCl_3$); **^{1}H NMR** ($CDCl_3$, 500 MHz) δ (ppm): 6.19 (dt, J = 3.1, 1.0 Hz, 1H), 5.91 (d, J = 3.1 Hz, 1H), 3.72 (d, J = 1.0 Hz, 2H), 2.53 (ddd, J = 12.2, 11.1, 3.4 Hz, 1H), 1.84 – 1.74 (m, 2H), 1.70 (dq, J = 12.7, 3.2 Hz, 1H), 1.49 – 1.37 (m, 3H), 1.25 – 1.20 (m, 1H), 1.15 – 1.02 (m, 1H), 1.02 – 0.93 (m, 1H), 0.90 (d, J = 6.5 Hz, 3H), 0.84 (d, J = 6.9 Hz, 3H), 0.71 (d, J = 6.9 Hz, 3H); **^{13}C NMR** ($CDCl_3$, 125 MHz) δ (ppm): 160.8, 140.5, 115.9, 108.9, 105.6, 46.8, 42.1, 41.0, 35.0, 33.0, 28.5, 24.9, 22.5, 21.3, 17.8, 15.9; **HRMS** (ESI) mass calculated for $[M+Na]^+$ ($C_{16}H_{23}NNaO^+$) requires *m/z* 268.1672, found *m/z* 268.1675.

2-(2-Cyclohexyl-1-methyl-1H-indol-3-yl)acetonitrile (14a)

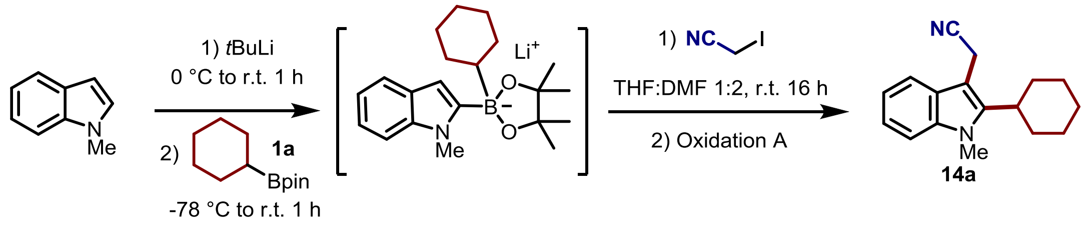

According to the General Procedure, using cyclohexyboronic acid pinacol ester (45 μL, 0.20 mmol, 1.0 equiv.) and iodoacetonitrile (50 mg, 22 μL, 0.30 mmol, 1.5 equiv.), following oxidation procedure **A**. The crude product was purified by FCC (*n*-hexane/EtOAc = 100/0 to 95/5) to afford the title compound **14a** as a yellowish solid in 70% yield (35 mg).

MP: 64 – 66 °C (*n*-hexane/EtOAc); **R$_f$** (85/15 *n*-hexane/EtOAc): 0.35; **IR** (film) ν_{max}/cm^{-1}: 2923, 2854, 2240, 1546, 1474, 1414, 1359, 1133, 747, 552; **^{1}H NMR** ($CDCl_3$, 500 MHz) δ (ppm): 7.56 (ddd, J = 8.2, 7.8, 1.0 Hz, 1H), 7.30 (dt, J = 8.2, 1.0 Hz, 1H), 7.24 (ddd, J = 8.2, 7.0, 1.0 Hz, 1H), 7.17 (ddd, J = 7.8, 7.0, 1.0 Hz, 1H), 3.90 (s, 2H), 3.76 (s, 3H), 2.93 (tt, J = 12.5, 3.2 Hz, 1H), 2.02 – 1.89 (m, 4H), 1.90 – 1.74 (m, 3H), 1.54 – 1.32 (m, 3H); **^{13}C NMR** ($CDCl_3$, 125 MHz) δ (ppm): 142.2, 136.6, 127.1, 121.8, 119.9, 118.8, 117.3, 109.3, 98.9, 37.3, 31.9, 30.5, 27.2, 26.1, 13.8; **HRMS** (ESI) mass calculated for $[M+Na]^+$ ($C_{17}H_{20}N_2Na^+$) requires *m/z* 275.1519, found *m/z* 275.1519.

2-(2-Cyclohexyl-1-methyl-1H-indol-3-yl)-1-phenylethan-1-one (14b)

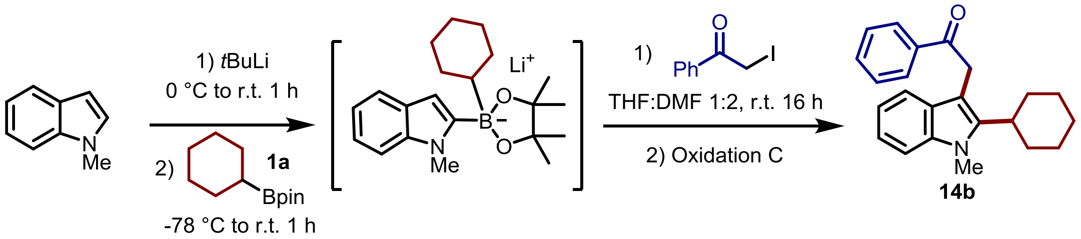

According to the General Procedure, using cyclohexyboronic acid pinacol ester (45 µL, 0.20 mmol, 1.0 equiv.) and iodoacetophenone (74 mg, 0.30 mmol, 1.5 equiv.), following oxidation procedure **C**. The crude product was purified by FCC (*n*-hexane/EtOAc = 100/0 to 98/2) to afford the title compound **14b** as a yellowish solid in 74% yield (49 mg).

MP: 120 – 122 °C (*n*-hexane/EtOAc); **R**$_f$ (85/15 *n*-hexane/EtOAc): 0.46; **IR** (film) ν_{max}/cm^{-1}: 2926, 2852, 1741, 1693, 1474, 1446, 1200, 1015, 783, 739, 687, 623, 562; ^{1}**H NMR** ($CDCl_3$, 400 MHz) δ (ppm): 8.17 – 8.01 (m, 2H), 7.66 – 7.53 (m, 1H), 7.53 – 7.38 (m, 3H), 7.34 – 7.22 (m, 1H), 7.22 – 7.13 (m, 1H), 7.13 – 7.00 (m, 1H), 4.51 (s, 2H), 3.78 (s, 3H), 2.96 (ddt, J = 13.9, 11.6, 3.4 Hz, 1H), 1.98 – 1.66 (m, 7H), 1.50 – 1.21 (m, 3H); ^{13}**C NMR** ($CDCl_3$, 100 MHz) δ (ppm): 198.3, 142.2, 137.3, 137.0, 133.0, 128.7, 128.4, 128.3, 121.1, 119.3, 117.9, 109.0, 103.4, 37.4, 35.5, 31.8, 30.9, 27.3, 26.3; **HRMS** (ESI) mass calculated for $[M+Na]^+$ ($C_{23}H_{25}NNaO^+$) requires *m/z* 354.1828, found *m/z* 354.1835.

Ethyl 2-(2-cyclohexyl-1-methyl-1H-indol-3-yl)acetate (14c)

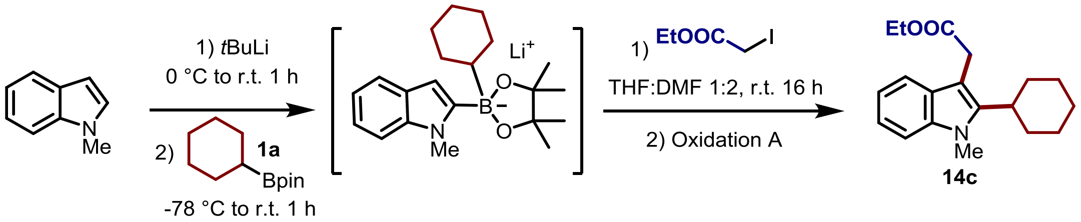

According to the General Procedure, using cyclohexyboronic acid pinacol ester (45 µL, 0.20 mmol, 1.0 equiv.) and ethyl iodoacetate (64 mg, 36 µL, 0.30 mmol, 1.5 equiv.), following oxidation procedure **A**. The crude product was purified by FCC (*n*-hexane/EtOAc = 100/0 to 96/4) to afford the title compound **14c** as yellowish solid in 72% yield (43 mg).

MP: 38 – 40 °C (*n*-hexane/EtOAc); **R**$_f$ (85/15 *n*-hexane/EtOAc): 0.51; **IR** (film) ν_{max}/cm^{-1}: 2930, 2850, 1733, 1473, 1362, 1154, 1032, 743; ^{1}**H NMR** ($CDCl_3$, 500 MHz) δ (ppm): 7.57 (dt, J = 7.9, 1.0 Hz, 1H), 7.26 (d, J = 8.2 Hz, 1H), 7.19 (ddd, J = 8.2, 6.9, 1.0 Hz, 1H), 7.11 (ddd, J = 7.9, 6.9, 1.0 Hz, 1H), 4.14 (q, J = 7.1 Hz, 2H), 3.84 (s, 2H), 3.77 (s, 3H), 2.97 (tt, J =

12.4, 3.3 Hz, 1H), 1.98 – 1.88 (m, 4H), 1.88 – 1.77 (m, 3H), 1.51 – 1.32 (m, 3H), 1.25 (t, J = 7.1 Hz, 3H); **^{13}C NMR** ($CDCl_3$, 125 MHz) δ (ppm): 172.4, 142.1, 136.7, 128.2, 121.1, 119.3, 118.2, 108.8, 103.5, 60.7, 37.3, 31.9, 31.1, 30.7, 27.4, 26.3, 14.4; **HRMS** (ESI) mass calculated for $[M+Na]^+$ ($C_{19}H_{25}NNaO_2^+$) requires *m/z* 322.1777, found *m/z* 322.1792.

2-(2-Cyclohexyl-1-methyl-1H-indol-3-yl)acetamide (14d)

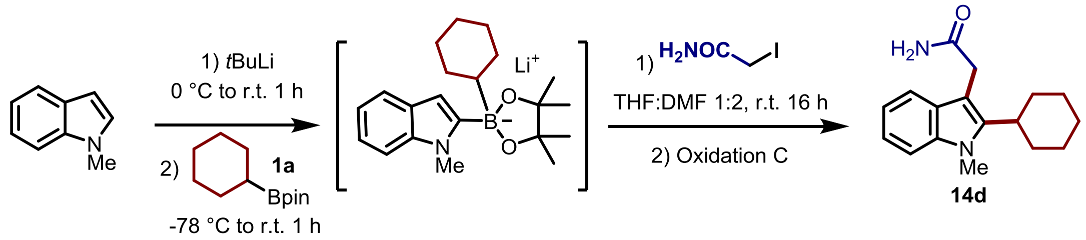

According to the General Procedure, using cyclohexyboronic acid pinacol ester (45 μL, 0.20 mmol, 1.0 equiv.) and iodoacetamide (55 mg, 0.30 mmol, 1.5 equiv.), following oxidation procedure **C**. The crude product was purified by FCC (*n*-hexane/EtOAc = 100/0 to 40/60) to afford the title compound **14d** as a white solid in 54% yield (29 mg).

MP: 184 – 185 °C (*n*-hexane/EtOAc); **R_f** (40/60 *n*-hexane/EtOAc): 0.29; **IR** (film) ν_{max}/cm^{-1}: 3441, 3190, 2925, 2851, 1633, 1473, 1381, 803, 735, 633; **^{1}H NMR** ($CDCl_3$, 500 MHz) δ (ppm): 7.49 (d, J = 7.8 Hz, 1H), 7.29 (d, J = 8.2 Hz, 1H), 7.25 – 7.18 (m, 1H), 7.13 (t, J = 7.8 Hz, 1H), 5.63 (bs, 2H), 3.80 (s, 2H), 3.77 (s, 3H), 2.91 (tt, J = 12.3, 3.4 Hz, 1H), 1.96 – 1.72 (m, 7H), 1.48 – 1.24 (m, 3H); **^{13}C NMR** ($CDCl_3$, 125 MHz) δ (ppm): 175.0, 142.7, 136.8, 127.9, 121.6, 119.8, 117.8, 109.2, 103.8, 37.2, 32.8, 32.0, 30.5, 27.2, 26.0; **HRMS** (ESI) mass calculated for $[M+H]^+$ ($C_{17}H_{23}N_2O^+$) requires *m/z* 271.1805, found *m/z* 271.1807.

2-(2-Cyclohexyl-1-methyl-1H-indol-3-yl)acetamide (14e)

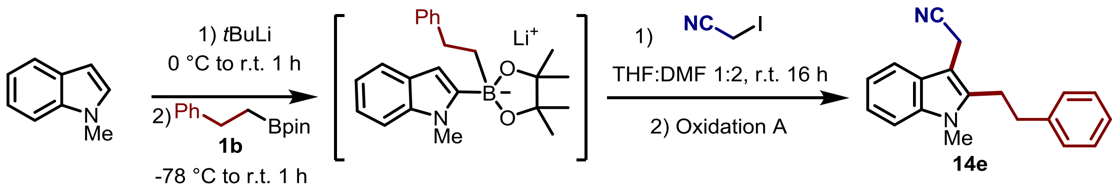

According to the General Procedure, using phenethylboronic acid pinacol ester (46 mg, 0.20 mmol, 1.0 equiv.) and iodoacetonitrile (50 mg, 22 μL, 0.30 mmol, 1.5 equiv.), following oxidation procedure **A**. The crude product was purified by FCC (*n*-hexane/EtOAc = 100/0 to 96/4) to afford the title compound **14e** as a yellow oil in 67% yield (37 mg).

R$_f$ (85/15 *n*-hexane/EtOAc): 0.39; **IR** (film) ν_{max}/cm^{-1}: 2933, 2244, 1563, 1472, 1454, 1369, 739, 699, 556, 495, 439; **^{1}H NMR** ($CDCl_3$, 500 MHz) δ (ppm): 7.57 (dt, J = 7.8, 1.0 Hz, 1H), 7.32 – 7.22 (m, 5H), 7.21 – 7.14 (m, 1H), 7.11 – 7.06 (m, 2H), 3.61 (s, 3H), 3.45 (s, 2H), 3.11 (t, J = 7.4 Hz, 2H), 2.93 (t, J = 7.4 Hz, 2H); **^{13}C NMR** ($CDCl_3$, 125 MHz) δ (ppm): 140.4, 137.3, 136.7, 128.8, 128.7, 126.7, 126.4, 121.8, 120.0, 118.4, 117.7, 109.3, 100.2, 35.9, 29.7, 27.1, 12.9; **HRMS** (ESI) mass calculated for $[M+Na]^+$ ($C_{19}H_{18}N_2Na^+$) requires *m/z* 297.1362, found *m/z* 297.1368.

(*R*)-2-(1-Methyl-2-(1-phenylethyl)-1H-indol-3-yl)acetonitrile (14f)

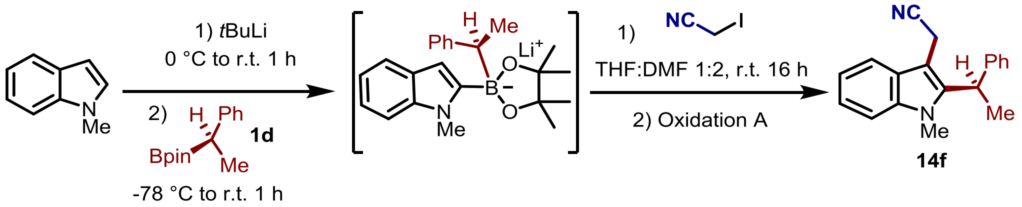

According to the General Procedure, using chiral boronic ester **1d** (46 mg, 0.20 mmol, 1.0 equiv.) and iodoacetonitrile (50 mg, 22 μL, 0.30 mmol, 1.5 equiv.), following oxidation procedure **A**. The crude product was purified by FCC (*n*-hexane/EtOAc = 100/0 to 96/4) to afford the title compound **14f** as a colourless oil in 73% yield (40 mg).

R$_f$ (91/9 *n*-hexane/EtOAc): 0.13; **IR** (film) ν_{max}/cm^{-1}: 3057, 2973, 2938, 2244, 1601, 1471, 1366, 909, 740, 699; $[\alpha]_D^{20}$= +64.0 (c = 0.005 g/ml, $CHCl_3$); **^{1}H NMR** ($CDCl_3$, 400 MHz) δ (ppm): 7.59 (dt, J = 7.7, 1.0 Hz, 1H), 7.36 – 7.30 (m, 2H), 7.30 – 7.14 (m, 6H), 4.62 (q, J = 7.4 Hz, 1H), 3.70 (d, J = 18.0 Hz, 1H), 3.60 (d, J = 18.0 Hz, 1H), 3.49 (s, 3H), 1.81 (d, J = 7.4 Hz, 3H); **^{13}C NMR** ($CDCl_3$, 125 MHz) δ (ppm): 142.0, 141.2, 136.9, 128.9, 127.2, 126.8, 126.8, 122.1, 120.1, 118.4, 117.7, 109.3, 100.3, 35.3, 30.6, 18.8, 13.3; **HRMS** (ESI) mass calculated for $[M+Na]^+$ ($C_{19}H_{18}N_2Na^+$) requires *m/z* 297.1362, found *m/z* 297.1372.

(*R*)-2-(2-(1-(4-Methoxyphenyl)propan-2-yl)-1-methyl-1H-indol-3-yl)acetonitrile (14g)

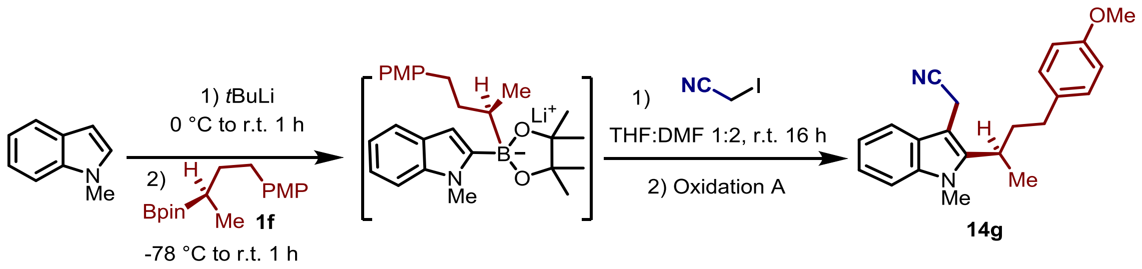

According to the General Procedure, using chiral boronic ester **1f** (58 mg, 0.20 mmol, 1.0 equiv.) and iodoacetonitrile (50 mg, 22 μL, 0.30 mmol, 1.5 equiv.), following oxidation

procedure **A**. The crude product was purified by FCC (*n*-hexane/EtOAc = 100/0 to 96/4) to afford the title compound **14g** as a colourless oil in 93% yield (62 mg).

R_f (91/9 *n*-hexane/EtOAc): 0.16; **IR** (film) ν_{max}/cm^{-1}: 2934, 2244, 1711, 1611, 1512, 1471, 1244, 1178, 1034, 828, 744, 558; $[\alpha]_D^{20}$= +113.0 (c = 0.0018 g/ml, $CHCl_3$); **^{1}H NMR** ($CDCl_3$, 400 MHz) δ (ppm): 7.60 (d, J = 7.6 Hz, 1H), 7.31 (t, J = 7.7 Hz, 1H), 7.29 – 7.24 (m, 1H), 7.20 (ddd, J = 7.6, 6.7, 1.2 Hz, 1H), 7.09 – 6.99 (m, 2H), 6.88 – 6.78 (m, 2H), 3.80 (s, 3H), 3.77 - 3.73 (m, 2H), 3.67 (s, 3H), 3.17 (h, J = 7.4 Hz, 1H), 2.58 (t, J = 7.6 Hz, 2H), 2.26 – 2.02 (m, 2H), 1.47 (d, J = 7.3 Hz, 3H); **^{13}C NMR** ($CDCl_3$, 125 MHz) δ (ppm): 158.0, 141.6, 136.9, 133.3, 129.3, 127.0, 121.8, 120.0, 118.5, 117.5, 114.0, 109.3, 99.5, 55.4, 37.5, 33.3, 30.7, 30.5, 20.0, 13.5; **HRMS** (ESI) mass calculated for $[M+Na]^+$ ($C_{22}H_{24}N_2NaO^+$) requires *m/z* 355.1781, found *m/z* 355.1793.

2-(2-((1*R*,2*S*,5*R*)-2-Isopropyl-5-methylcyclohexyl)-1-methyl-1H-indol-3-yl)acetonitrile (14h)

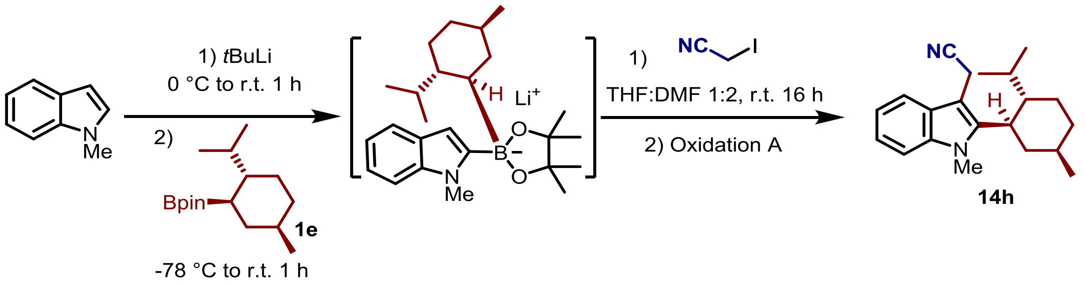

According to the General Procedure, using chiral boronic ester **1e** (53 mg, 0.20 mmol, 1.0 equiv.) and iodoacetonitrile (100 mg, 43.2 µL, 0.600 mmol, 3.00 equiv.), following oxidation procedure **A**. The crude product was purified by preparative TLC (*n*-hexane/EtOAc = 96/4) to afford the title compound **14h** as a colourless oil in 18% yield (11 mg).

R_f (96/4 *n*-hexane/EtOAc): 0.15; **IR** (film) ν_{max}/cm^{-1}: 2952, 2924, 2869, 2246, 1738, 1472, 1363, 908, 764, 739; $[\alpha]_D^{20}$= –41.8 (c = 0.0048 g/ml, $CHCl_3$); **^{1}H NMR** ($CDCl_3$, 400 MHz) δ (ppm): The product is a 1:1 mixture of rotamers, rotameric signals are marked with *: 7.62 – 7.55 (m, 1H), 7.33 – 7.28 (m, 1H), 7.27 – 7.21 (m, 1H), 7.20 – 7.15 (m, 1H), 3.94 (d, *J* = 17.8 Hz, 0.5H)*, 3.87 (d, *J* = 17.9 Hz, 0.5H)*, 3.83 (d, *J* = 17.9 Hz, 0.5H)*, 3.82 (s, 1.5H)*, 3.76 (d, *J* = 18.0 Hz, 0.5H)*, 3.72 (s, 1.5H)*, 2.92 (td, *J* = 12.0, 3.6 Hz, 0.5H)*, 2.85 (td, *J* = 12.0, 3.6 Hz, 0.5H)*, 1.98 – 1.75 (m, 4H), 1.68 – 1.01 (m, 5H), 0.96 (d, *J* = 6.5 Hz, 1.5H)*, 0.95 (d, *J* = 6.6 Hz, 1.5H)*, 0.92 (d, *J* = 7.0 Hz, 1.5H)*, 0.85 (d, *J* = 7.0 Hz, 1.5H)*, 0.70 (d, *J* = 6.9 Hz, 1.5H)*, 0.69 (d, *J* = 6.8 Hz, 1.5H)*; **^{13}C NMR** ($CDCl_3$, 100 MHz) δ (ppm): The two sets

of signals are due to the occurrence of rotamers: 141.2, 140.8, 137.5, 136.4, 127.6, 126.6, 121.8, 121.6, 120.0, 120.0, 118.3, 118.3, 117.7, 117.3, 109.5, 109.0, 100.6, 98.5, 46.4, 45.6, 41.9, 40.9, 40.4, 39.4, 35.4, 35.2, 33.7, 33.6, 32.0, 29.8, 28.7, 28.1, 25.8, 25.3, 22.5, 22.4, 21.6, 21.6, 16.2, 16.0, 14.0, 13.6; **HRMS** (ESI) mass calculated for $[M+Na]^+$ ($C_{21}H_{28}N_2Na^+$) requires *m/z* 331.2145, found *m/z* 331.2152.

As detailed above, the ^{1}H-NMR and ^{13}C-NMR spectra (at r.t.) of compound **14h** are characterized by the presence of two set of signals (for the whole spectrum see section 6). Variable temperature NMR analysis was used to give insights into the origin of this phenomenon. As depicted in Figure S1 the two sets of signals were found to coalesce at 120 °C in DMSO-d_6, suggesting that at 25 °C **14h** is present in solution as two conformers (most likely originating from restricted rotation around C2-C1') that interconvert at a slower rate than the NMR timescale. In figure S1, the relevant section of the spectrum performed at 120 °C clearly shows a single set of signals for the three methyl groups within the menthyl moiety in **14h**, indicating a d.r. >95:5.

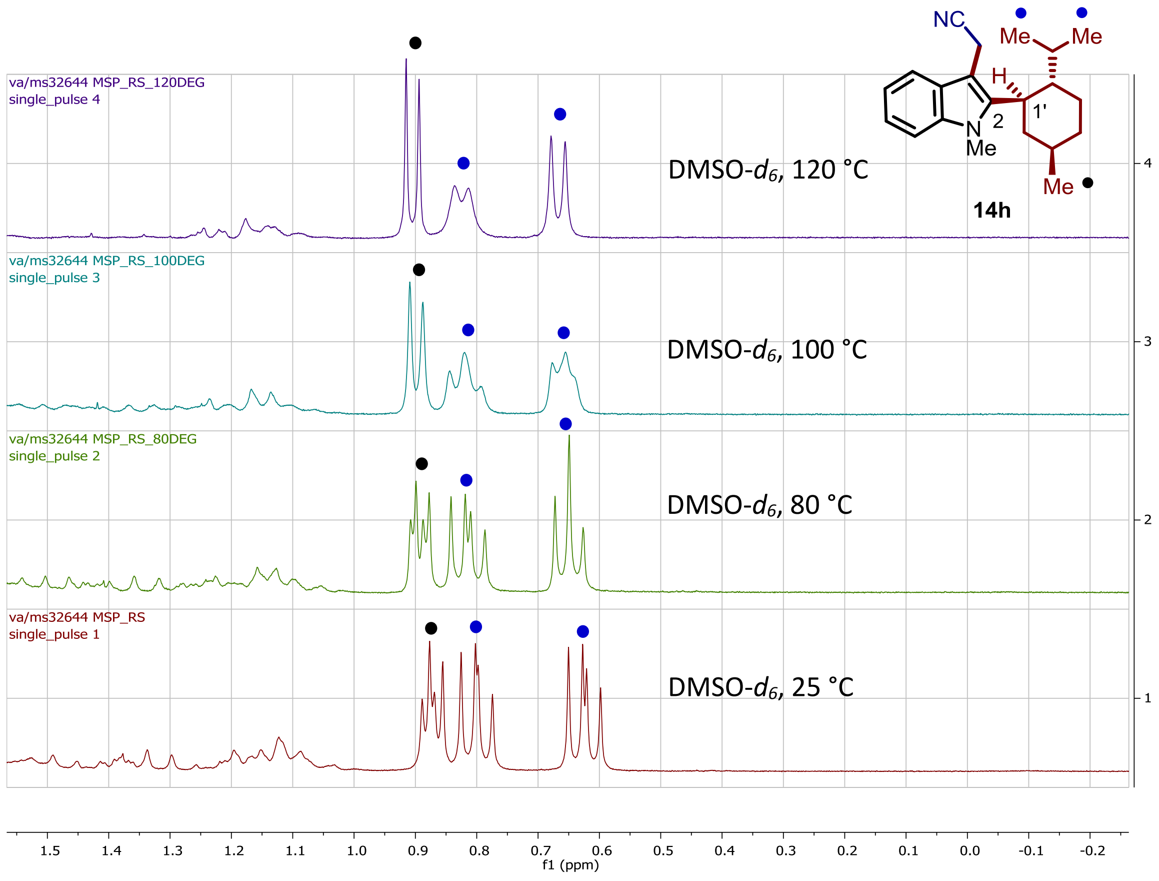

Fig. S1 – Relevant section of the ^{1}H-NMR spectrum for compound **14h** performed at different temperatures in DMSO-d_6. The two sets of signals observed at 25 °C for the Me signals of the menthyl motif coalesce to a single set at 120 °C.

3. Mechanistic Studies

3.1 Synthesis and Characterization of Boronate Complex 2a

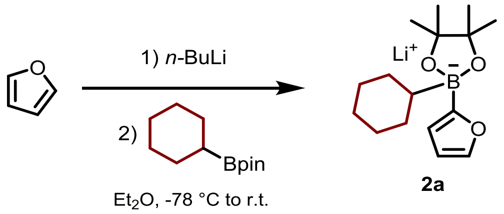

To a -78 °C stirred solution of furan (71.5 mg, 76.4 µL, 1.05 mmol, 1.05 equiv.) in dry Et_2O (10 mL) under a N_2 atmosphere was added dropwise a 1.6 M solution of *n*-butyllithium in hexanes (687 µL – 1.10 mmol, 1.10 equiv.). The solution was left to warm to r.t. and stirred for 30 minutes, after which it was cooled down to -78 °C. Cyclohexylboronic acid pinacol ester (210 mg, 221 µL, 1.00 mmol, 1.00 equiv.) was added dropwise to the cold mixture under a nitrogen atmosphere. The reaction was left to warm to r.t. and stirred for one hour, the formation of a white precipitate was observed. The mixture was then cooled down to 0 °C and filtered under N_2 to collect solid compound **2a**. The boronate complex was washed with 10 mL of cold (0 °C), dry Et_2O to remove impurities and dried under vacuum. Compound **2a** was obtained as a white solid (170 mg, 60% yield).

MP: gradual decomposition above 120 °C; **IR** (film) ν_{max}/cm^{-1}: 2970, 2916, 2844, 1738, 1365, 1275, 984, 764, 750; **^{1}H NMR** (DMSO-d_6, 500 MHz) δ (ppm): 7.28 – 7.19 (m, 1H), 5.99 (dd, J = 2.9, 1.6 Hz, 1H), 5.74 (dd, J = 2.8, 0.7 Hz, 1H), 1.54 – 1.44 (m, 5H), 1.01 – 0.93 (m, 3H), 0.91 (s, 6H), 0.88 (d, J = 5.3 Hz, 1H), 0.80 (s, 6H), 0.77-0.66 (m, 2H); **^{13}C NMR** (DMSO-d_6, 125 MHz) δ (ppm): 138.8, 107.9, 107.6, 76.4, 30.0, 29.2, 28.0, 27.6, 26.6; **^{11}B NMR** (128 MHz, DMSO-d_6) δ (ppm): 5.14; **HRMS** (ESI) mass calculated for $[M]^-$ ($C_{16}H_{26}BO_3^-$) requires *m/z* 277.1983, found *m/z* 277.1973.

3.2 Fluorescence Quenching Experiments

The emission spectra were recorded using a Varian Cary Eclipse fluorimeter, equipped with a Xenon pulse lamp, pulsed frequency 80 Hz, pulse width at half peak height ~ 2 µs, peak power equivalent to 75 kW. 2 mL of a $6.68 \cdot 10^{-4}$ M solution of $Ru(bpy)_3 \cdot 6H_2O$ in dry DMF (spectrophotometric grade) were placed in a Hellma® fluorescence cuvette, 10x10 mm light

path Supersil® quartz, equipped with PTFE lid. The solution was degassed through nitrogen sparging for three minutes, and analysed immediately. The excitation wavelength was fixed at 465 nm (incident light slit regulated to 10 mm), while the emission light was acquired from 515 nm to 700 nm (emission light slit regulated to 10 mm). For quenching data the emission wavelength was fixed to 615 nm.

Different solutions, with different concentration of quencher were prepared and analysed following the procedure detailed above. The concentration of Ru photocatalyst was maintained constant, varying only the quencher concentration.

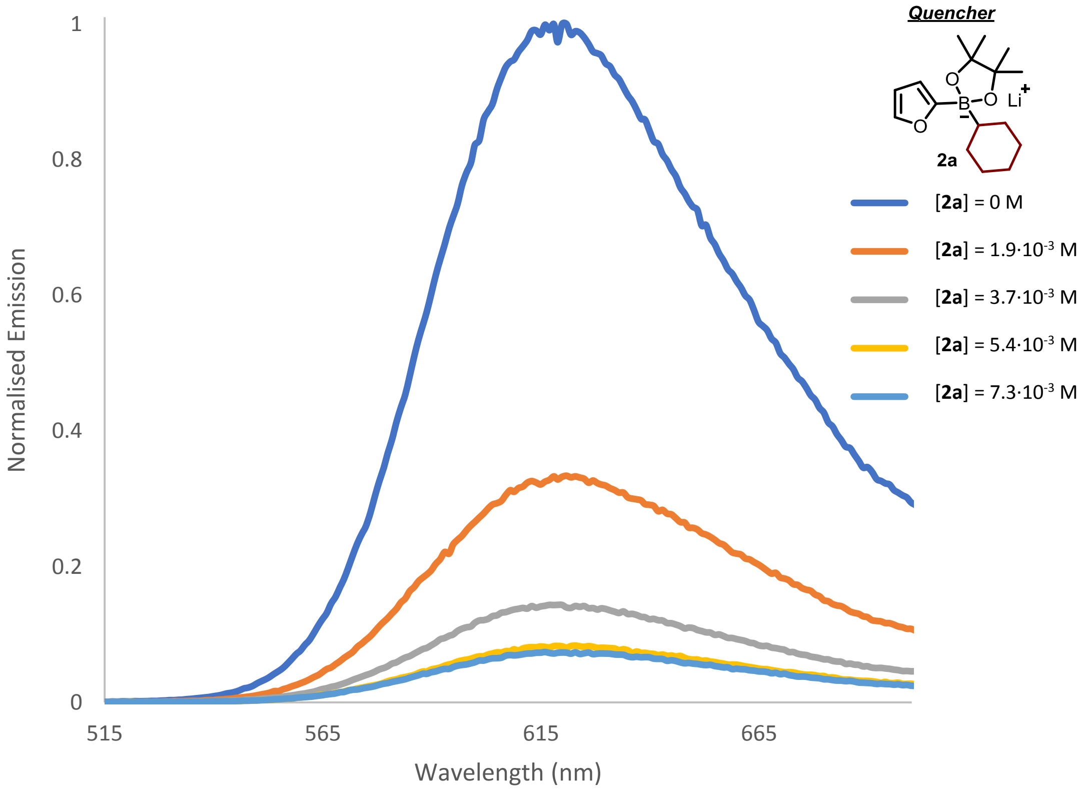

Fig. S2 – Emission spectrum of solutions of Ru(bpy)$_3$·6H_2O (6.68·10^{-4} M in DMF) at different concentrations of boronate complex **2a**.

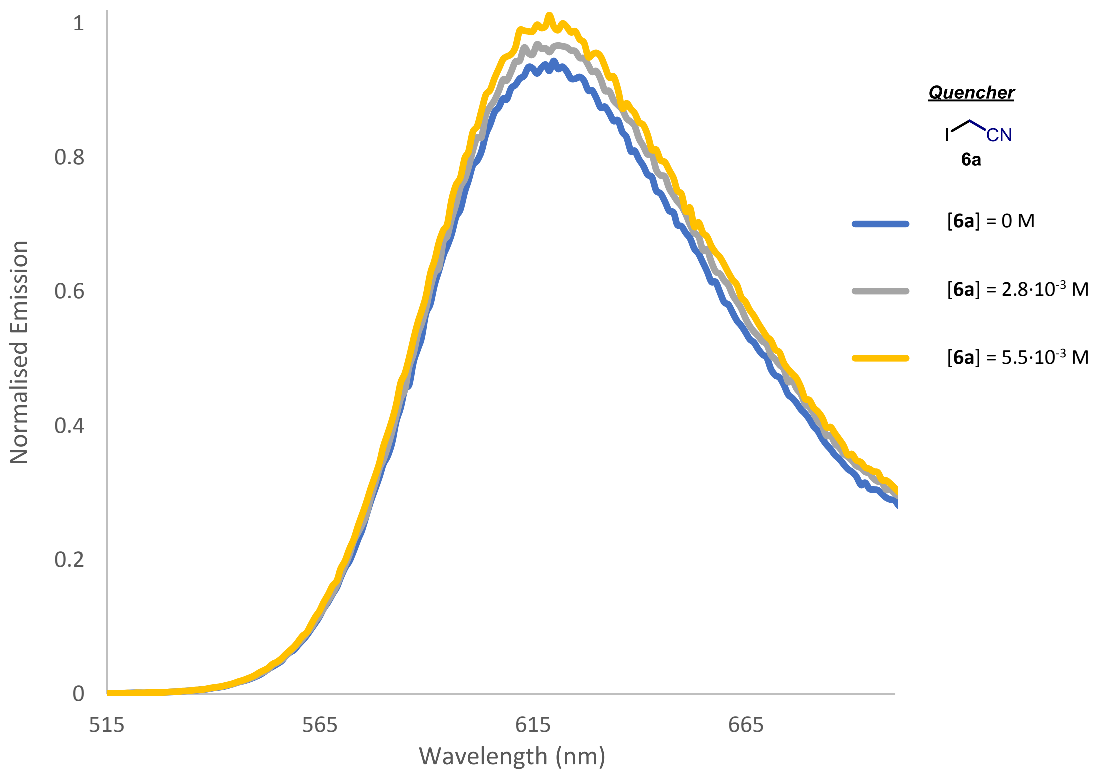

Fig. S3 – Emission spectrum of solutions of Ru(bpy)$_3$·6H_2O (6.68·10^{-4} M in DMF) at different concentrations of iodoacetonitrile **6a**.

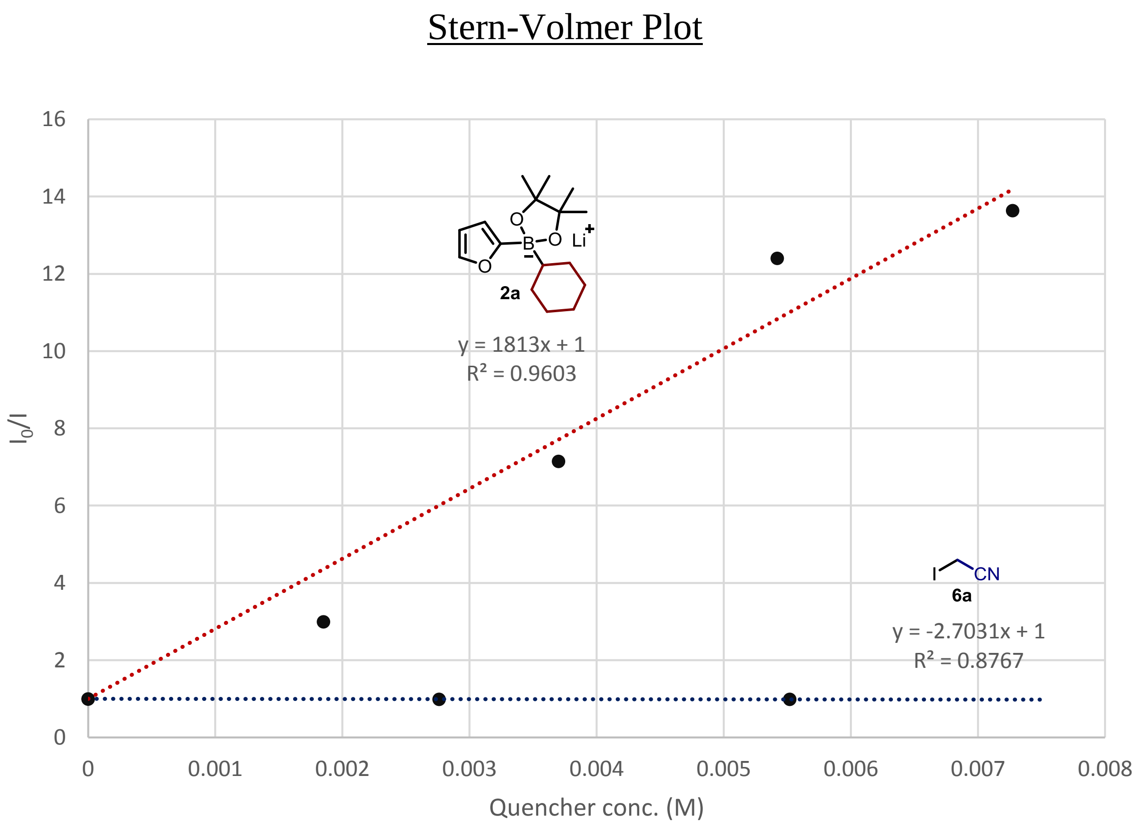

Fig. S4 – Stern-Volmer plot for the quenching studies above. Emission wavelength fixed at 615 nm.

Iodoacetonitrile **6a** is not able to quench the fluorescence of $Ru(bpy)_3 \cdot 6H_2O$ while the boronate complex **2a** is an effective quencher. The Stern-Volmer constant for this quenching is K_{SV} = 1813 M^{-1}.
The excited state lifetime for $Ru(bpy)_3 \cdot 6H_2O$ in DMF at r.t. is $\tau_0 = 912 \cdot 10^{-9}$ s.[8]
From this data it is possible to calculate the bimolecular quenching constant (k_q) using eq. (1).[9]

eq. (1) $$k_q = K_{sv}/\tau_0$$

The excited $Ru(bpy)_3 \cdot 6H_2O$ is quenched by boronate complex **2a** with a bimolecular quenching constant $k_q = 1.98 \cdot 10^9\ M^{-1} \cdot s^{-1}$, which is close to the diffusion limit, generally considered to be approximatively $k_0 \approx 1 \cdot 10^{10}$ for small organic molecules in solution.[9]
In conclusion, the Ru excited state is readily quenched by the boronate complex **2a** while no interactions were observed with iodoacetonitrile **6a**. These data strongly support the mechanism reported in Scheme 4.

3.3 Electrochemical Experiments

Cyclic voltammetry analyses were performed using a Basi Epsilon EC potentiostat. Electrochemical grade tetrabutylammonium hexafluorophosphate (242 mg, 0.625 mmol) was added to a 6.25 mL, 4 mM solution of analyte (**2a** or **6a**) in dry DMF and the solution was vigorously bubbled with argon for 5 minutes under stirring prior to the measurement. The stirring was then stopped, and the solution was allowed to reach quietness, after which the measure was carried out under an argon atmosphere. The anodic/cathodic peak potentials were measured using a glassy carbon working electrode, a platinum wire counter electrode, and a 3 M NaCl Ag/AgCl reference electrode at 200 mV/s scan rate using ferrocene as internal standard. The measurements are reported in graphs vs SCE, considering the conversion SCE = +420 mV Fc/Fc^+.[10]
E_p were obtained from the graphs as the potential corresponding to the maximum current observed. To better evaluate compounds' redox potential, $E_{p/2}$ were used[10] and measured as the potential corresponding to the half value of the maximum current intensity observed.

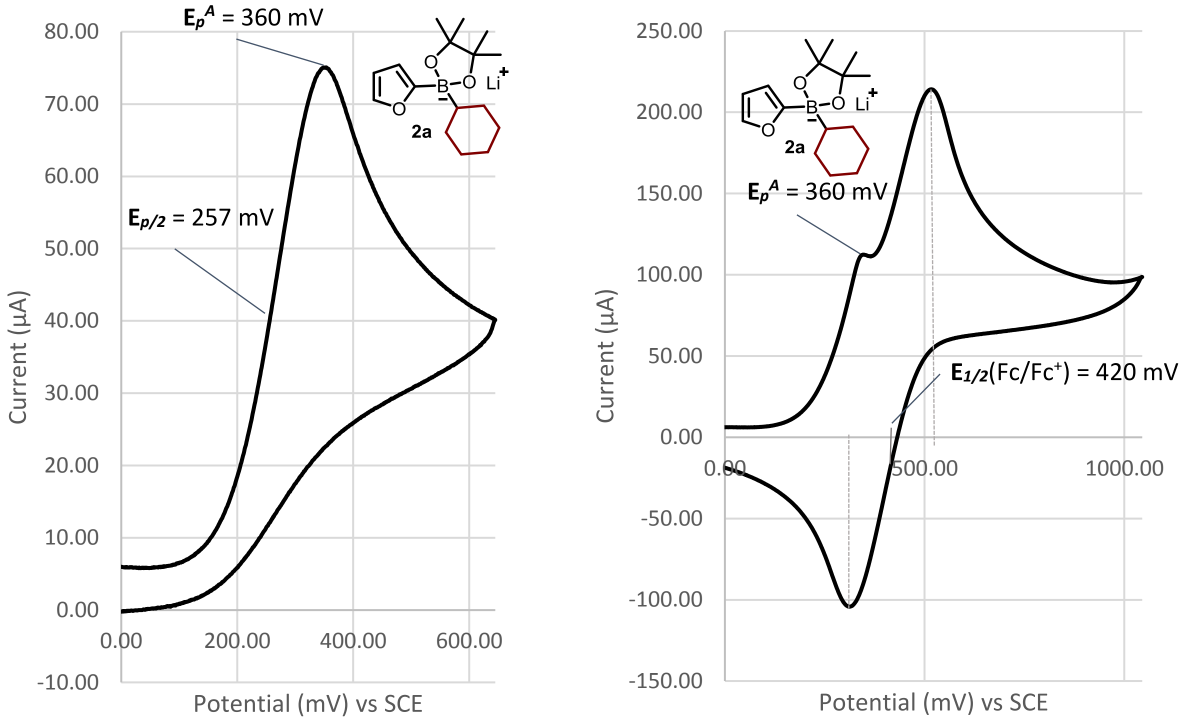

Fig. S5 – Cyclic voltammograms of a 4 mM solution of **2a** (left) and of a 4 mM **2a** in the presence of ferrocene as internal standard (8.6 mM).

The cyclic voltammetry of boronate complex **2a** shows a completely irreversible oxidation wave with E_p^a = +360 mV (vs SCE) and $E_{p/2}$ = +257 mV (vs SCE).

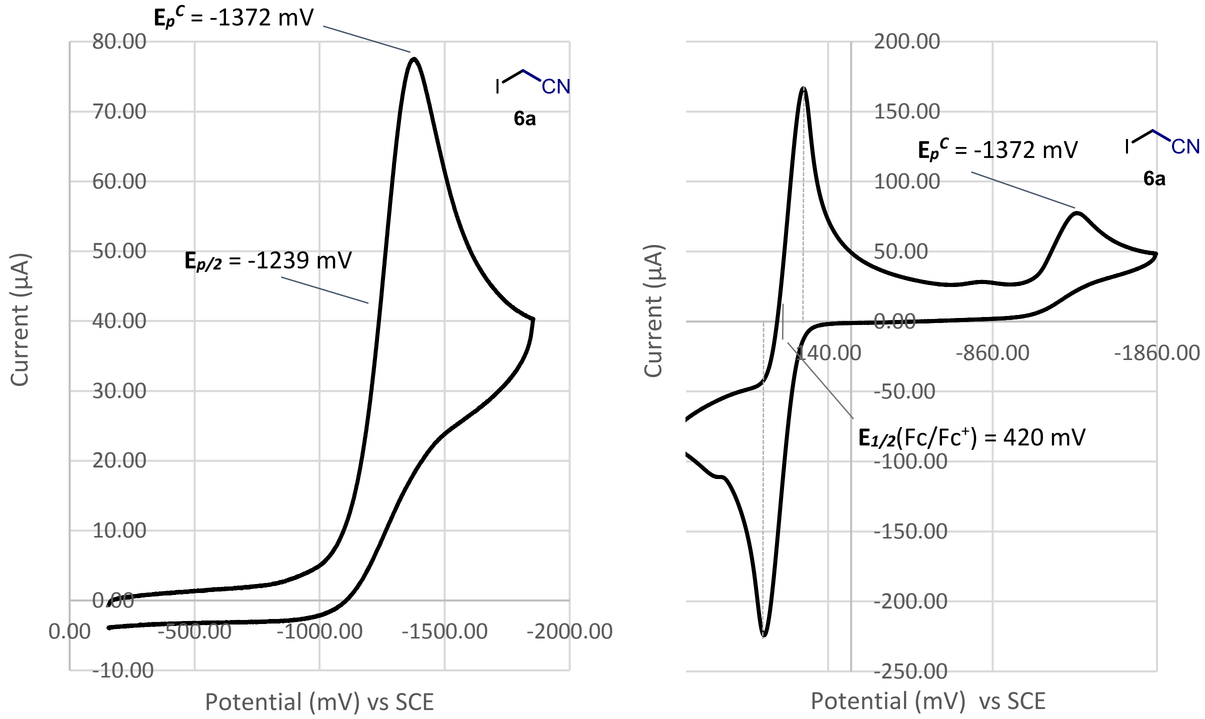

Fig. S6 – Cyclic voltammograms of a 4 mM solution of iodoacetonitrile **6a** (left) and of a 4 mM iodoacetonitrile **6a** in the presence of ferrocene as internal standard (8.6 mM).

The cyclic voltammetry of iodoacetonitrile **6a** shows a completely irreversible reduction wave with E_p^c = -1372 mV (vs SCE) and $E_{p/2}$ = -1239 mV (vs SCE).
The electrochemical proprieties of the compounds are consonant with a scenario in which reductive quenching of Ru^{II*} ($E_0^{II*/I}$ = 770 mV vs SCE) is induced by the boronate complex. The Ru^I ($E_0^{I/II}$ = -1330 mV vs SCE) species acts as a reductant to generate radicals by direct reduction of iodoacetonitrile.

3.4 Quantum Yield Measurements

The quantum yield was measured for the reaction of furan with cyclohexylboronic acid pinacol ester (**1a**) and iodoacetonitrile (**6a**). The reaction was performed in a quartz cuvette (path length: l = 1.0 cm) positioned 5 cm away from a single 0.1 W blue LED (λ_{max} = 450 nm).

Determination of the Photon Flux:

The photon flux of the LED setup was determined using standard ferrioxalate actinometry.[11] A 0.15 M ferrioxalate solution was prepared by dissolving 2.21 g of potassium ferrioxalate trihydrate in 30 mL of 0.05 M aq. H_2SO_4. A buffered phenanthroline solution was prepared by dissolving 50 mg of 1,10-phenanthroline 11.25 g of NaOAc•3H_2O in 50 mL of 0.5 M aq. H_2SO_4. Both solutions were stored in amber bottles in the dark.
Whilst working under red light, 1.0 mL of the 0.15 M ferrioxalate solution was added to a quartz cuvette (l = 1.0 cm). The cuvette was placed 5 cm from a single blue LED and irradiated for specific time internals of between 10 and 60 s. After irradiation, 0.50 mL of the 1,10-phenanthroline solution was added to the cuvette. The mixture was left to stand for approximately 30 minutes before the absorbance at λ = 510 nm was measured by UV/Vis spectroscopy using a Perkin Elmer Lambda 25 UV/Vis Spectrophotometer. The absorbance of a non-irradiated sample was also measured.
The number of moles of Fe^{2+} formed was calculated using:

$$\text{mol Fe}^{2+} = \frac{\text{V} \bullet \Delta\text{A}}{\text{l} \bullet \varepsilon}$$

Where V is the total volume of the solution after the addition of 1,10-phenanthroline (0.0015 L), ΔA is the difference in absorbance at λ = 510 nm between the irradiated and non-irradiated ferrioxalate solutions, l is the optical path length of the irradiation cell (1.0 cm), and ε is the molar absorptivity of the $Fe(phen)_3^{2+}$ complex at λ = 510 nm (11,100 L mol^{-1} cm^{-1}).

The moles of Fe^{2+} were plotted as a function of time:

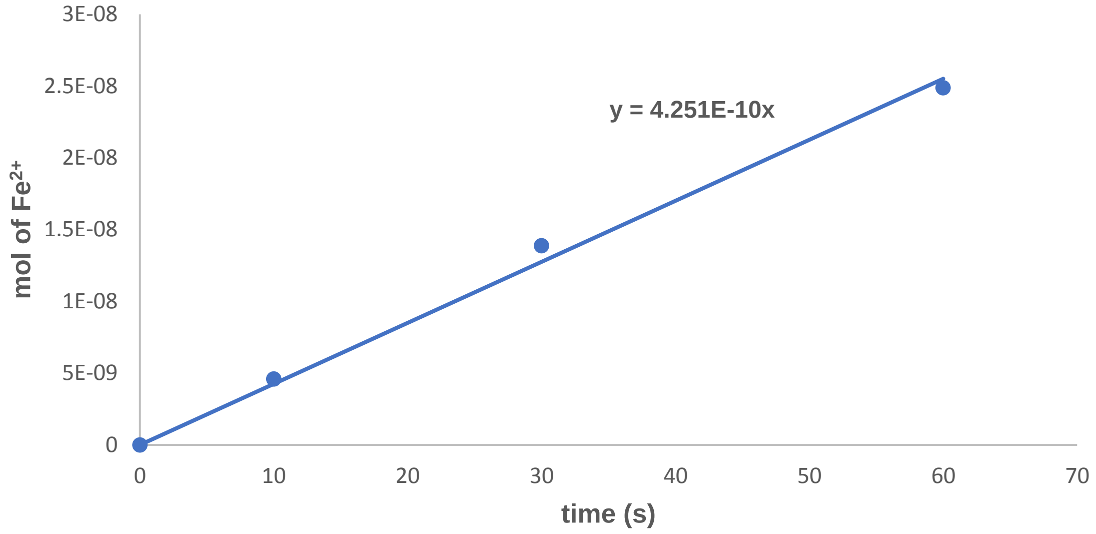

Fig. S7 – Moles of Fe^{2+} formed vs time of irradiation for determination of the photon flux.

The photon flux was then calculated using:

$$\text{photon flux} = \frac{\text{mol Fe}^{2+}}{\Phi \bullet t \bullet f}$$

Where Φ is the quantum yield of the ferrioxalate actinometer (1.0 at $\lambda = 450$ nm),[11a] t is the time, and f is the fraction of absorbed light at $\lambda = 450$ nm, where $f = 1 - 10^{-A}$. The absorbance (A) of the ferrioxalate solution at $\lambda = 450$ nm was measured by UV/Vis spectroscopy to be 1.887, therefore f = 0.9870.

$$\text{photon flux} = \frac{4.251 \times 10^{-10}}{1.0 \bullet 0.987} = 4.31 \times 10^{-10} \text{ einstein s}^{-1}$$

Determination of the Quantum Yield:

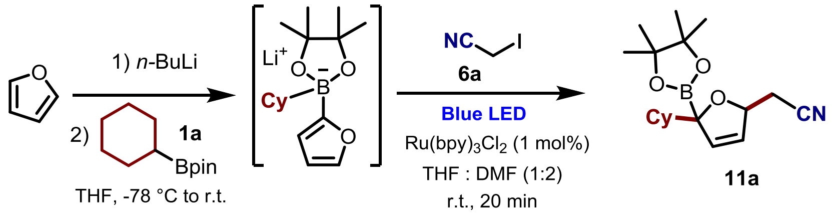

To a –78 °C stirred solution of furan (17.5 µL, 0.240 mmol, 1.20 equiv.) in THF (0.35 mL) under an N_2 atmosphere was added dropwise a 1.6 M solution of *n*-butyllithium in hexanes (145 µL, 0.230 mmol, 1.15 equiv.). The solution was left to warm to r.t. under vigorous stirring

over 30 minutes, after which it was cooled down to –78 °C. Boronic ester **1a** (45 μL, 0.20 mmol, 1.0 equiv.) was placed in a separate oven-dried test tube and dissolved in dry THF (0.3 mL) under a nitrogen atmosphere. The boronic ester solution was added dropwise to the cold reaction mixture under vigorous stirring. To ensure quantitative transfer of the material the test tube was washed twice with 0.2 mL of THF followed by dropwise transfer to the reaction mixture. The resulting solution was then left to warm to r.t. and stirred for 30 minutes. This provided a 0.158 M solution of boronate complex in THF of which 0.39 mL (0.061 mmol) was transferred to a N_2-purged quartz cuvette, equipped with a stir bar (5 × 3 mm) and sealed with a septum. To the cuvette was then added 0.61 mL of a degassed (15 minutes of N_2 sparging) solution of iodoacetonitrile (22 μL, 0.30 mmol, 1.5 equiv.) and $Ru(bpy)_3 \cdot 6H_2O$ (1.5 mg, 0.0020 mmol, 1.0 mol%) in DMF (2.0 mL). The reaction was irradiated for 20 min before addition of EtOAc (5 mL). The mixture was washed with H_2O (2 × 5 mL) and brine (5 mL) before concentration *in vacuo*. The yield was determined by ^{1}H NMR using dibromomethane as an internal standard to be 24%. The reaction was repeated a second time, giving a yield of 23%. Average yield = 23.5%.

The quantum yield (Φ) was then calculated using:

$$\Phi = \frac{\text{mol product}}{\text{photon flux} \bullet \text{t} \bullet \text{f}}$$

Where t is the time (1200 s) and f is the fraction of light absorbed by the $Ru(bpy)_3Cl_2$ catalyst at λ = 450 nm (for a 6.7 x 10^{-4} M solution in DMF, this was determined by UV/Vis spectroscopy to be 0.998).

$$\Phi = \frac{1.43 \times 10^{-5}}{4.31 \times 10^{-10} \bullet 1200 \bullet 0.998} = 27.8$$

The quantum yield of the process is well above unity, suggesting a radical chain propagation to dominate the reaction mechanism.

3.5 Control Experiments for Indole Three Component Couplings

Procedure for the reaction carried out in the presence of 1,1-diphenylethylene

To a 0 °C stirred solution of *N*-methylindole (30 µL, 0.24 mmol, 1.2 equiv.) in THF (0.35 mL) under N_2 atmosphere was added dropwise a 1.7 M solution of *tert*-butyllithium in pentane (150 µL, 0.270 mmol, 1.35 equiv.). The solution was left to warm to r.t. and stirred for 60 minutes, resulting in the formation of a white precipitate. The reaction mixture was then cooled down to -78 °C. Cyclohexylboronic acid pinacol ester (45 µL, 0.20 mmol) was placed in a separate oven-dried test tube and dissolved in dry THF (0.3 mL) under a nitrogen atmosphere. The boronic ester solution was added dropwise to the cold reaction mixture under vigorous stirring. To ensure quantitative transfer of the material the test tube was washed twice with 0.2 mL of THF followed by dropwise transfer to the reaction mixture. The reaction was then left to warm at r.t. and stirred for 30 minutes, resulting in a clear solution. Afterwards, a solution of the iodoacetonitrile (22 µL, 0.30 mmol, 1.5 equiv.) and 1,1-diphenylthylene (35 µL, 0.20 mmol, 1.0 equiv.) in DMF (2.0 mL) was added quickly by syringe, and the mixture was stirred vigorously for 16 h. Oxidation/workup procedure **A** was then carried out (reaction time: 60 minutes). Dibromomethane (14 µL, 0.20 mmol) was added to the crude residue and the mixture was analysed by NMR. The three-component alkylation product **14a** was detected in 76% NMR yield.

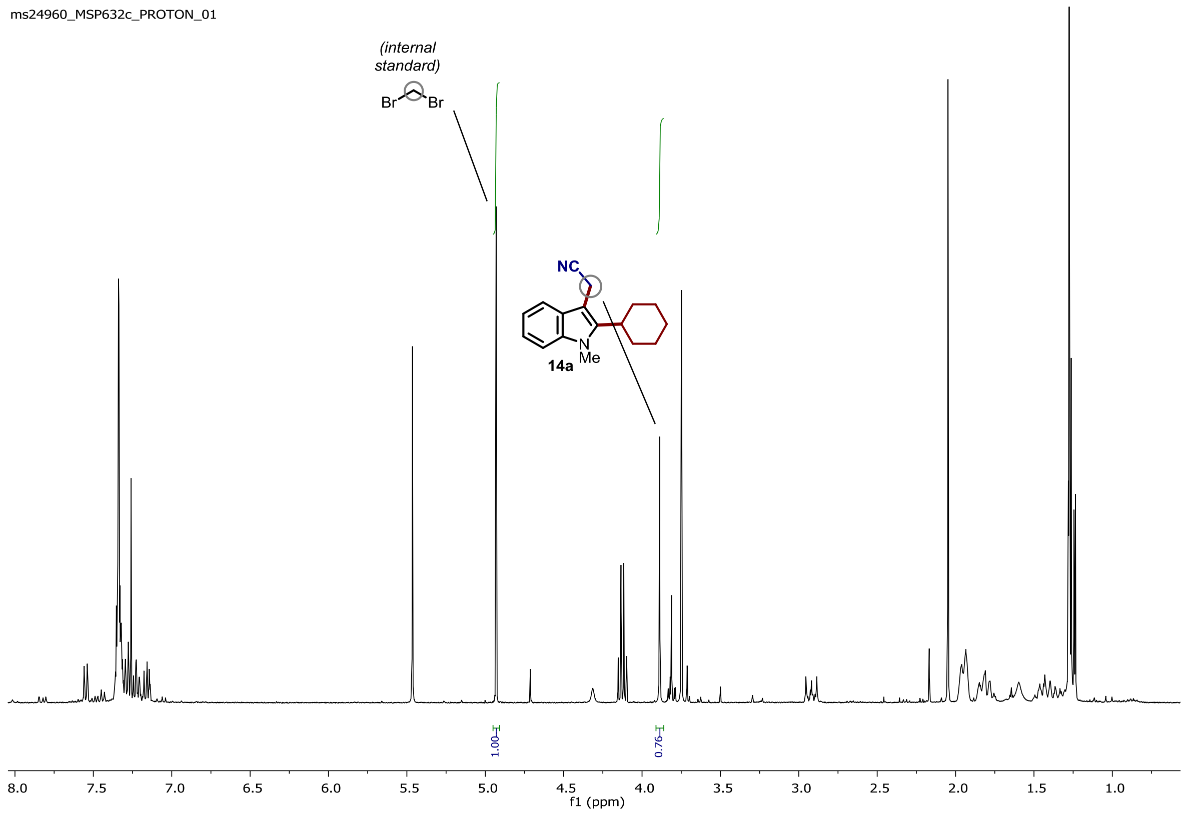

Fig. S8 – ^{1}H-NMR analysis of the crude mixture of the reaction carried out in the presence of 1 equiv. of 1,1-diphenylethylene as radical inhibitor.

Procedure for the control reaction between N-methylindole and iodoacetonitrile.

To a r.t. stirred solution of *N*-methylindole (25 µL, 0.20 mmol, 1.0 equiv.) in THF (0.35 mL) under N_2 atmosphere was quickly added a solution of the iodoacetonitrile (22 µL, 0.30 mmol, 1.5 equiv.) in DMF (2.0 mL) through a syringe, and the mixture was stirred vigorously for 16 h. The reaction mixture was then diluted with EtOAc (150 mL) and washed with a 20% solution of sodium thiosulfate (50 ml), twice with water (100 mL + 4 mL brine to induce phase separation), and finally with brine (100 mL). The resulting organic phase was dried ($MgSO_4$), filtered and concentrated *in vacuo*. Dibromomethane (14 µL, 0.2 mmol) was added to the crude residue and the mixture was analysed through NMR. The recovery of the starting material amounted to 88%, with no other indole derivatives detected in the mixtures, thereby excluding the formation of Friedel-Crafts products.

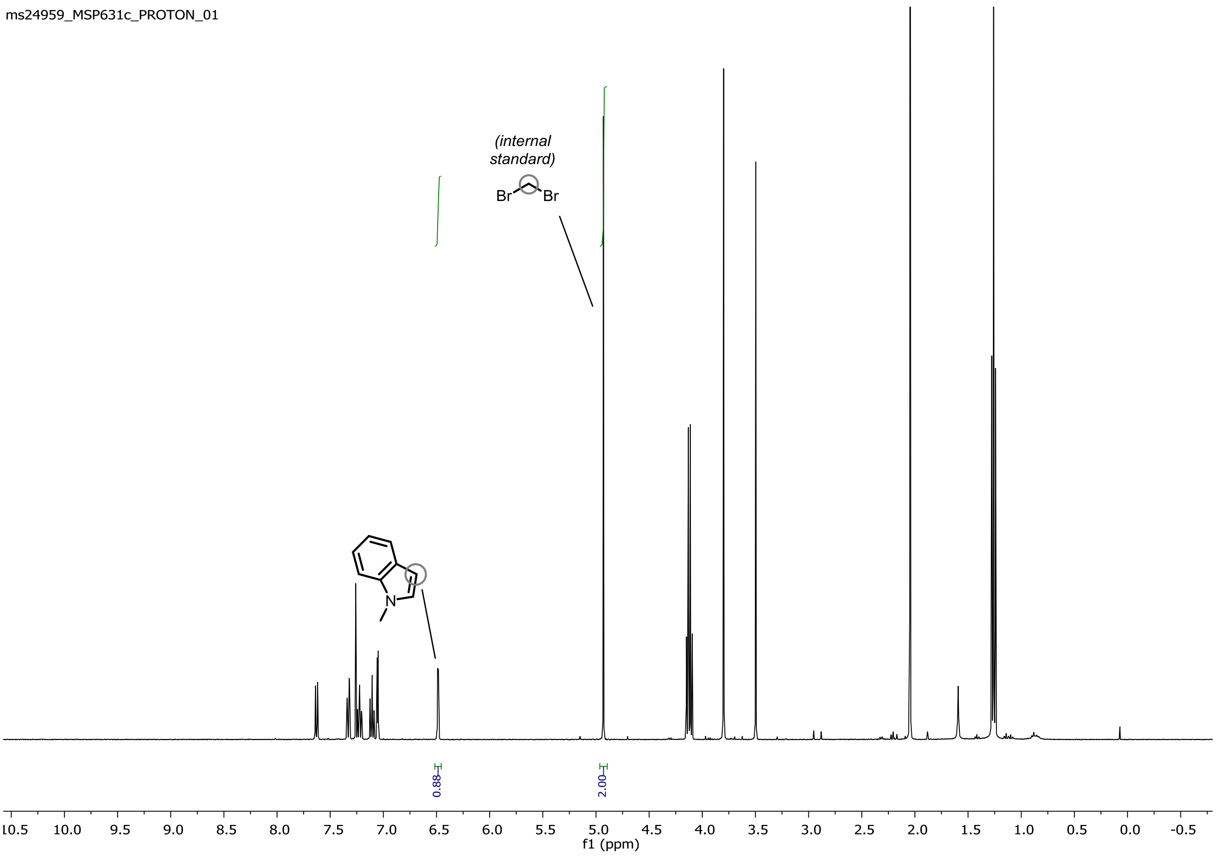

Fig. S9 – ^{1}H-NMR analysis of the crude mixture of the attempted reaction between N-Me indole and iodoacetonitrile.

4. References

[1] P. Klahn, H. Erhardt, A. Kotthaus, S. F. Kirsch *Angew. Chem. Int. Ed.* **2014**, *53*, 7913–7917.

[2] P. R. Olivato, S. Guerrero, R. Rittner *Magn. Res. Chem.* **1987**, *25*, 179-186.

[3] G. Filippini, M. Silvi, P. Melchiorre *Angew. Chem. Int. Ed.* **2017**, *56*, 4447.

[4] M. J. Hesse, C. P. Butts, C. L. Willis, V. K. Aggarwal *Angew. Chem. Int. Ed.* **2012**, *51*, 12444.

[5] D. Noh, H. Chea, J. Ju, J. Yun *Angew. Chem. Int. Ed.* **2009**, *48*, 6062.

[6] H. Ito, K. Kubota, *Org. Lett.* **2012**, *14*, 890–893.

[7] C. Sandford, R. Rasappan, V. K. Aggarwal *J. Am. Chem. Soc.* **2015**, *137*, 10100–10103.

[8] J. V. Caspar, T. J. Meyer *J. Am. Chem. Soc.* **1983**, *105*, 5583.

[9] J. R. Lakowicz, Quenching of Fluorescence in *Principles of Fluorescence Spectroscopy*; Plenum Press: New York, 1983; pp. 258-301.

[10] H. G. Roth, N. A. Romero, D. A. Nicewicz, *Synlett* **2016**, *27*, 714–723.

[11] a) M. Montalti, A. Credi, L. Prodi, M. T. Gandolfi, *Handbook of Photochemistry*, 3rd ed.; CRC/Taylor & Francis: Boca Raton, FL, 2006; b) M. A. Cismesia, T. P. Yoon, *Chem. Sci.* **2015**, *6*, 5426.

5. HPLC Chromatograms

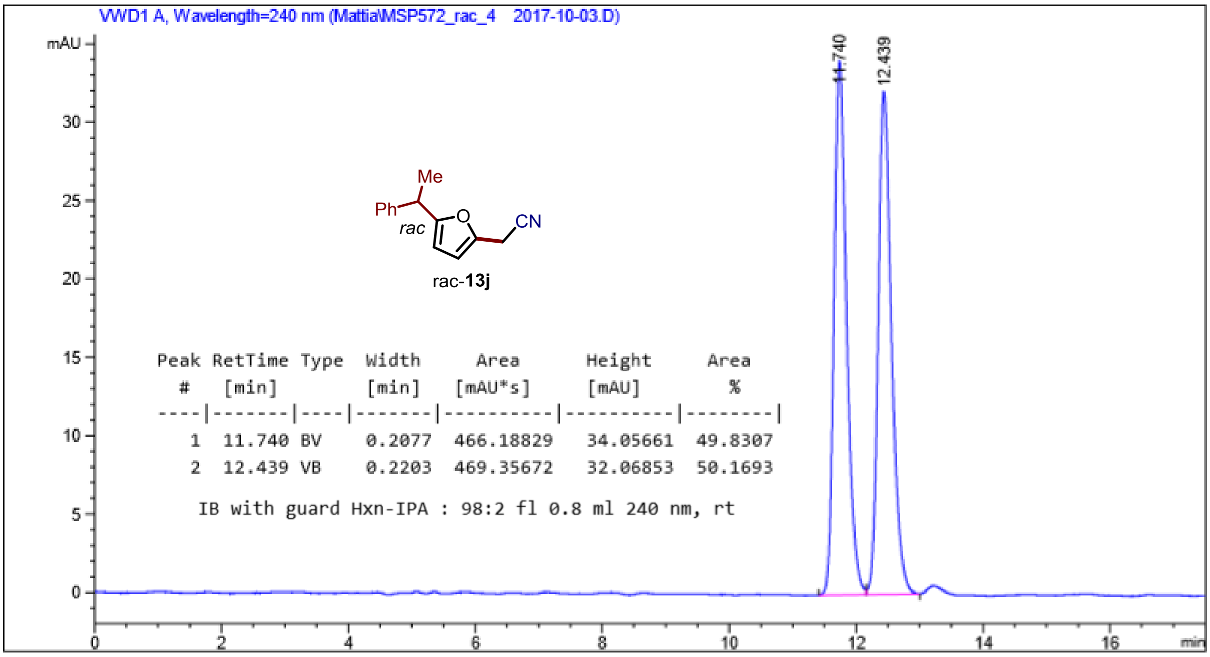

Peak #	RetTime [min]	Type	Width [min]	Area [mAU*s]	Height [mAU]	Area %
1	11.740	BV	0.2077	466.18829	34.05661	49.8307
2	12.439	VB	0.2203	469.35672	32.06853	50.1693

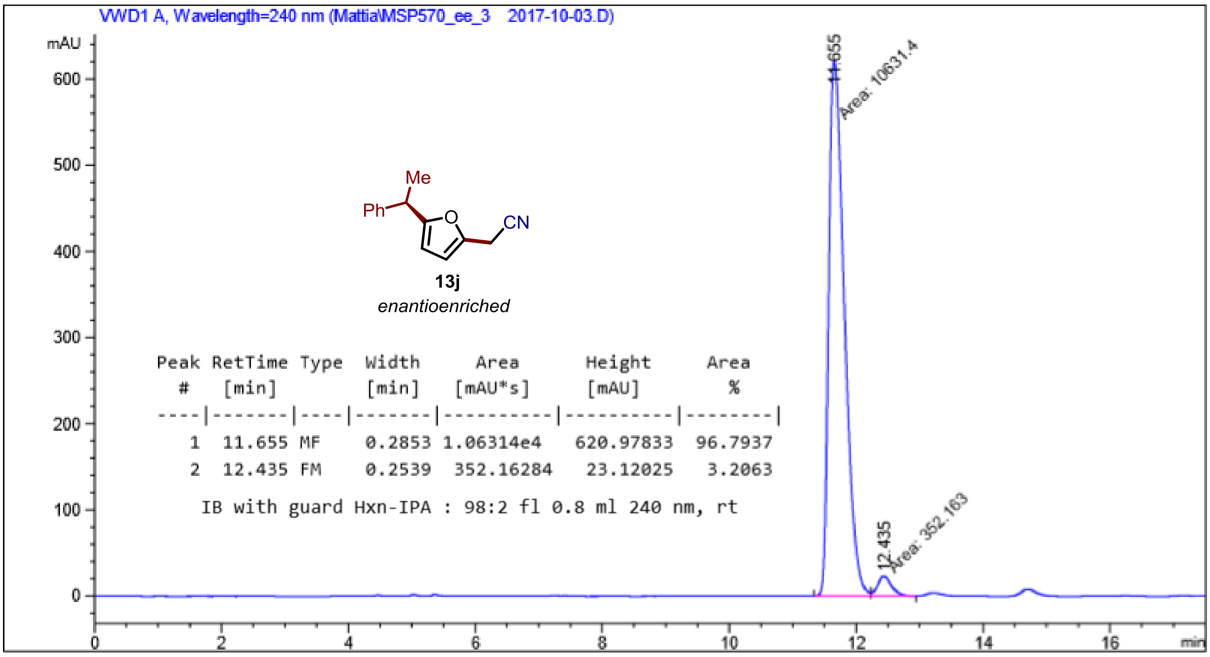

Peak #	RetTime [min]	Type	Width [min]	Area [mAU*s]	Height [mAU]	Area %
1	11.655	MF	0.2853	1.06314e4	620.97833	96.7937
2	12.435	FM	0.2539	352.16284	23.12025	3.2063

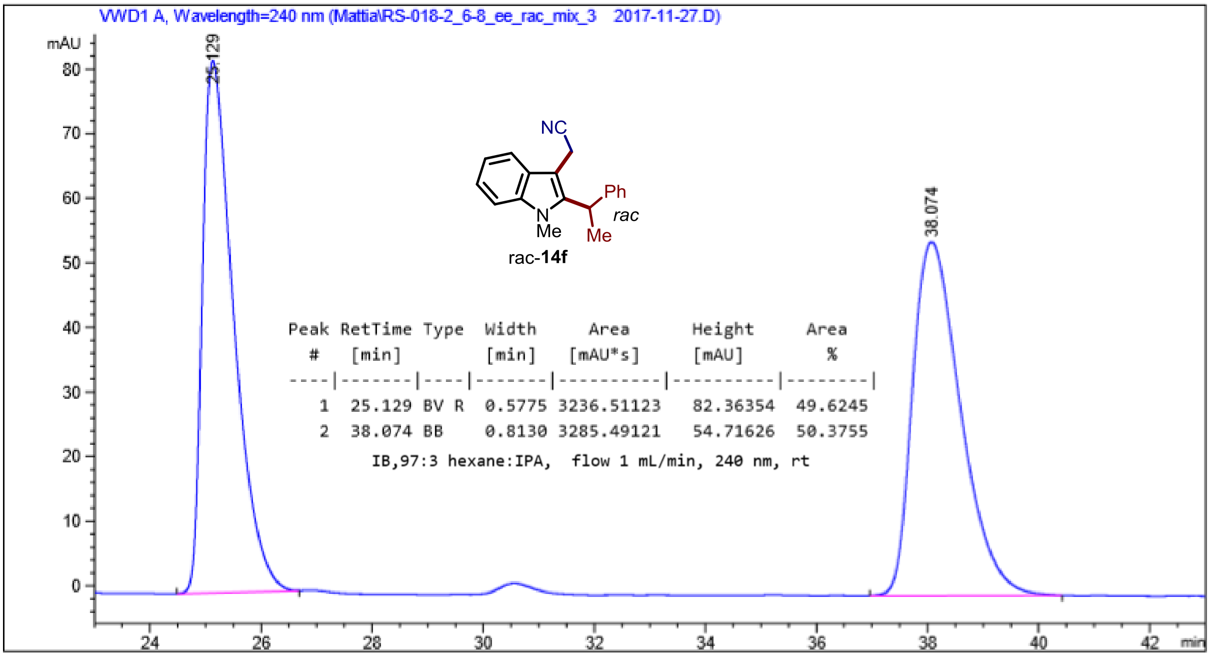

Peak #	RetTime [min]	Type	Width [min]	Area [mAU*s]	Height [mAU]	Area %
1	25.129	BV R	0.5775	3236.51123	82.36354	49.6245
2	38.074	BB	0.8130	3285.49121	54.71626	50.3755

IB,97:3 hexane:IPA, flow 1 mL/min, 240 nm, rt

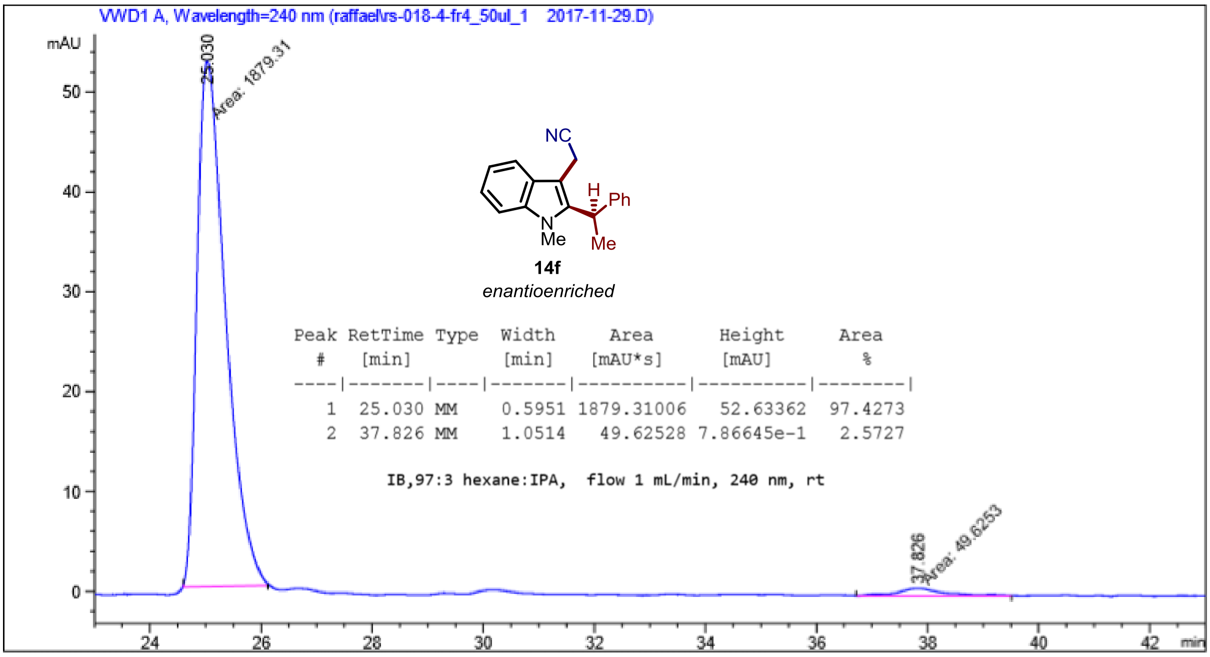

Peak #	RetTime [min]	Type	Width [min]	Area [mAU*s]	Height [mAU]	Area %
1	25.030	MM	0.5951	1879.31006	52.63362	97.4273
2	37.826	MM	1.0514	49.62528	7.86645e-1	2.5727

IB,97:3 hexane:IPA, flow 1 mL/min, 240 nm, rt

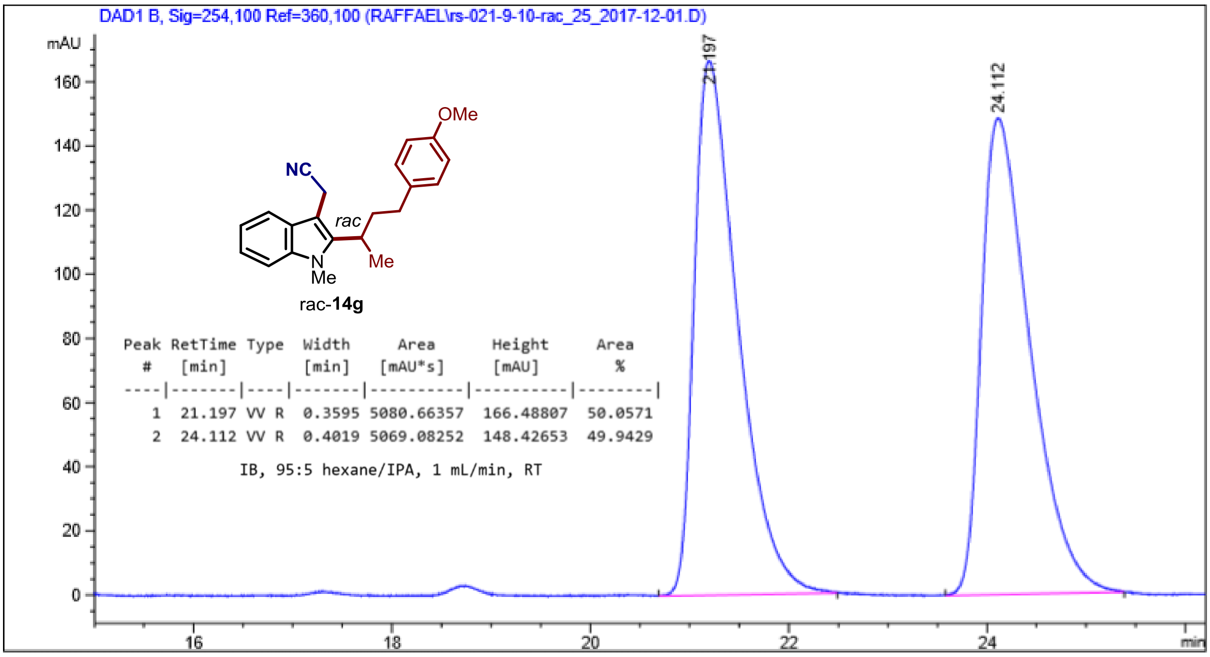

Peak #	RetTime [min]	Type	Width [min]	Area [mAU*s]	Height [mAU]	Area %
1	21.197	VV R	0.3595	5080.66357	166.48807	50.0571
2	24.112	VV R	0.4019	5069.08252	148.42653	49.9429

IB, 95:5 hexane/IPA, 1 mL/min, RT

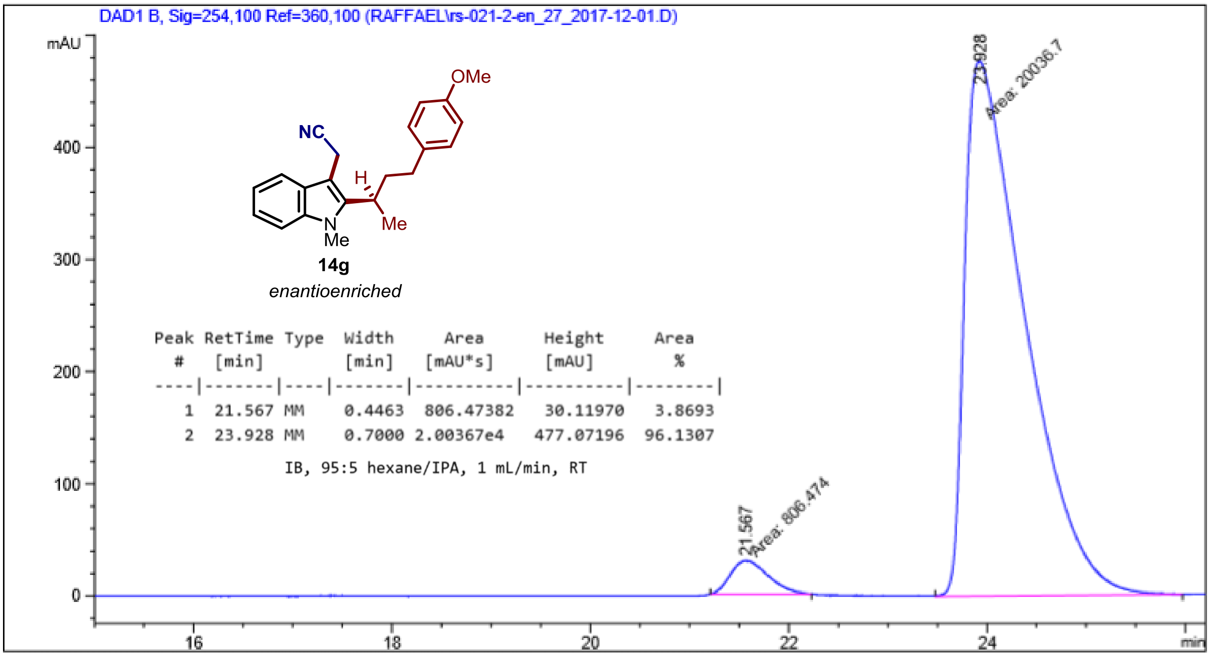

Peak #	RetTime [min]	Type	Width [min]	Area [mAU*s]	Height [mAU]	Area %
1	21.567	MM	0.4463	806.47382	30.11970	3.8693
2	23.928	MM	0.7000	2.00367e4	477.07196	96.1307

IB, 95:5 hexane/IPA, 1 mL/min, RT

6. NMR Spectra

^{1}H NMR of compound **13a** (500 MHz, $CDCl_3$)

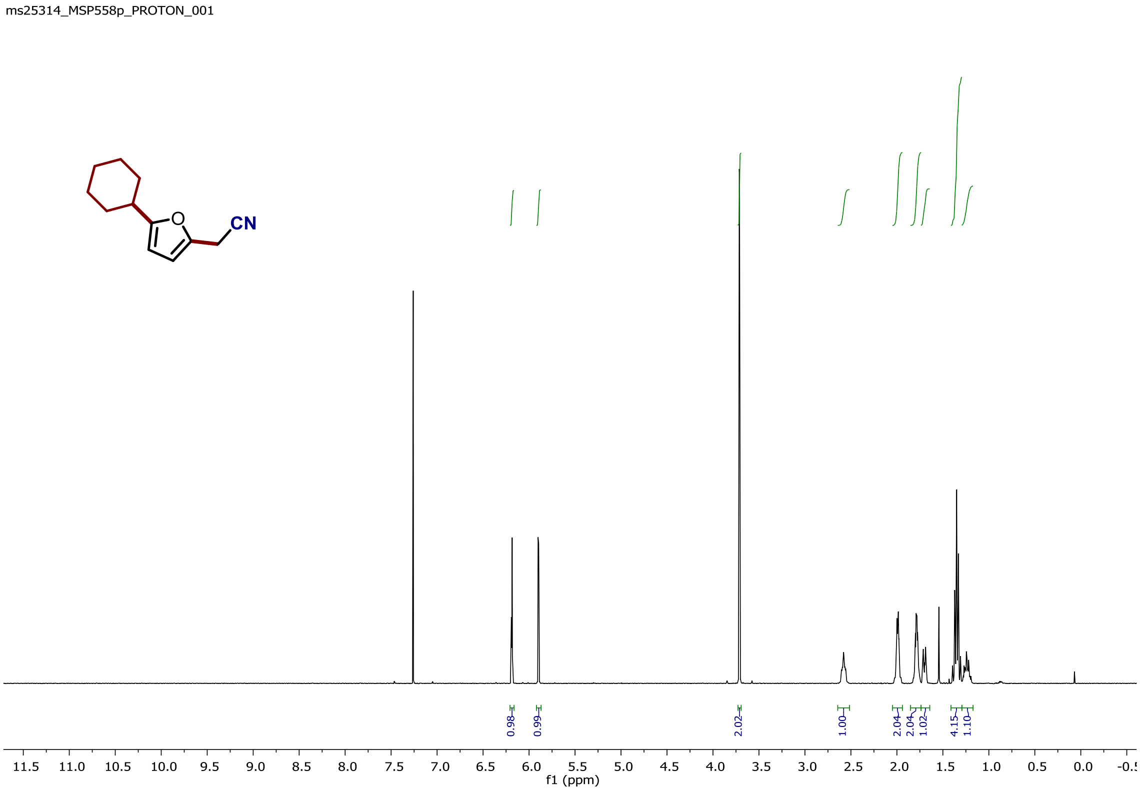

^{13}C NMR of compound **13a** (125 MHz, $CDCl_3$)

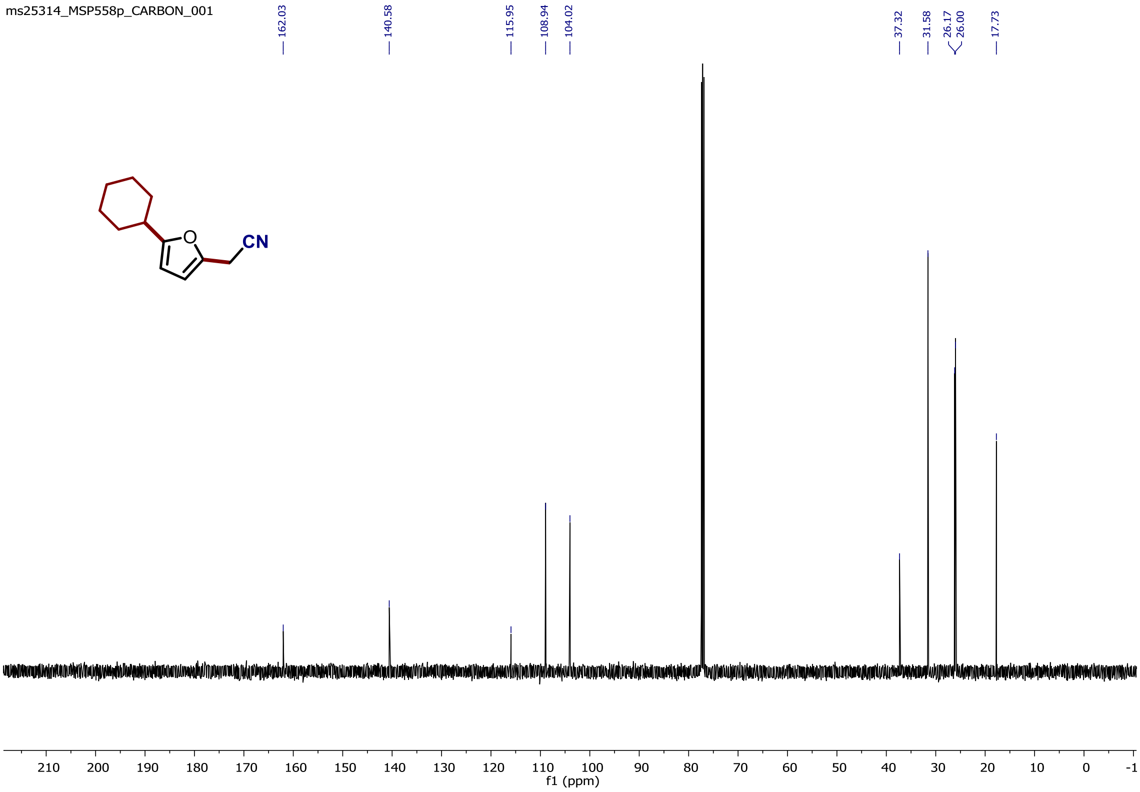

^{1}H NMR of compound **13b** (400 MHz, $CDCl_3$)

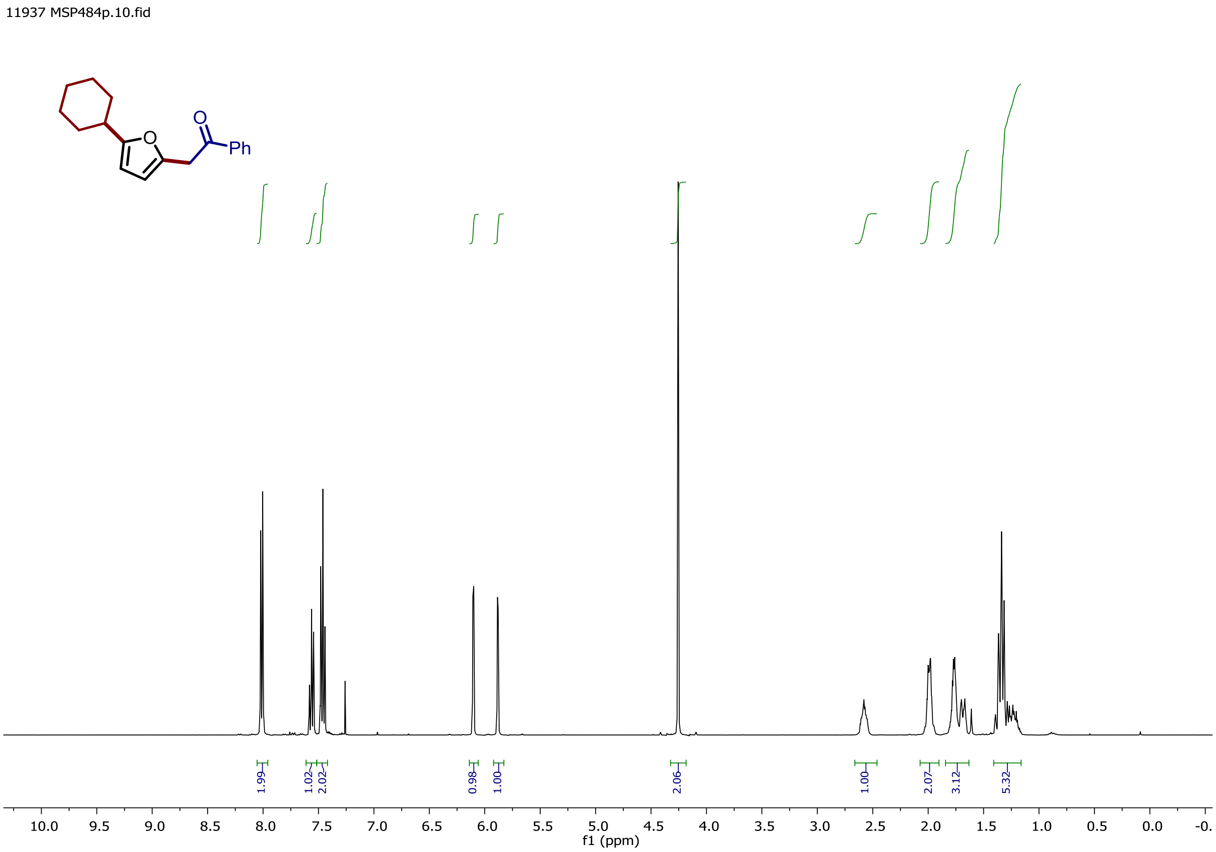

^{13}C NMR of compound **13b** (100 MHz, $CDCl_3$)

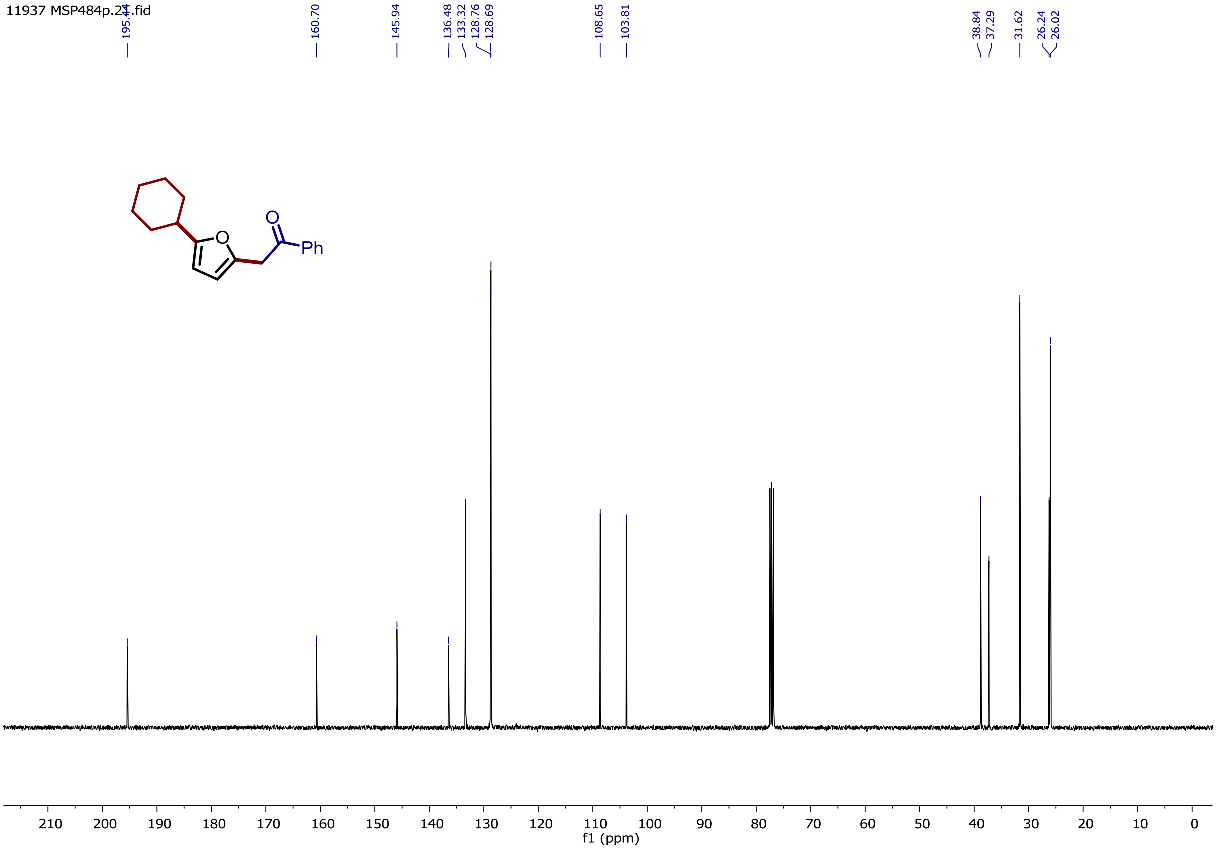

^{1}H NMR of compound **13c** (500 MHz, $CDCl_3$)

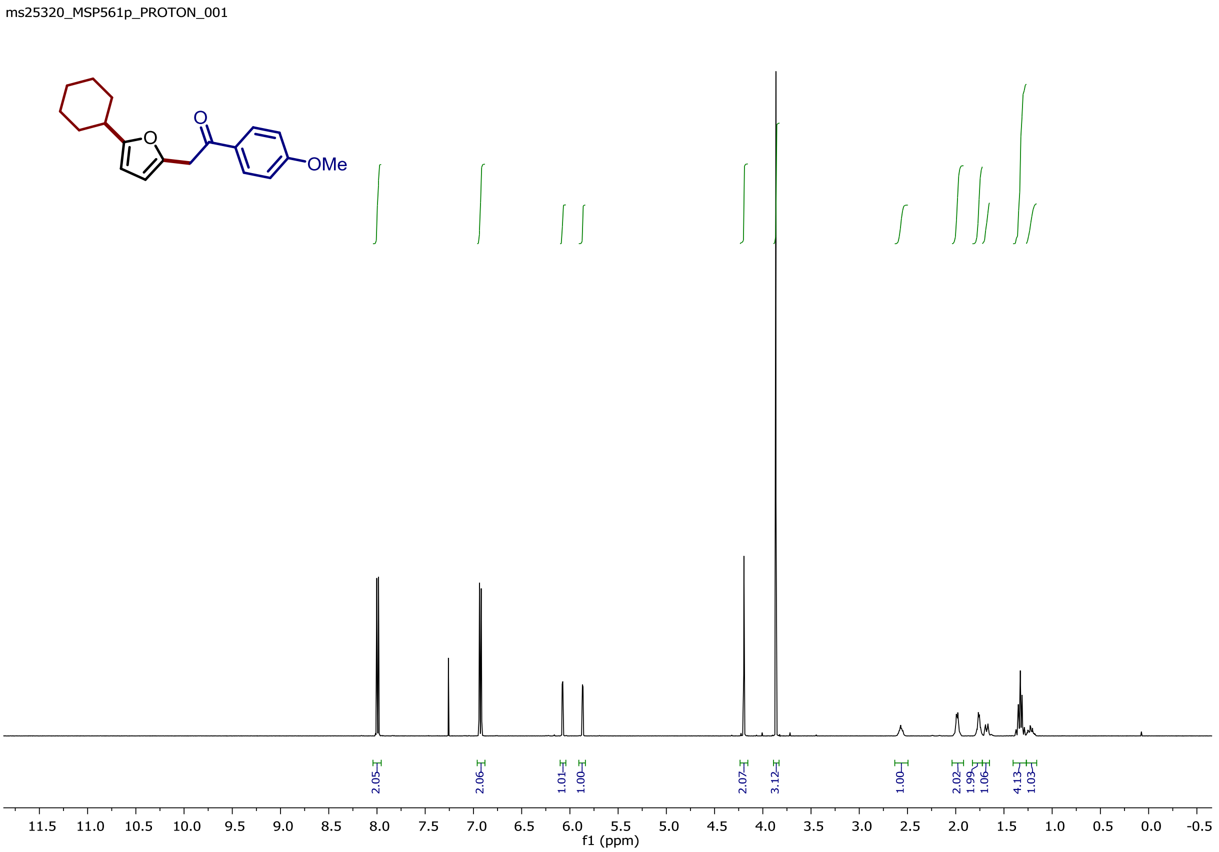

^{13}C NMR of compound **13c** (125 MHz, $CDCl_3$)

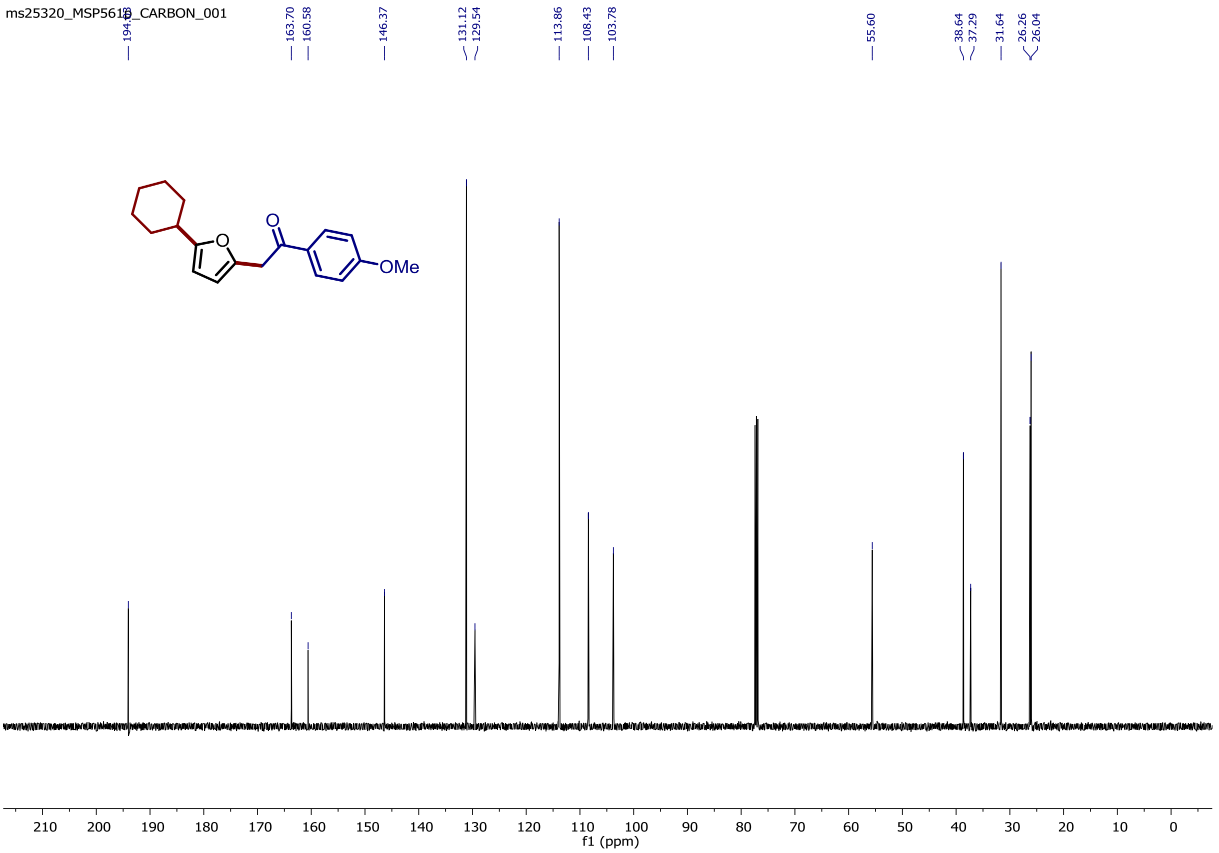

^{1}H NMR of compound **13d** (500 MHz, $CDCl_3$)

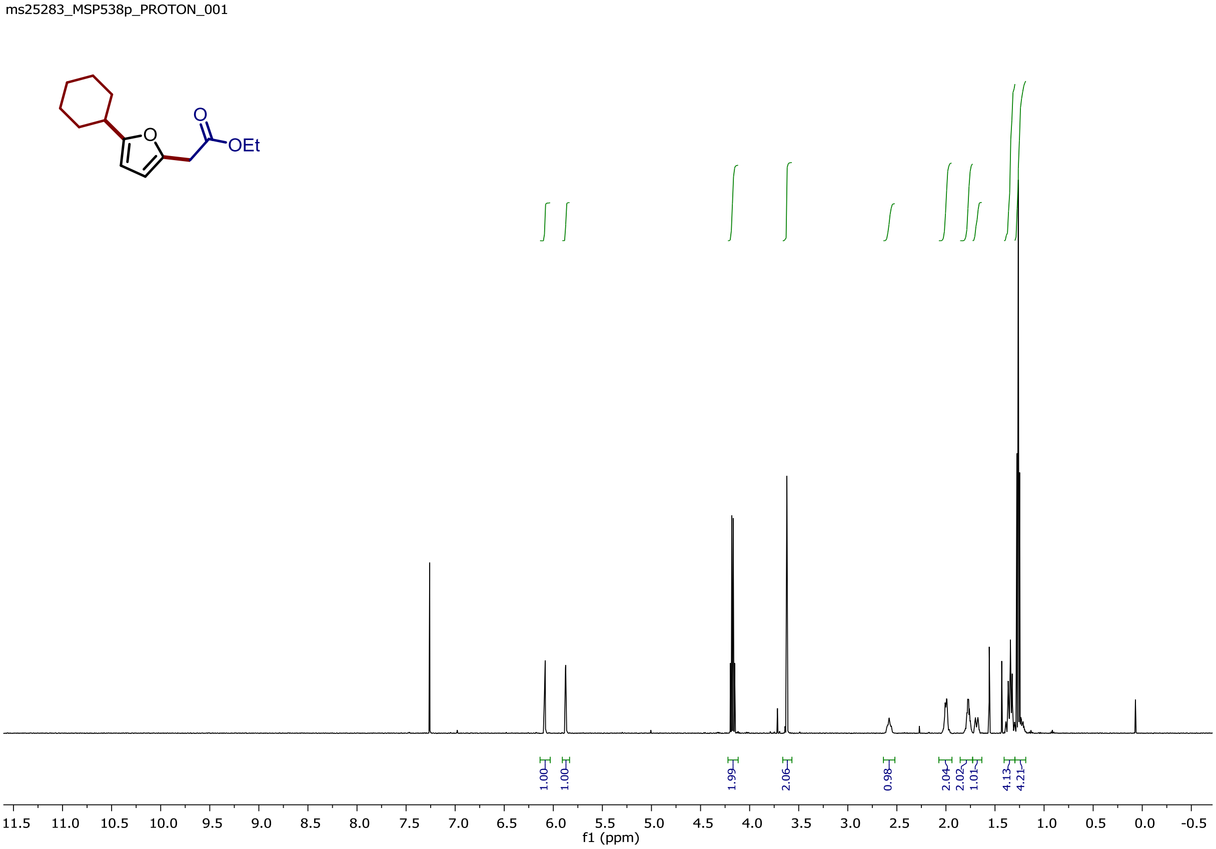

^{13}C NMR of compound **13d** (125 MHz, $CDCl_3$)

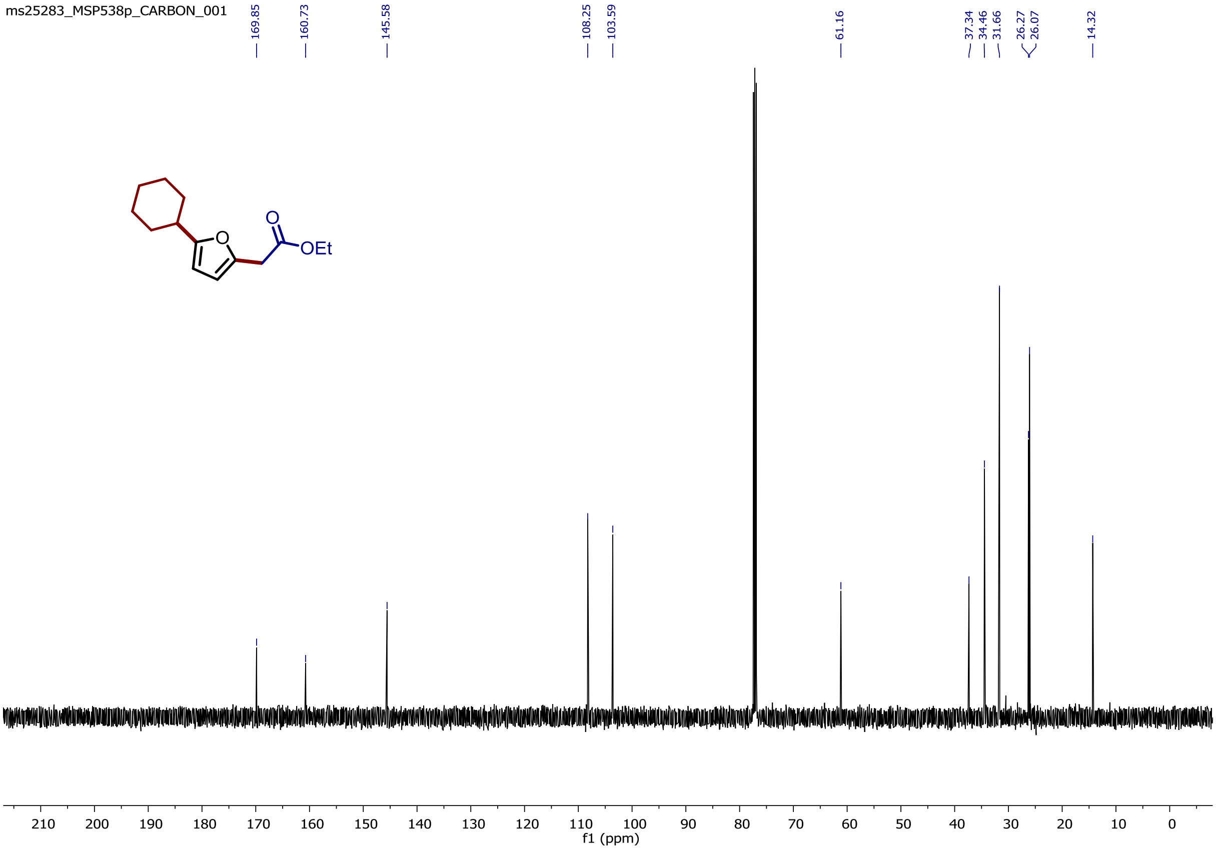

^{1}H NMR of compound **13e** (500 MHz, $CDCl_3$)

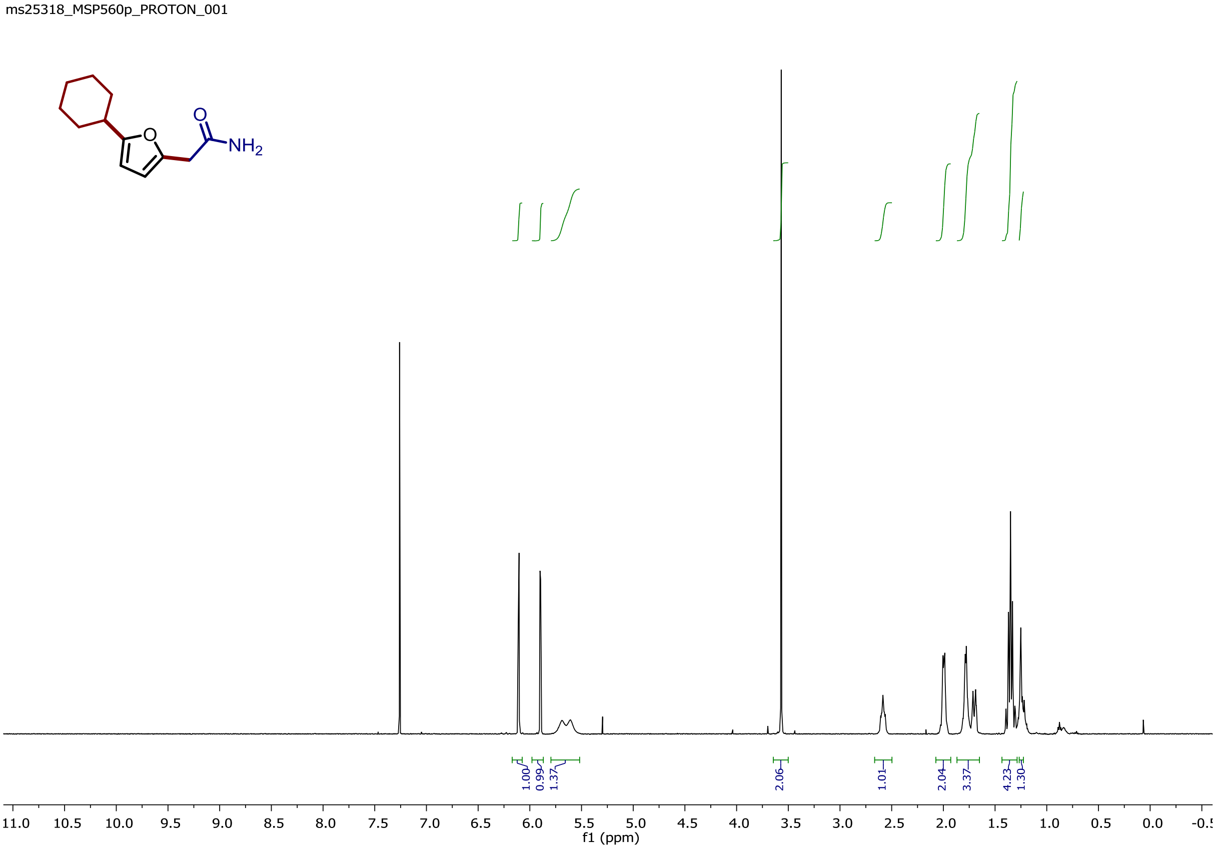

^{13}C NMR of compound **13e** (125 MHz, $CDCl_3$)

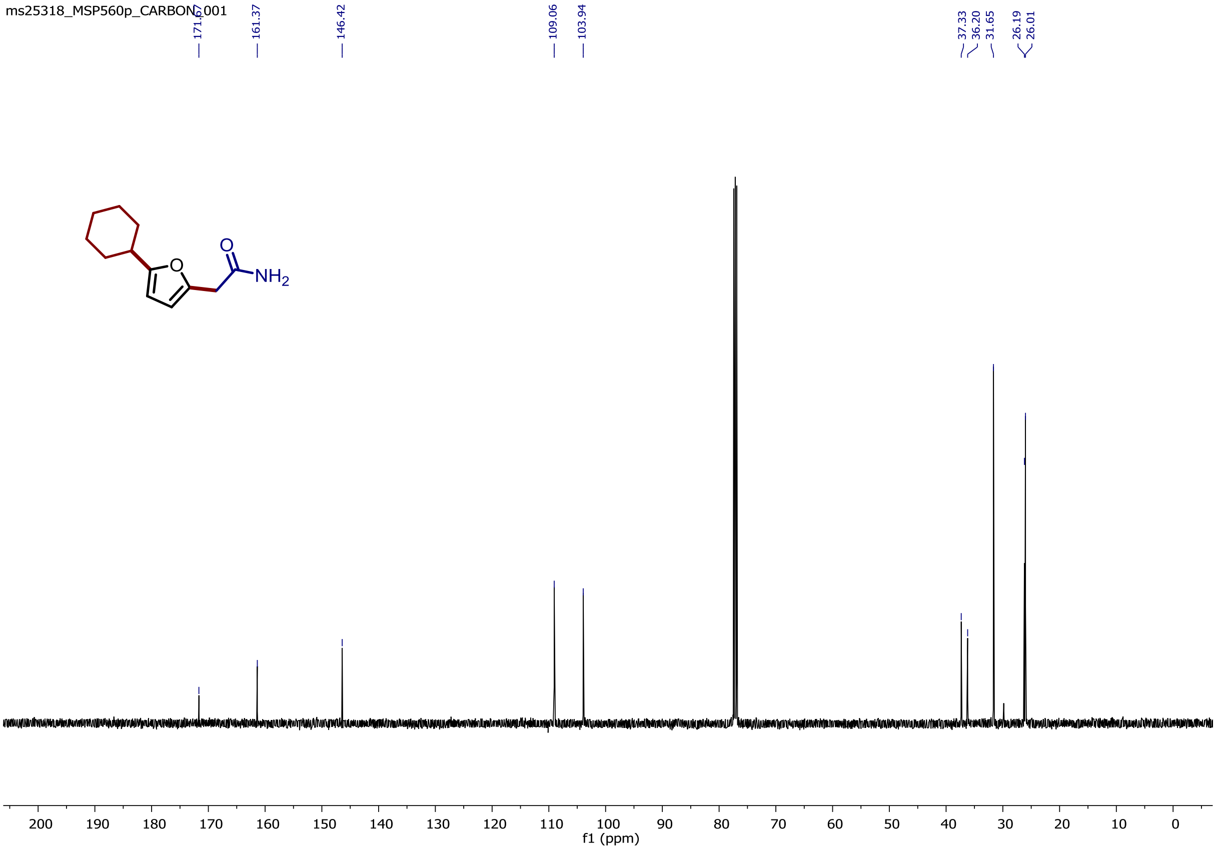

^{1}H NMR of compound **13f** (500 MHz, $CDCl_3$)

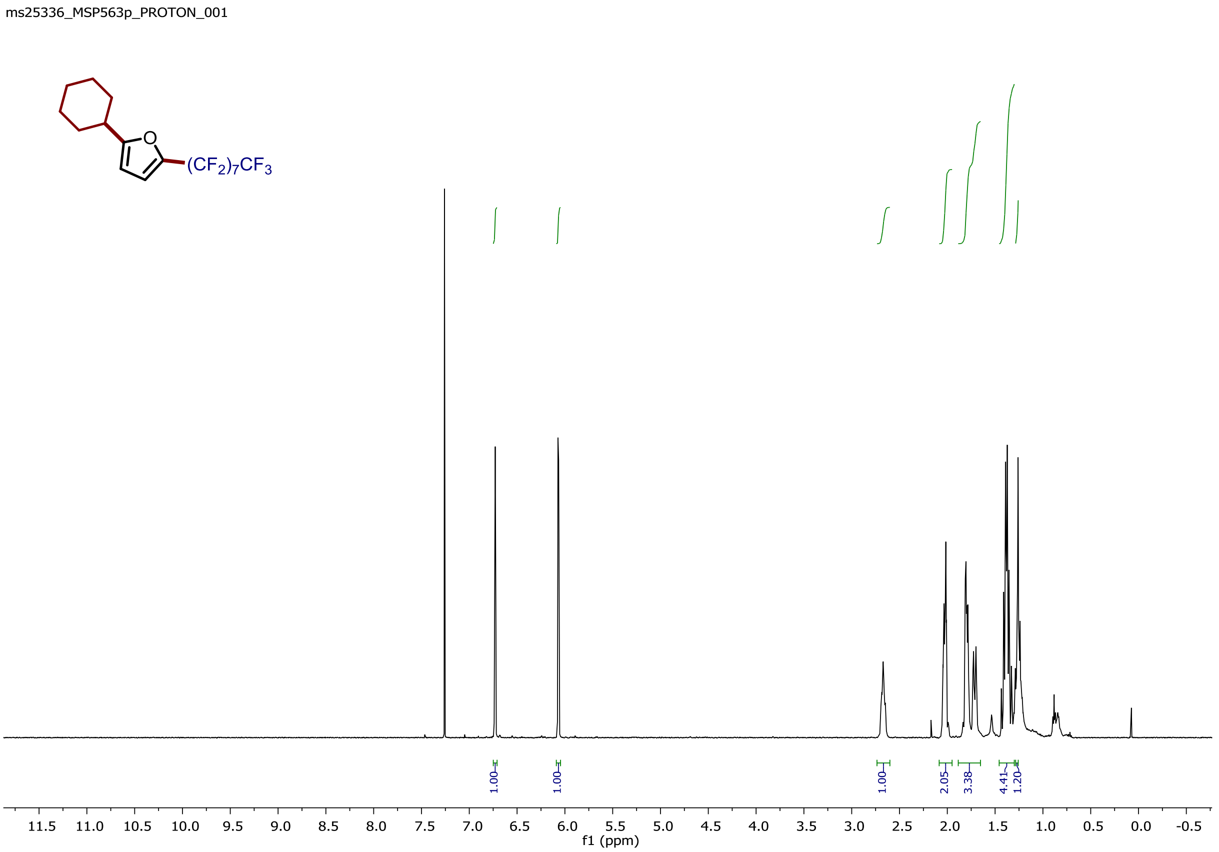

^{13}C NMR of compound **13f** (125 MHz, $CDCl_3$)

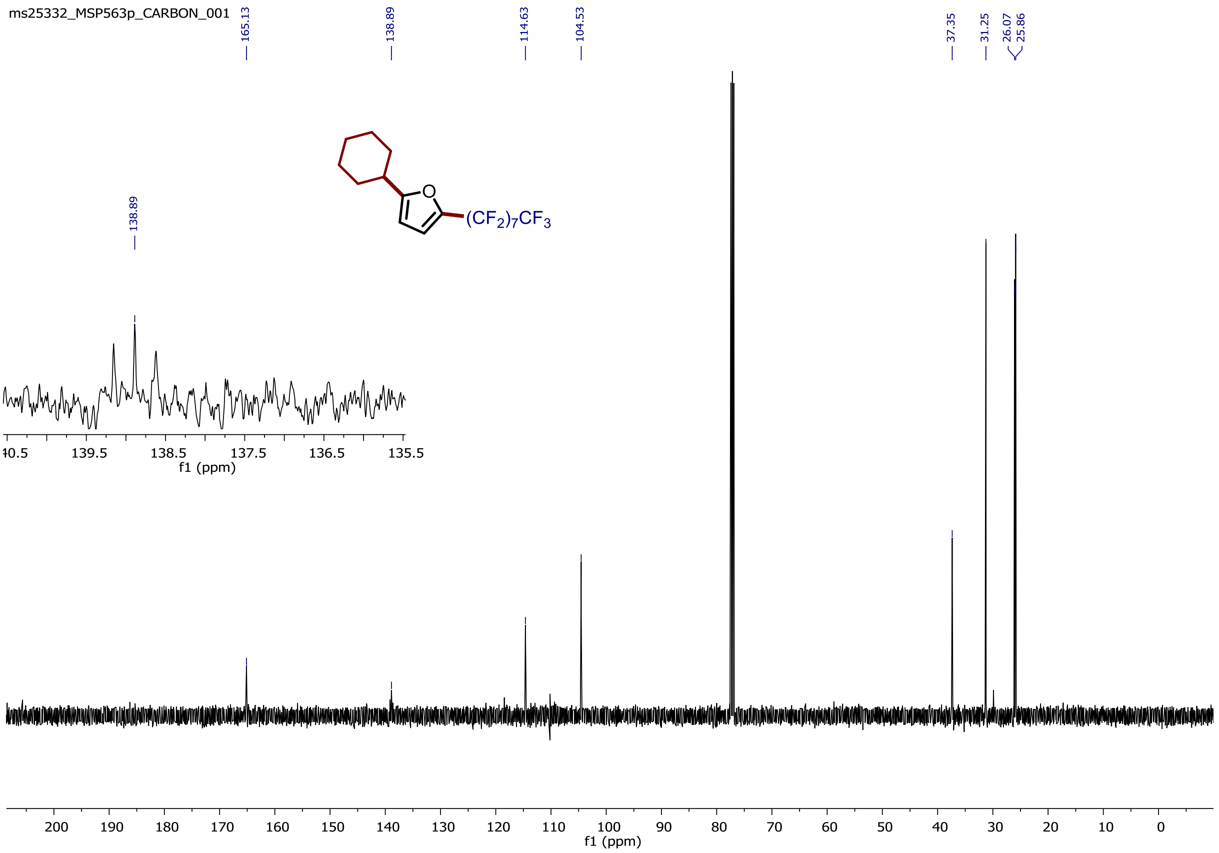

^{19}F NMR of compound **13f** (470 MHz, CDCl$_3$)

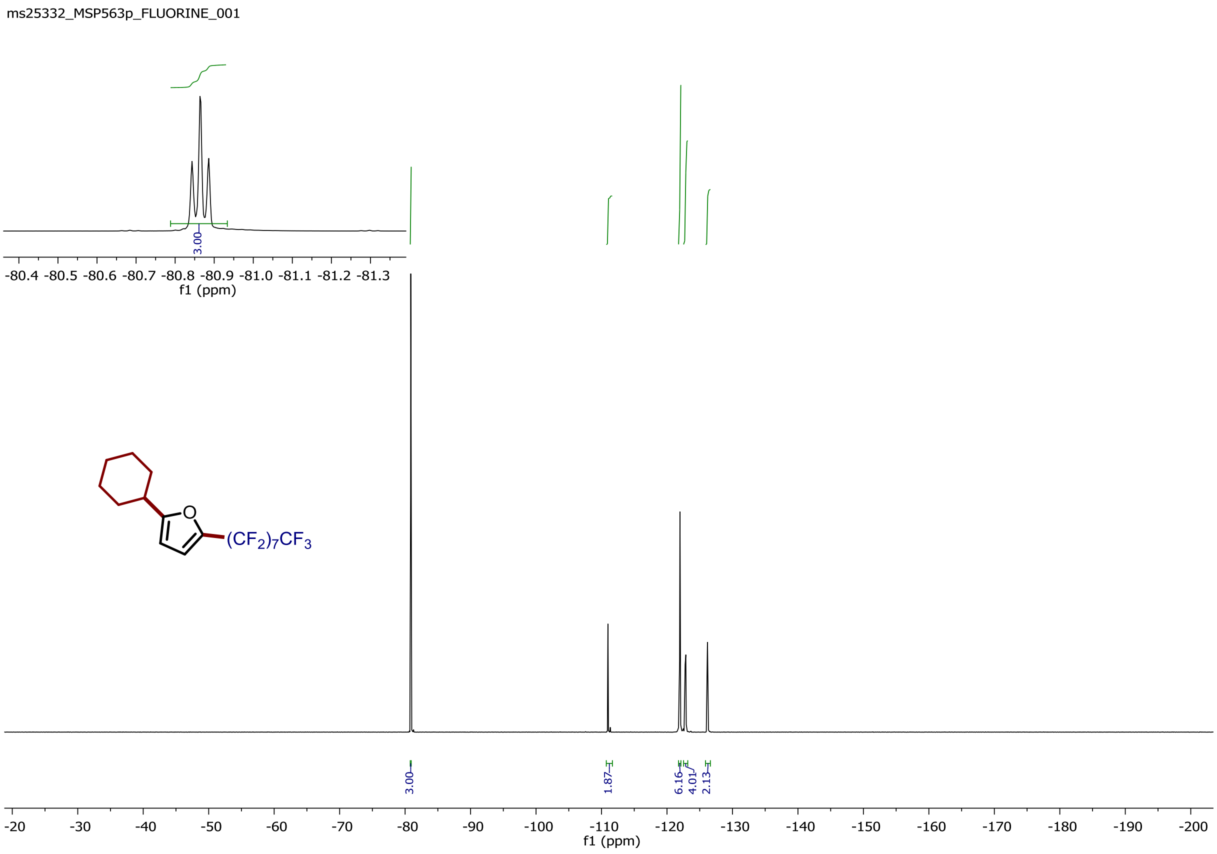

^{1}H NMR of compound **13g** (500 MHz, $CDCl_3$)

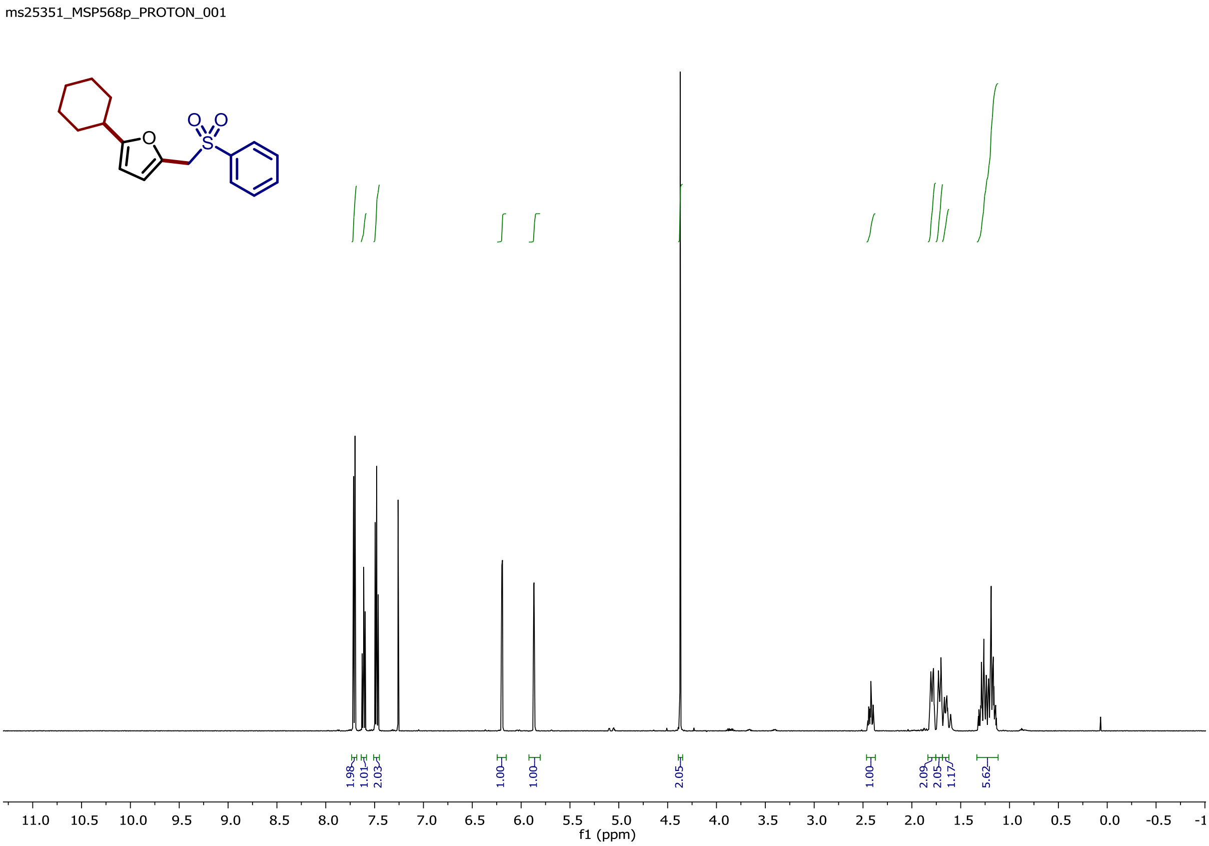

^{13}C NMR of compound **13g** (125 MHz, $CDCl_3$)

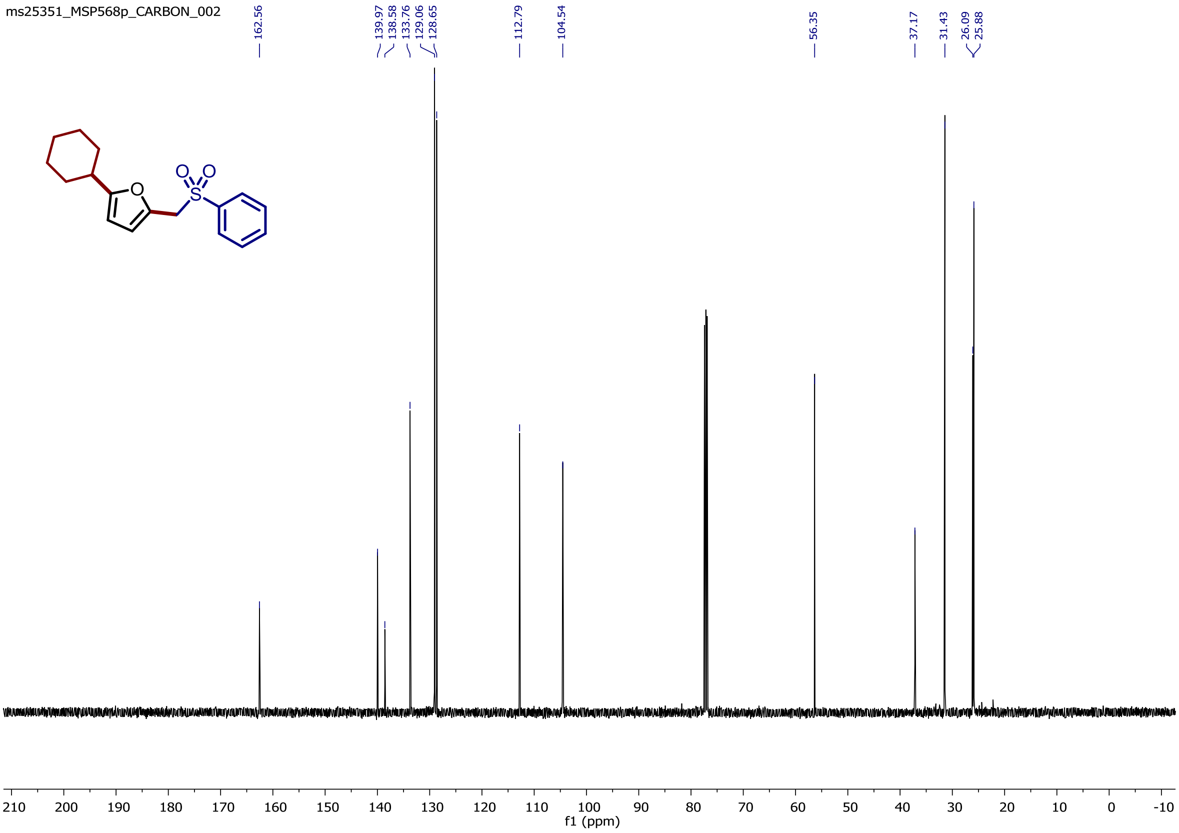

^{1}H NMR of compound **13h** (400 MHz, $CDCl_3$)

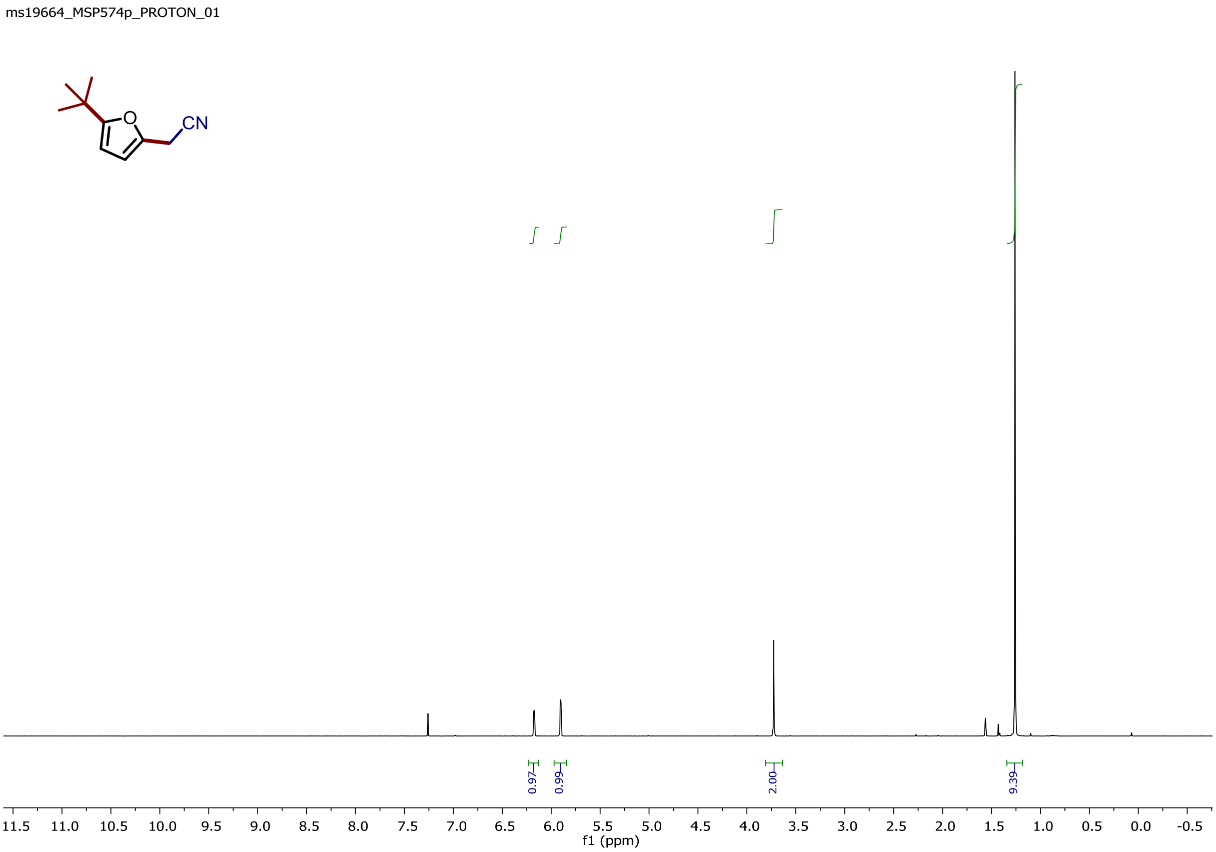

^{13}C NMR of compound **13h** (100 MHz, $CDCl_3$)

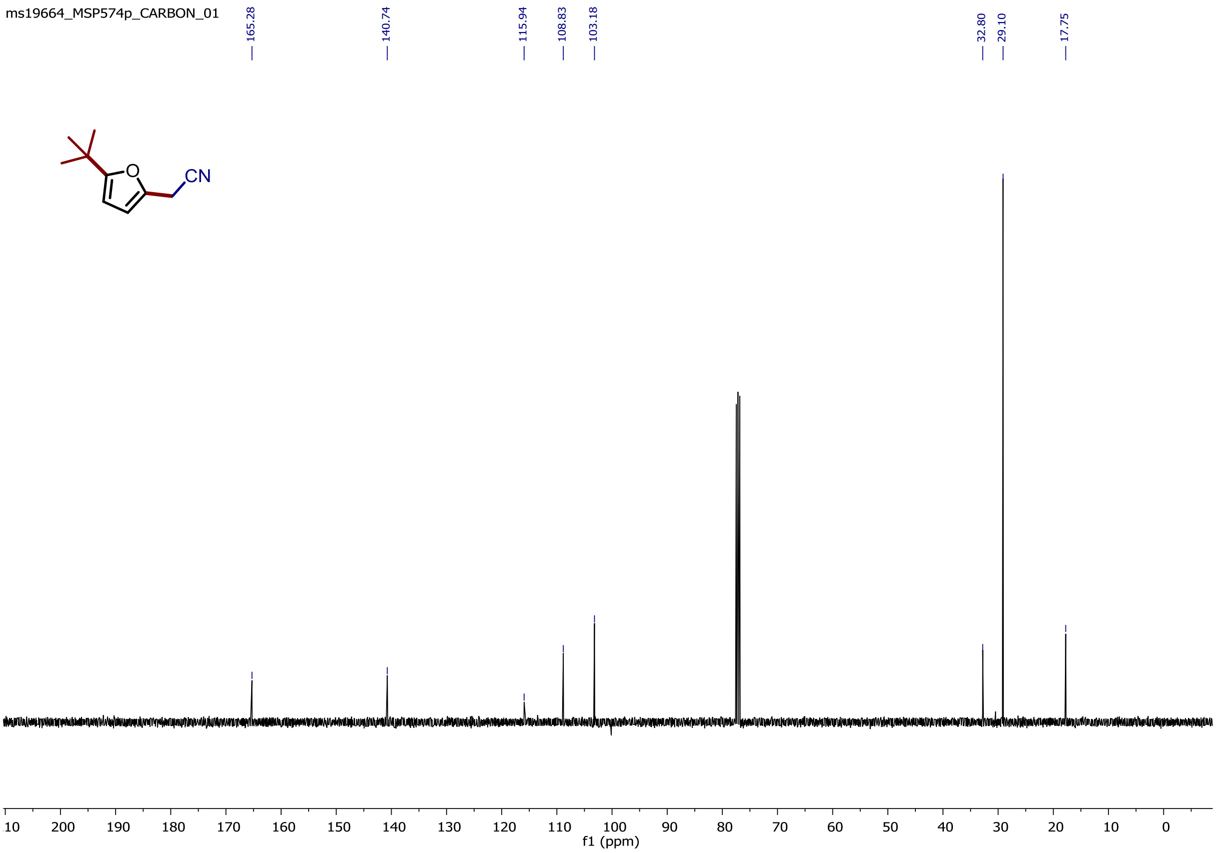

^{1}H NMR of compound **13i** (500 MHz, $CDCl_3$)

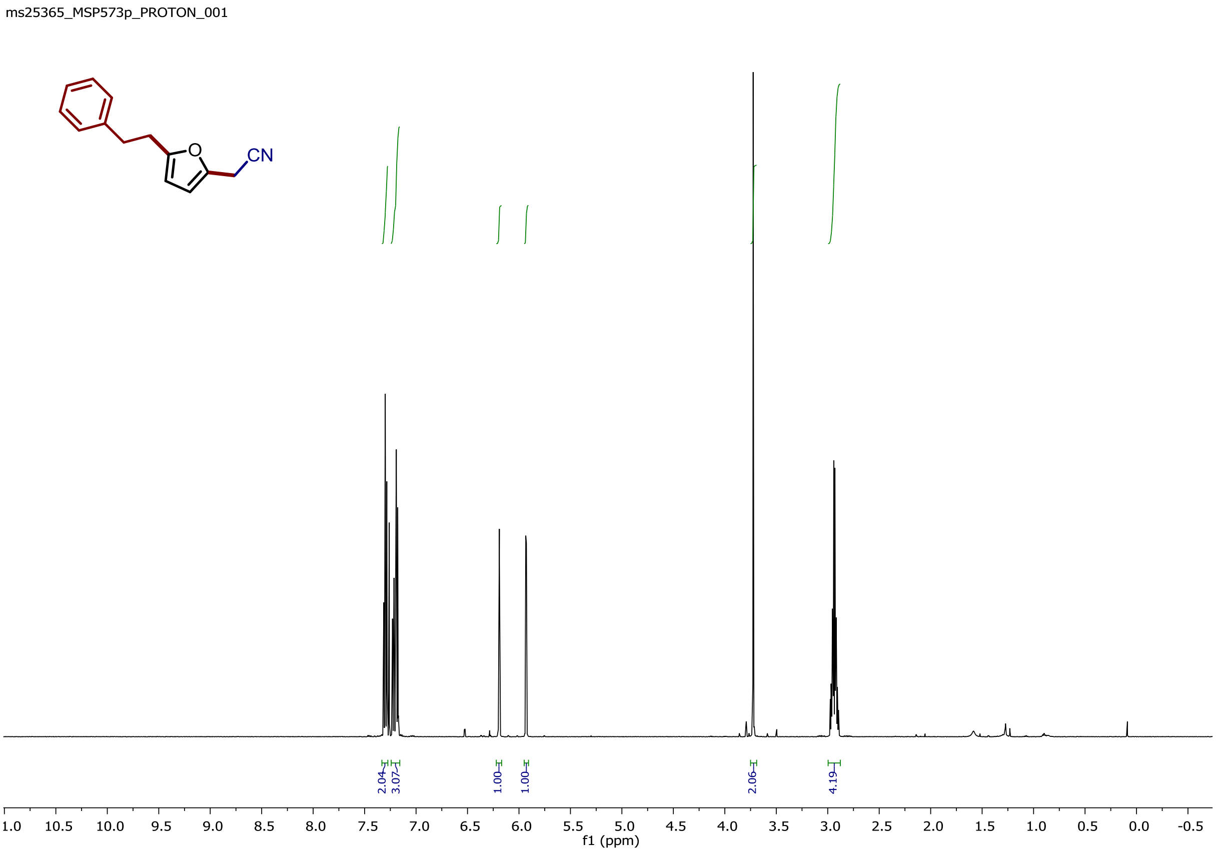

^{13}C NMR of compound **13i** (125 MHz, $CDCl_3$)

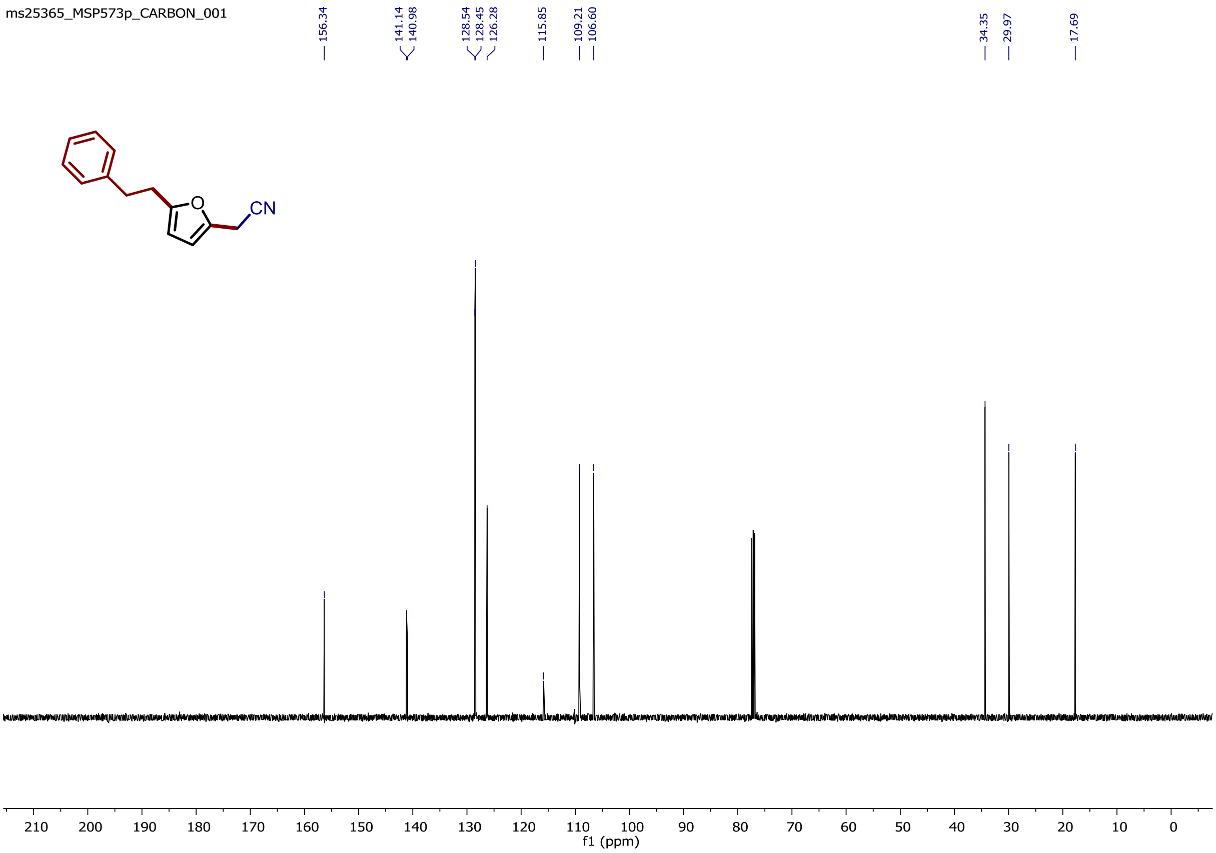

^{1}H NMR of compound **13j** (500 MHz, $CDCl_3$)

ms19274_MSP570p_PROTON_01

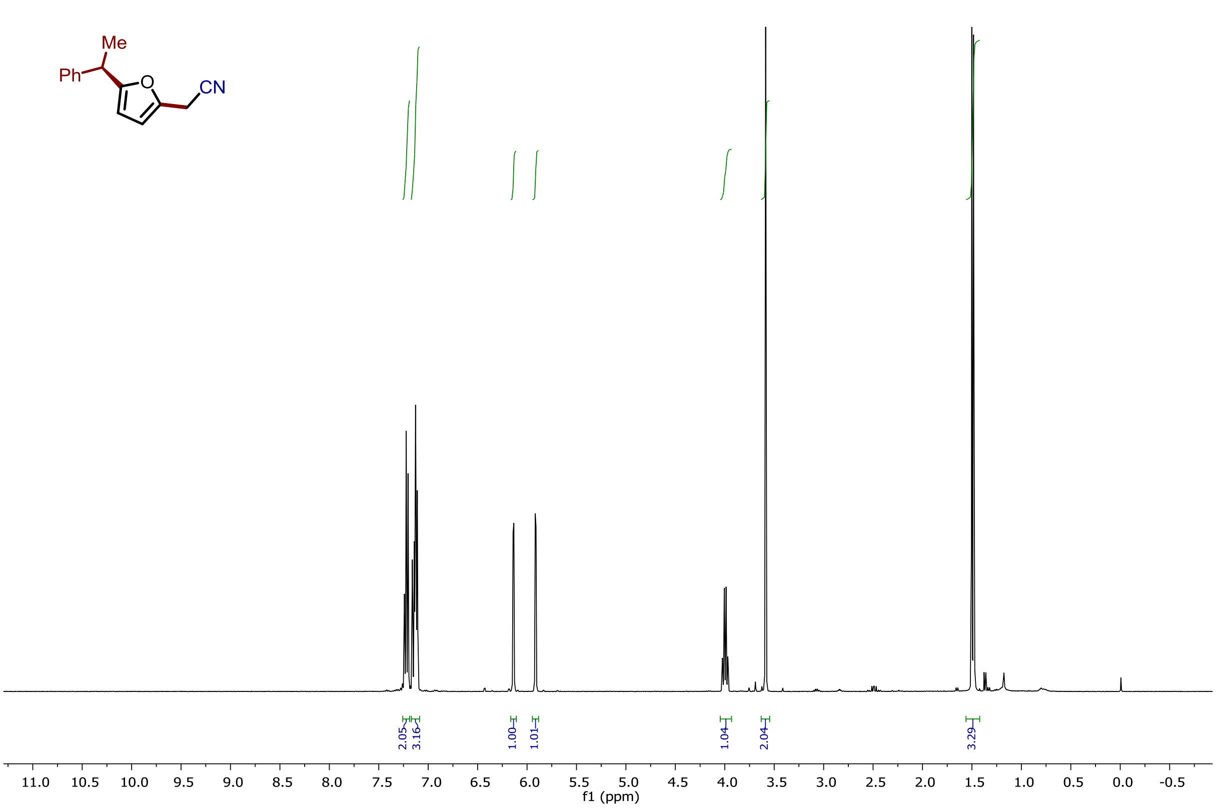

^{13}C NMR of compound **13j** (125 MHz, $CDCl_3$)

ms19274_MSP570p_CARBON_01

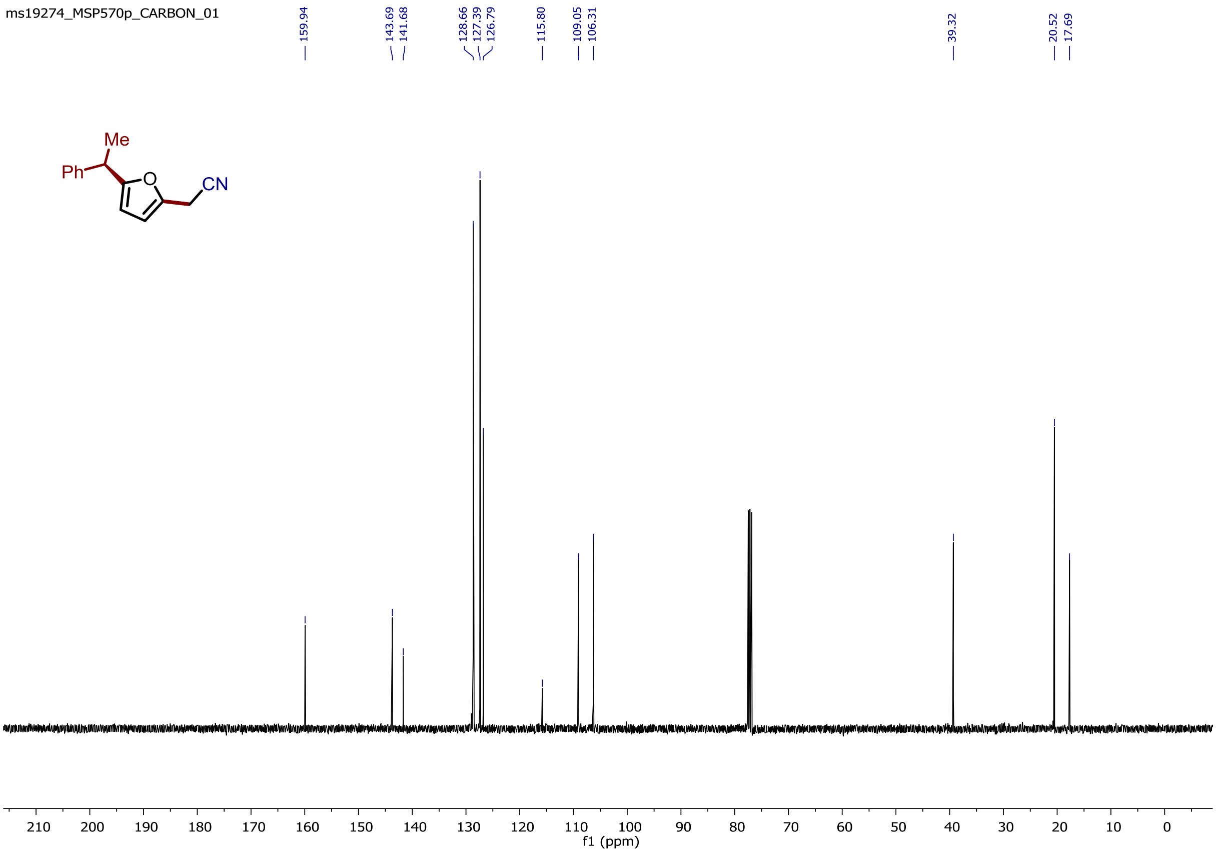

^{1}H NMR of compound **13k** (500 MHz, $CDCl_3$)

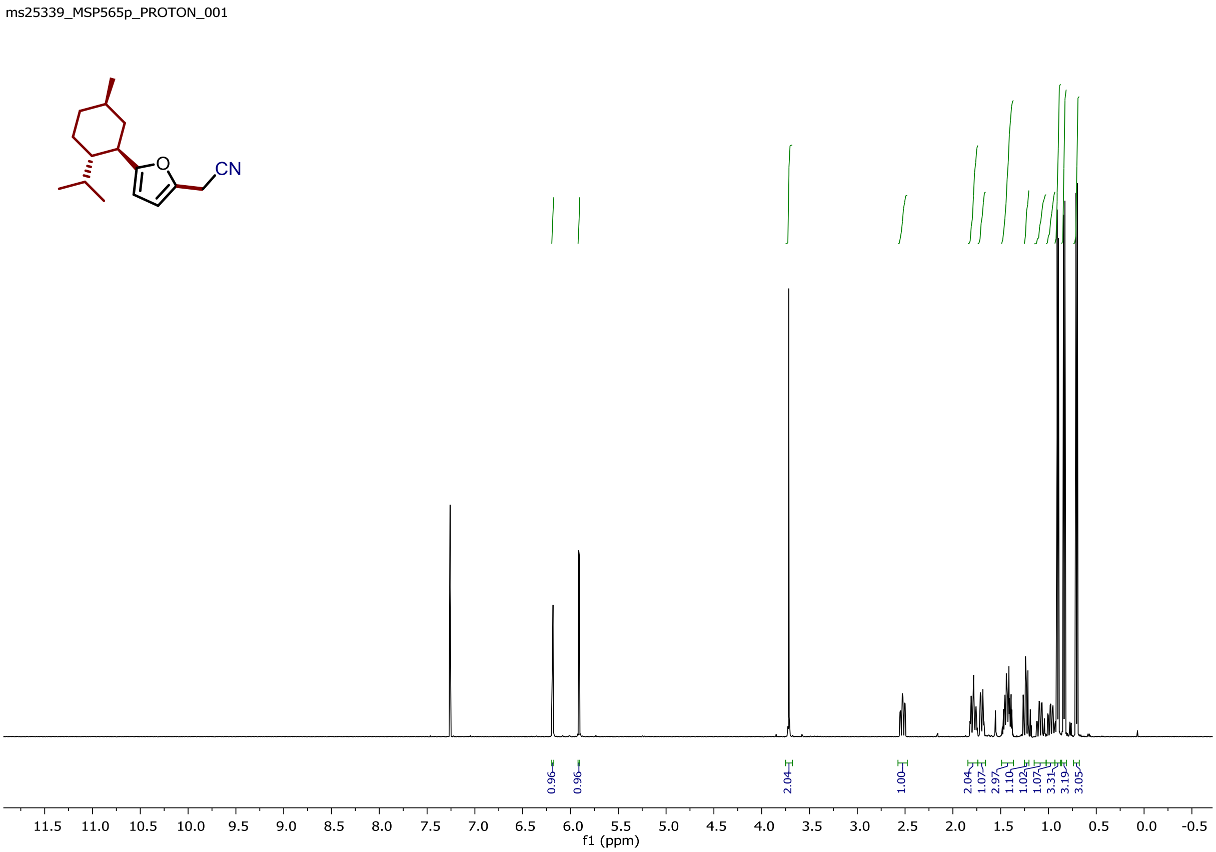

^{13}C NMR of compound **13k** (125 MHz, $CDCl_3$)

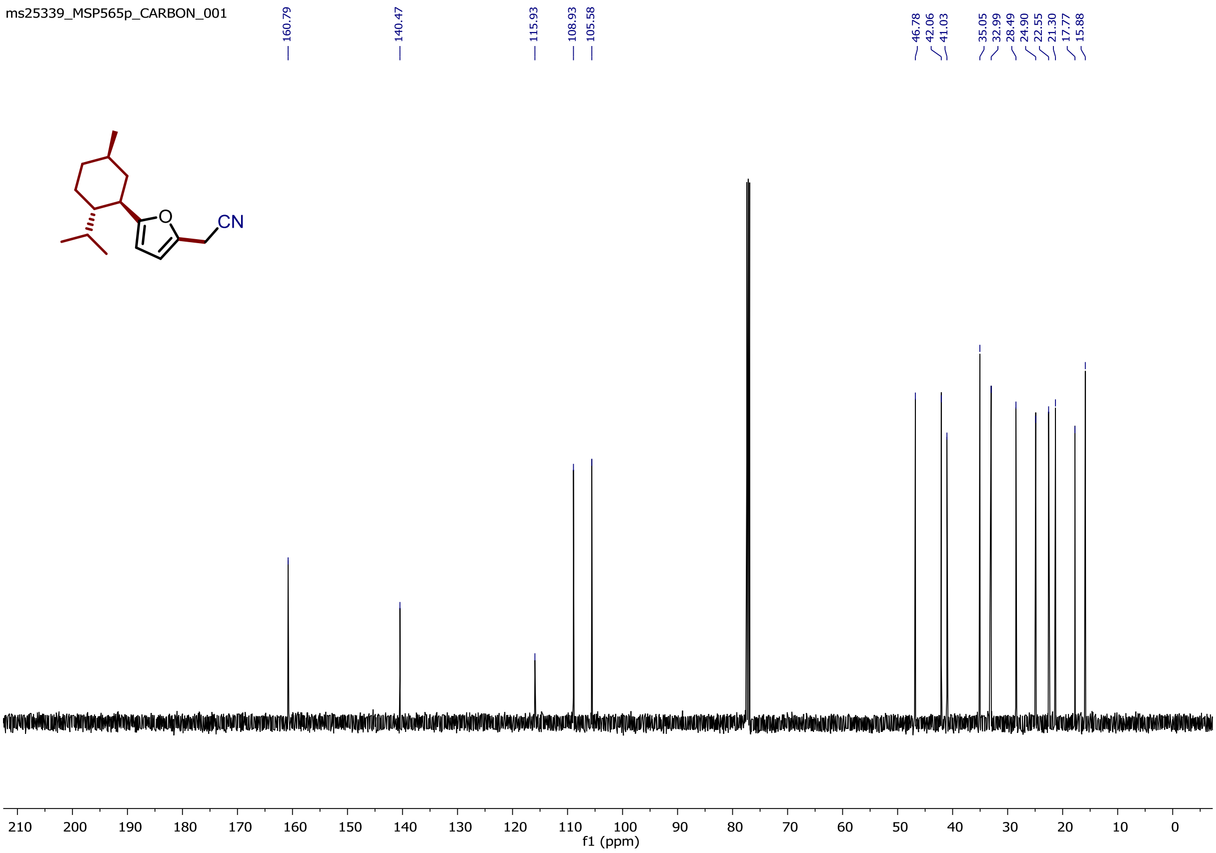

^{1}H NMR of compound **14a** (500 MHz, $CDCl_3$)

ms25512_MSP594p_PROTON_001

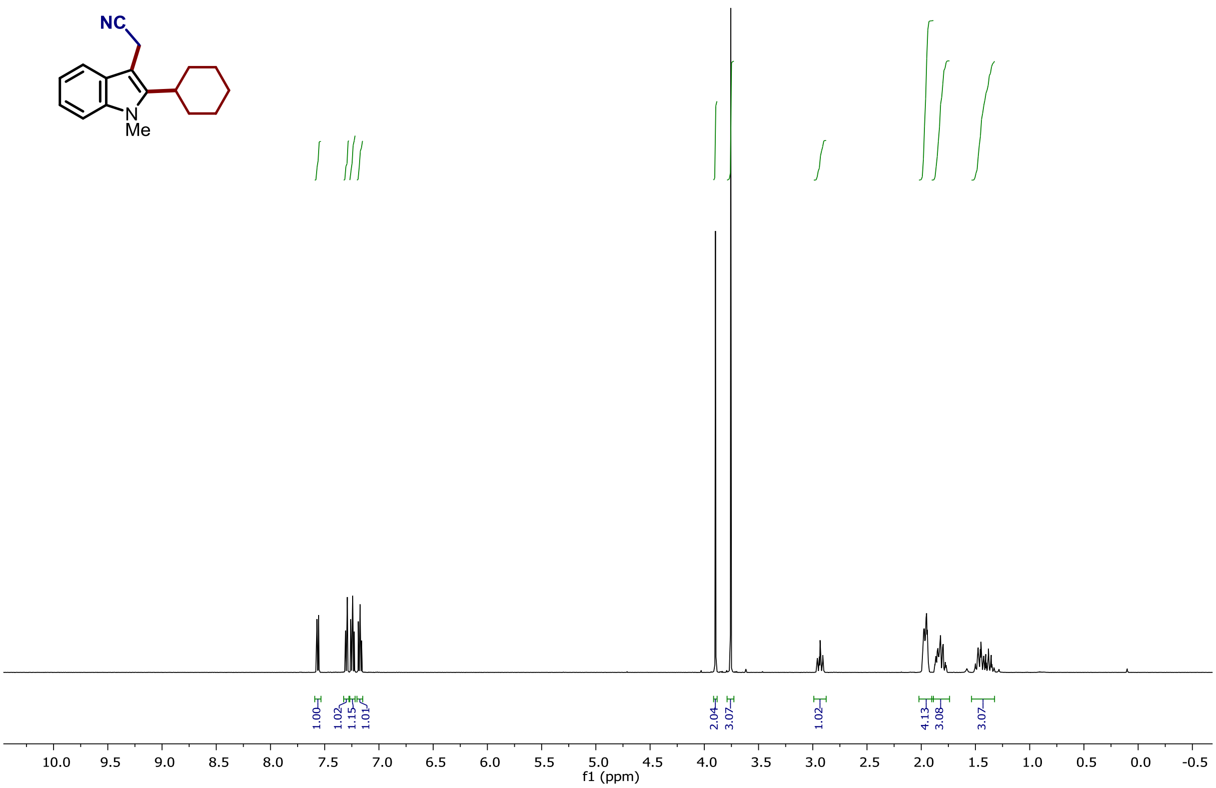

^{13}C NMR of compound **14a** (125 MHz, $CDCl_3$)

ms25512_MSP594p_CARBON_002

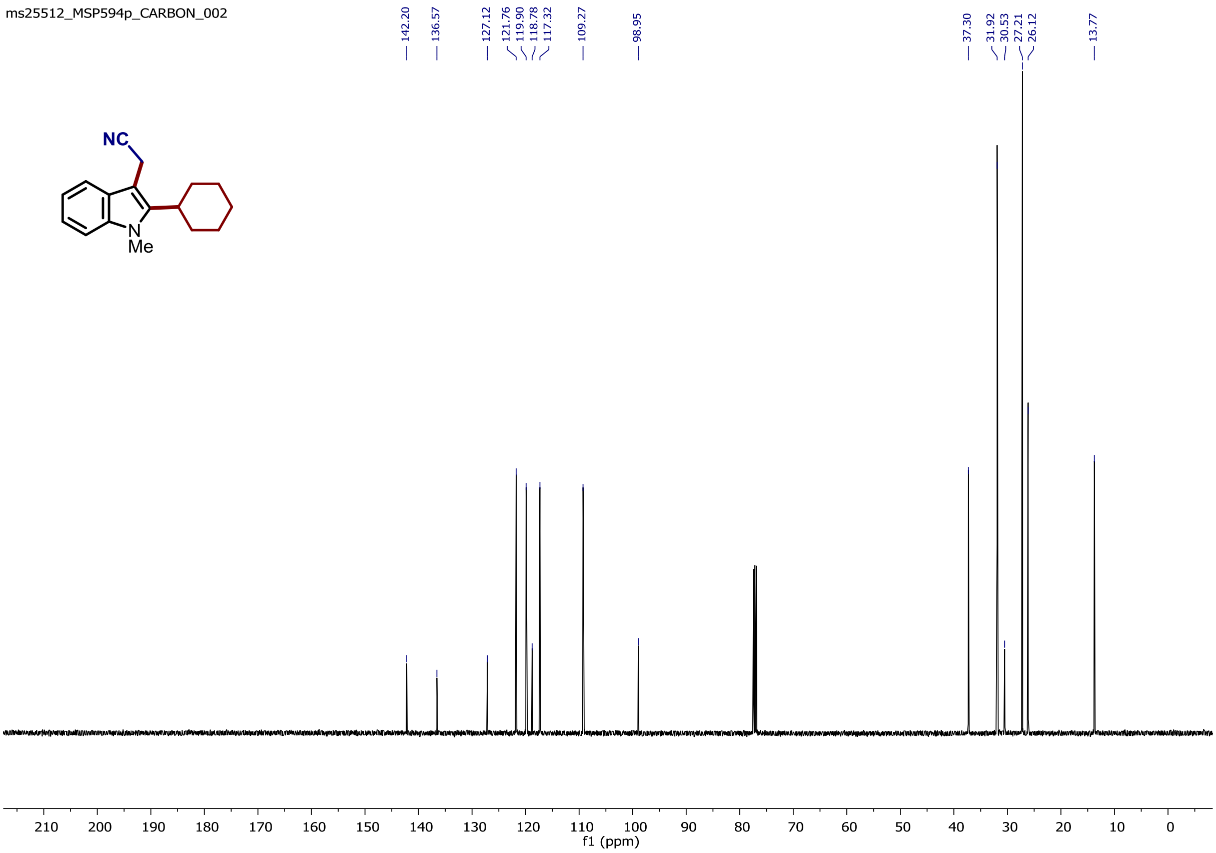

^{1}H NMR of compound **14b** (400 MHz, $CDCl_3$)

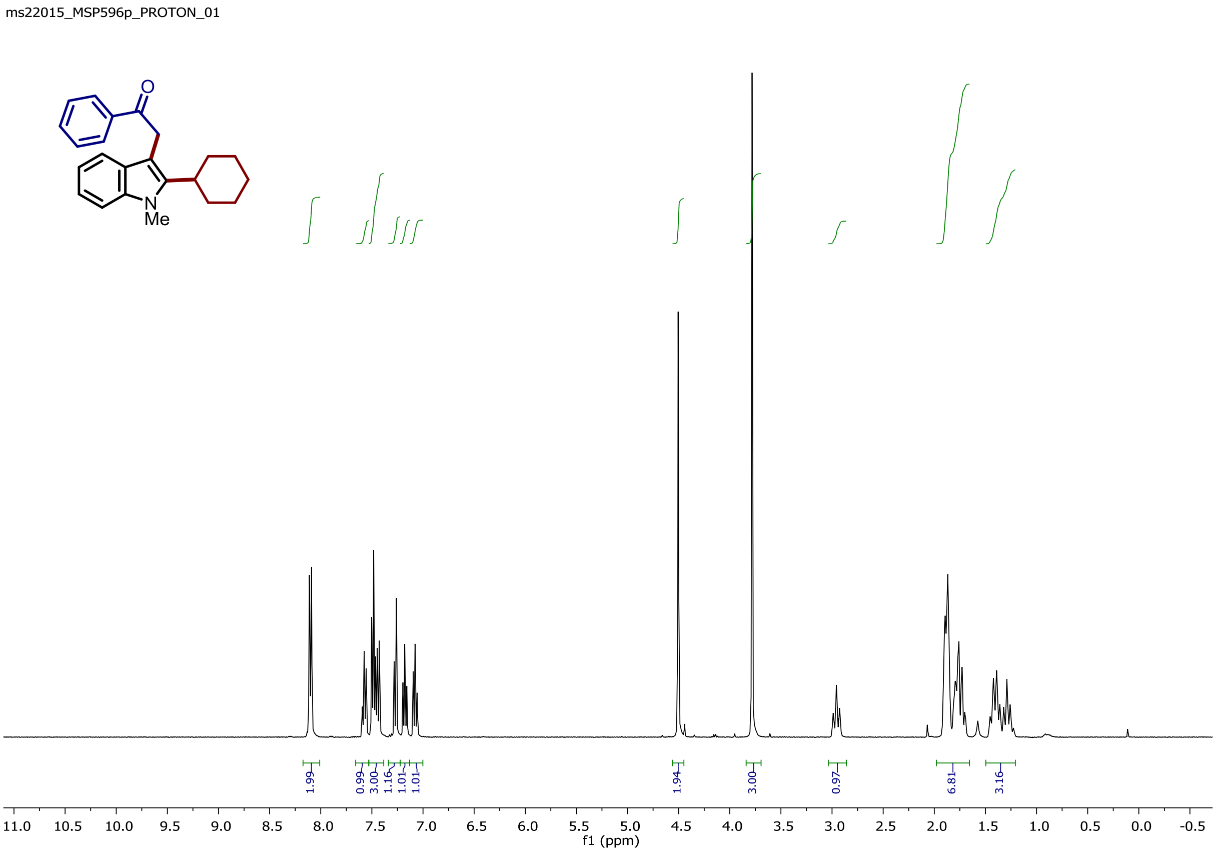

^{13}C NMR of compound **14b** (100 MHz, $CDCl_3$)

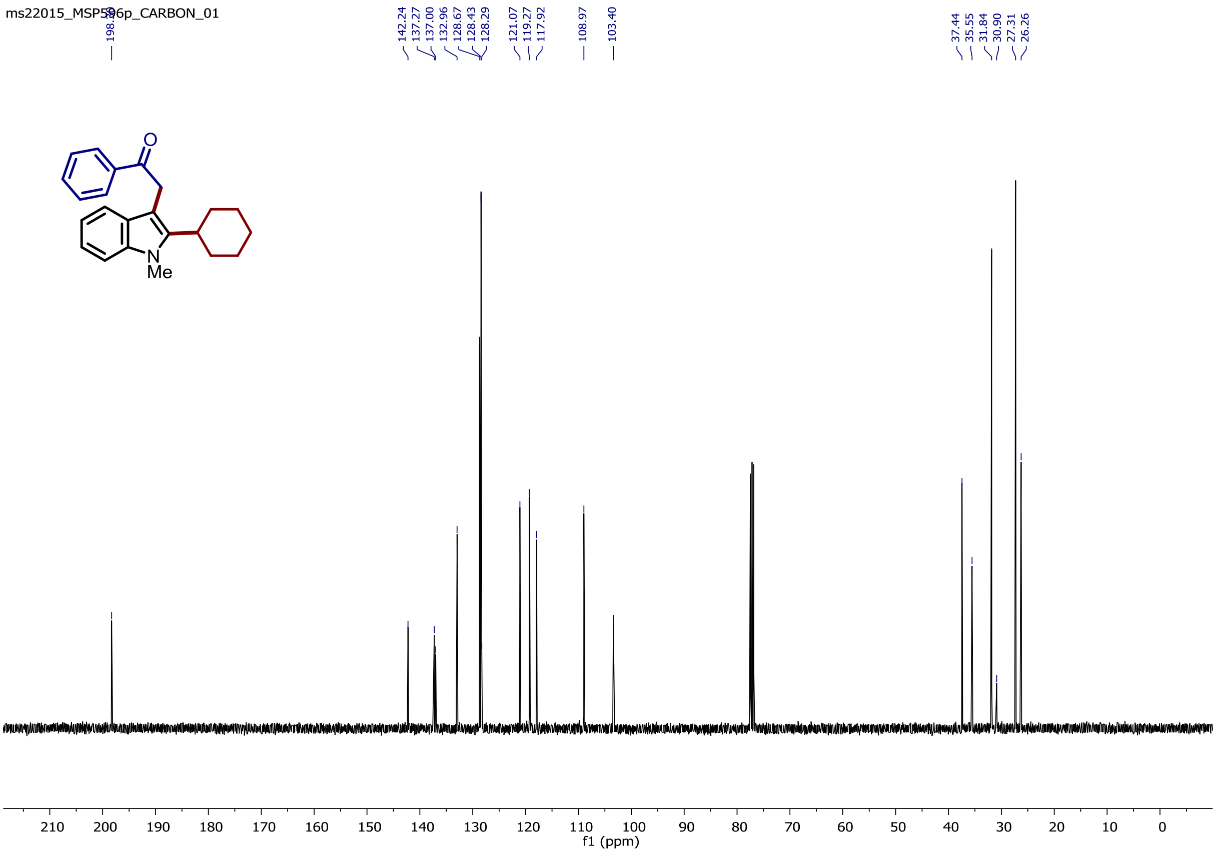

^{1}H NMR of compound **14c** (500 MHz, $CDCl_3$)

ms16271_MSP600bis_p_PROTON01

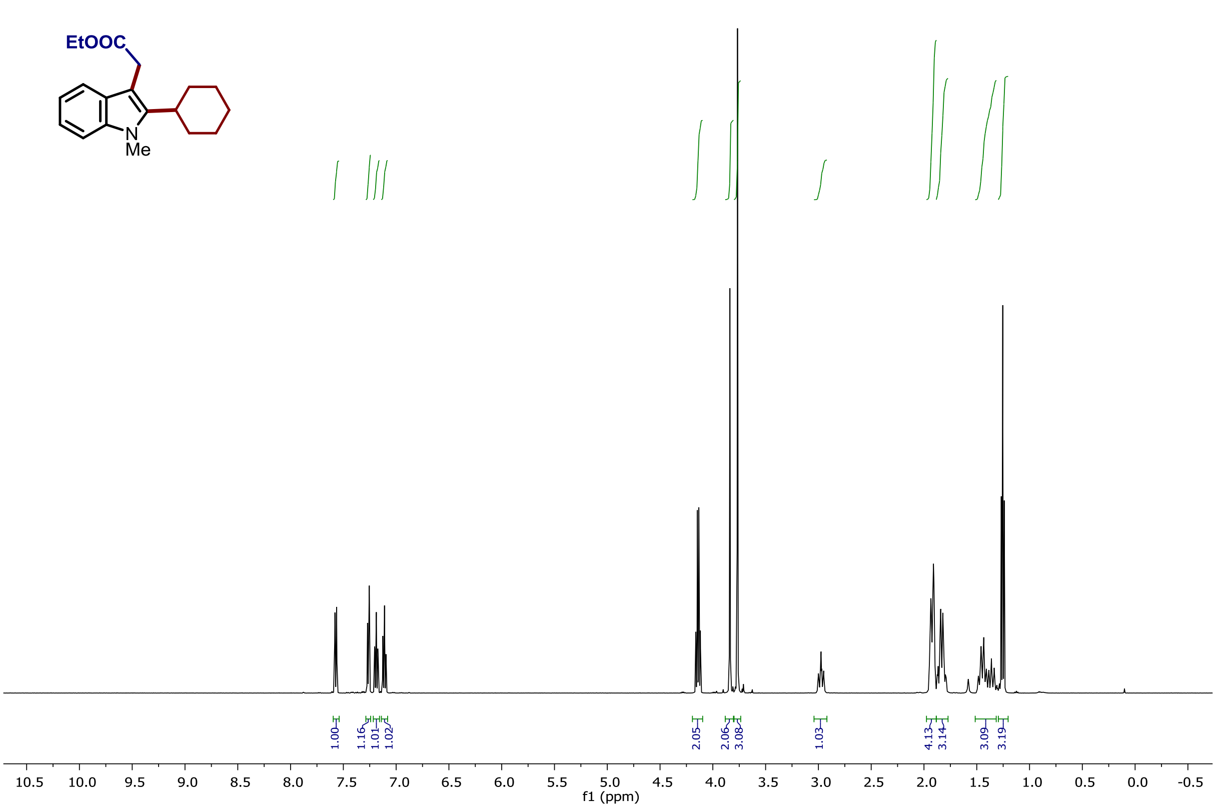

^{13}C NMR of compound **14c** (125 MHz, $CDCl_3$)

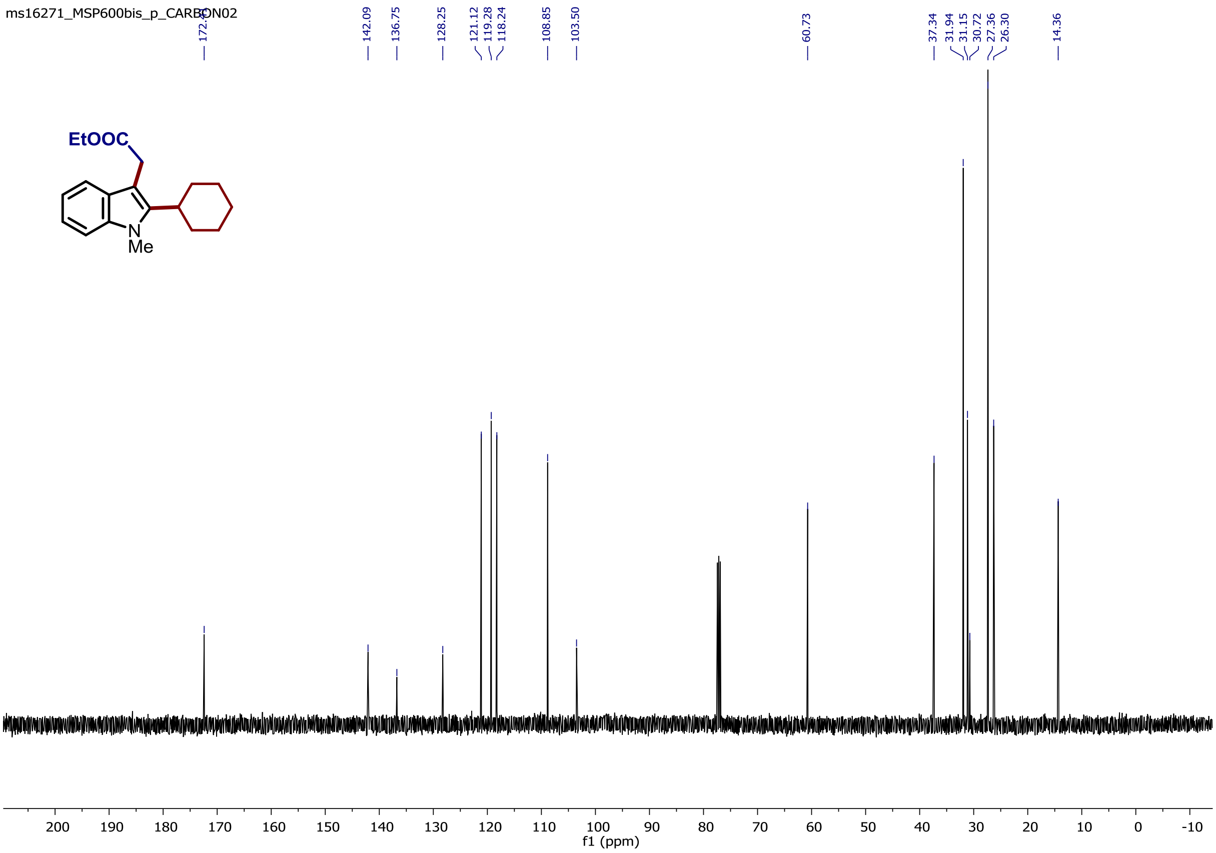

^{1}H NMR of compound **14d** (500 MHz, $CDCl_3$)

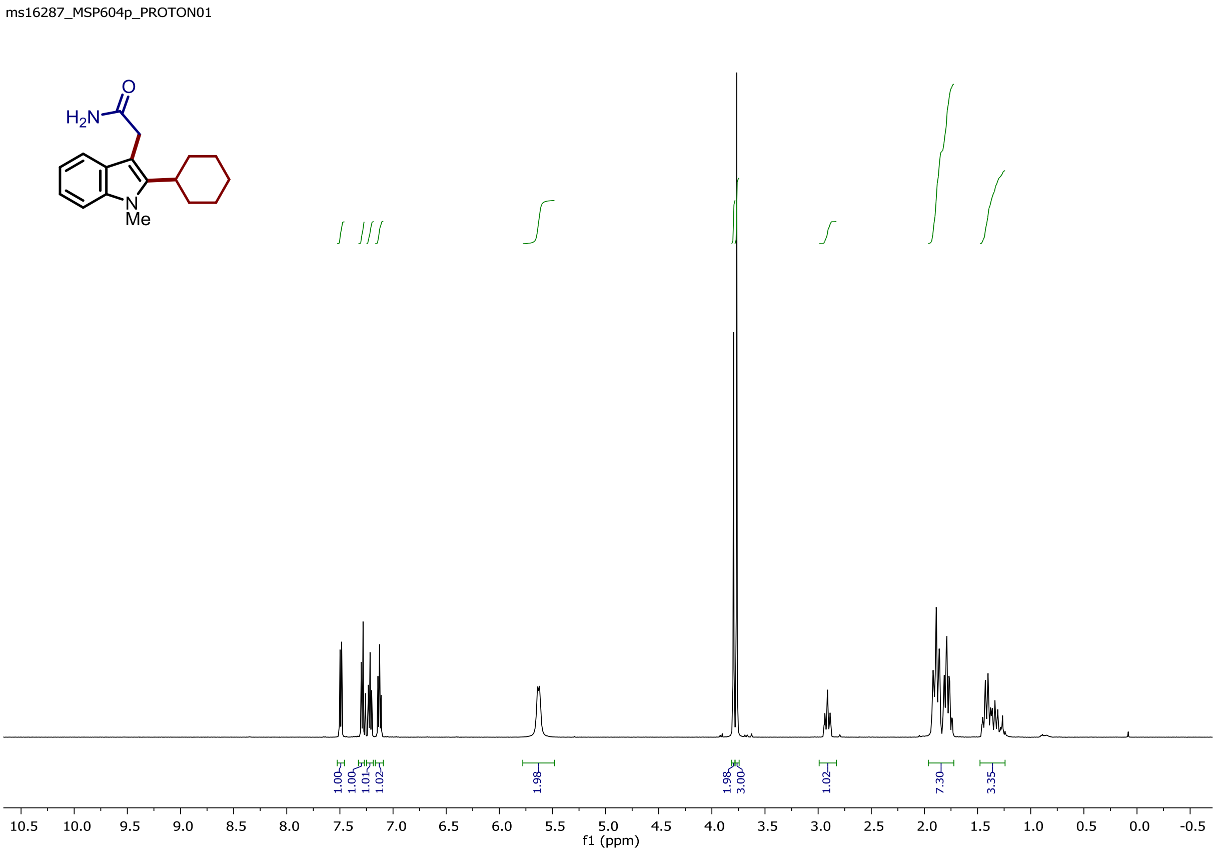

^{13}C NMR of compound **14d** (125 MHz, $CDCl_3$)

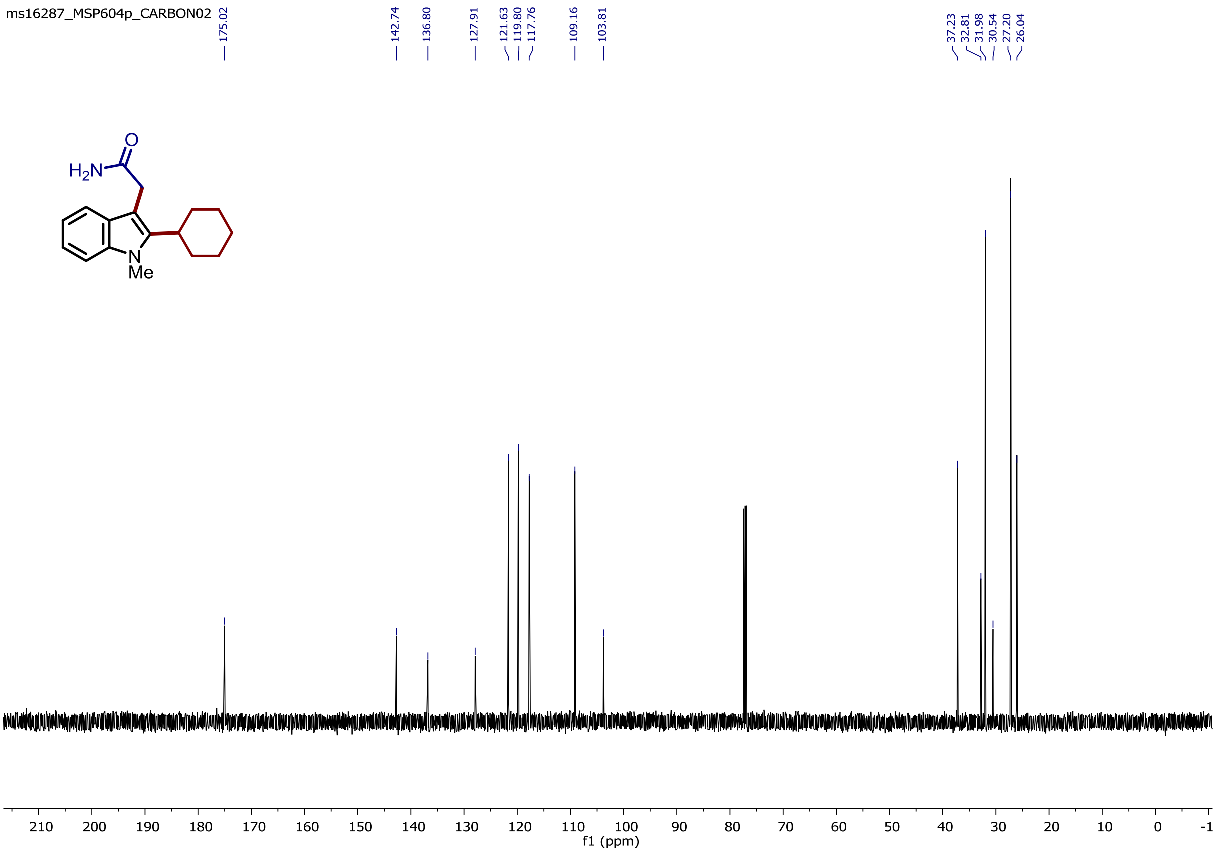

^{1}H NMR of compound **14e** (500 MHz, $CDCl_3$)

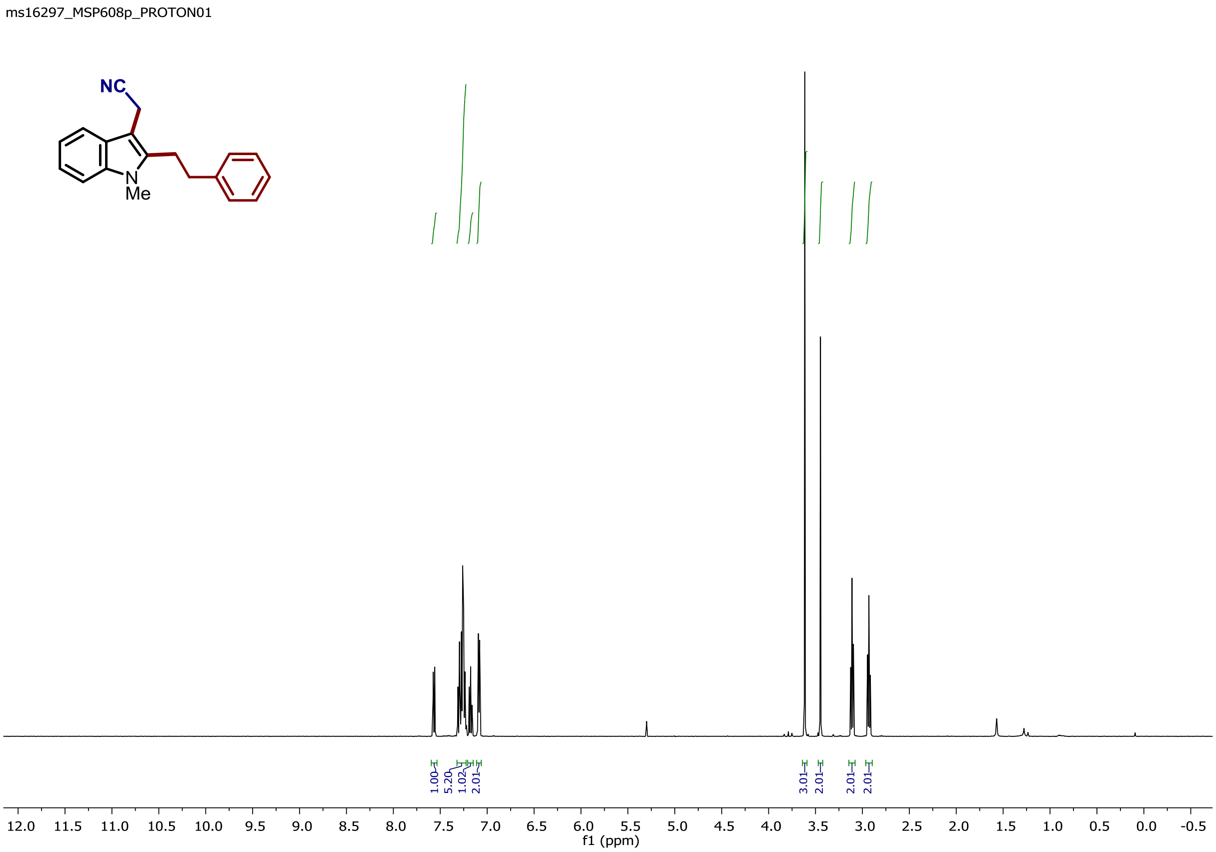

^{13}C NMR of compound **14e** (125 MHz, $CDCl_3$)

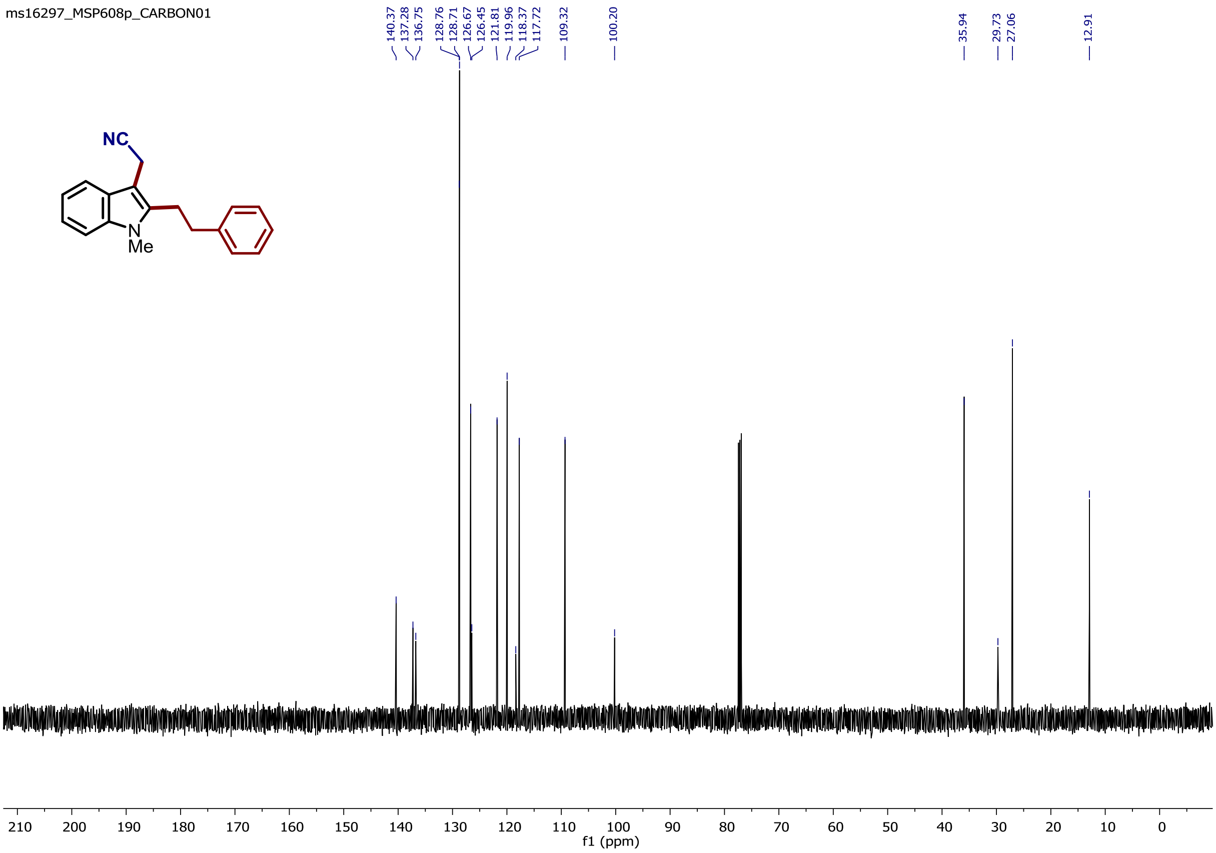

^{1}H NMR of compound **14f** (400 MHz, $CDCl_3$)

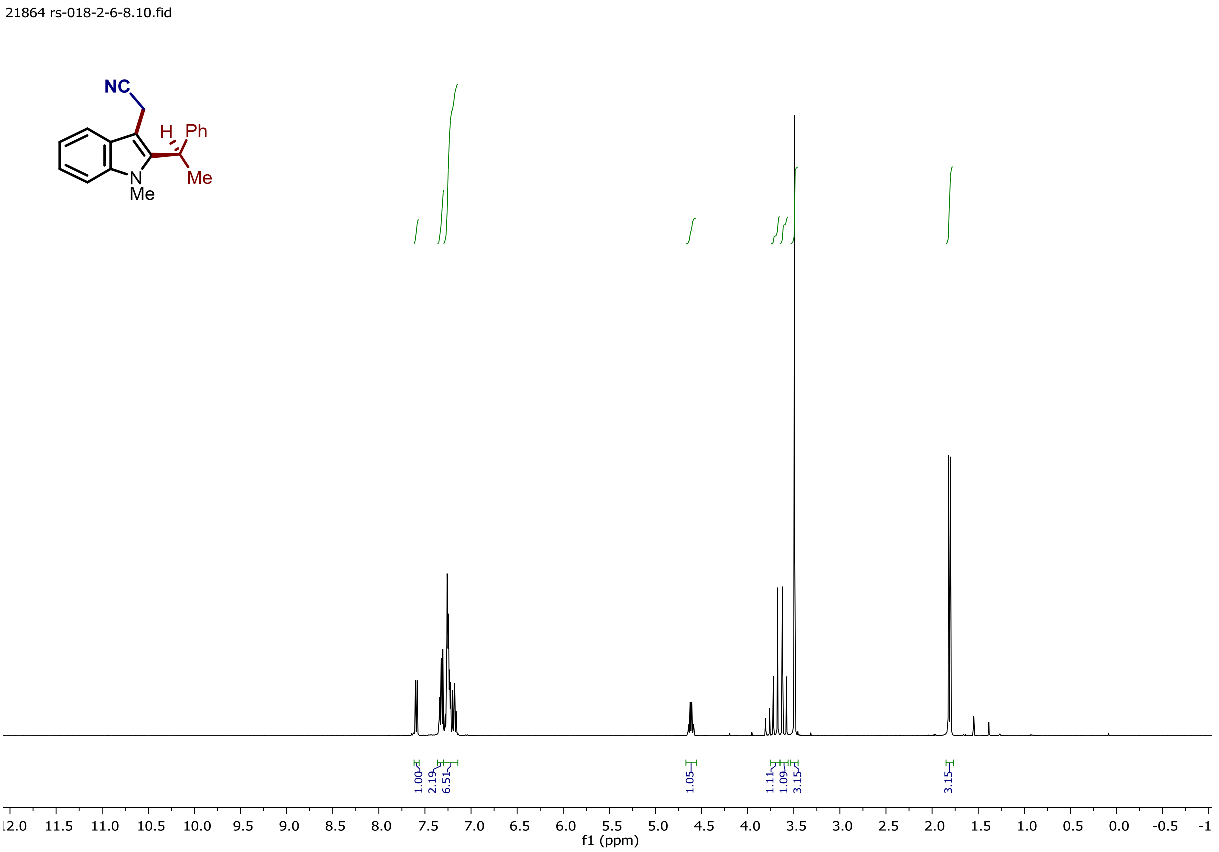

^{13}C NMR of compound **14f** (100 MHz, $CDCl_3$)

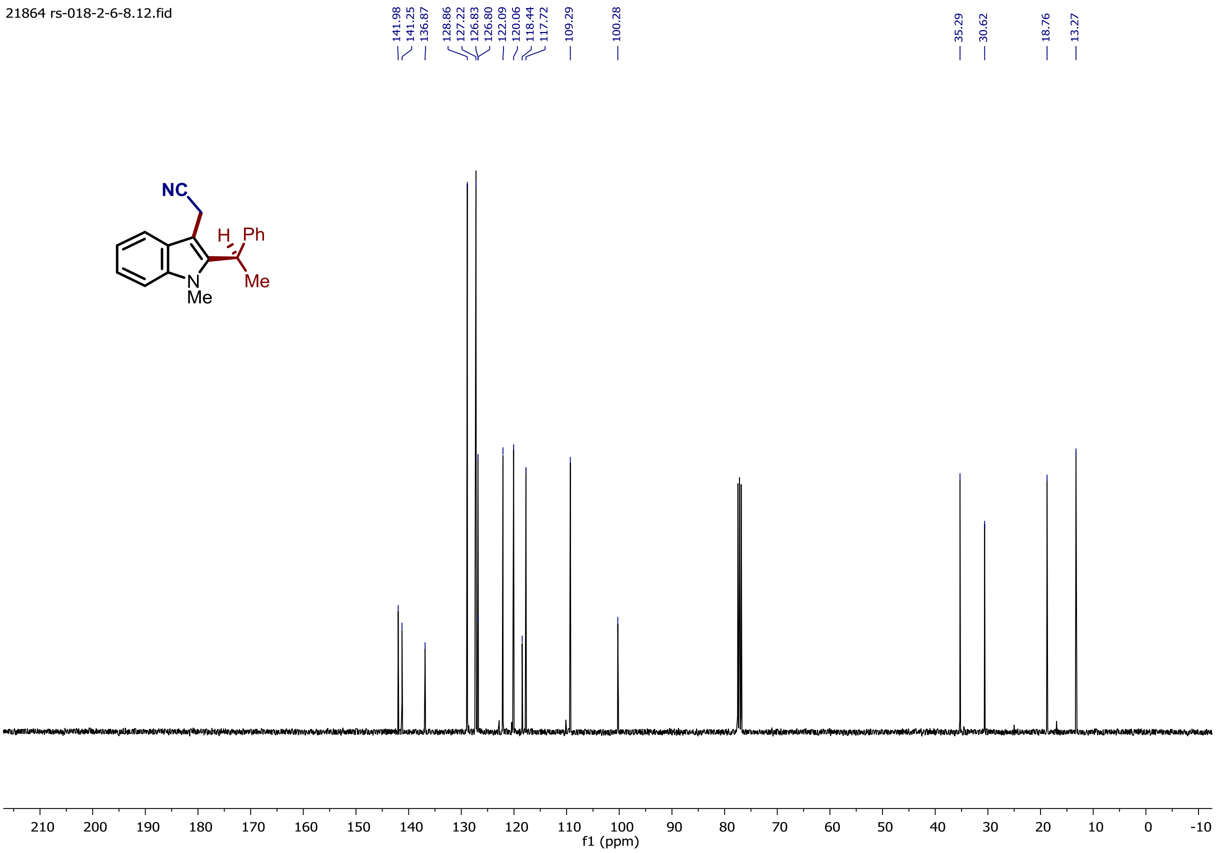

^{1}H NMR of compound **14g** (400 MHz, $CDCl_3$)

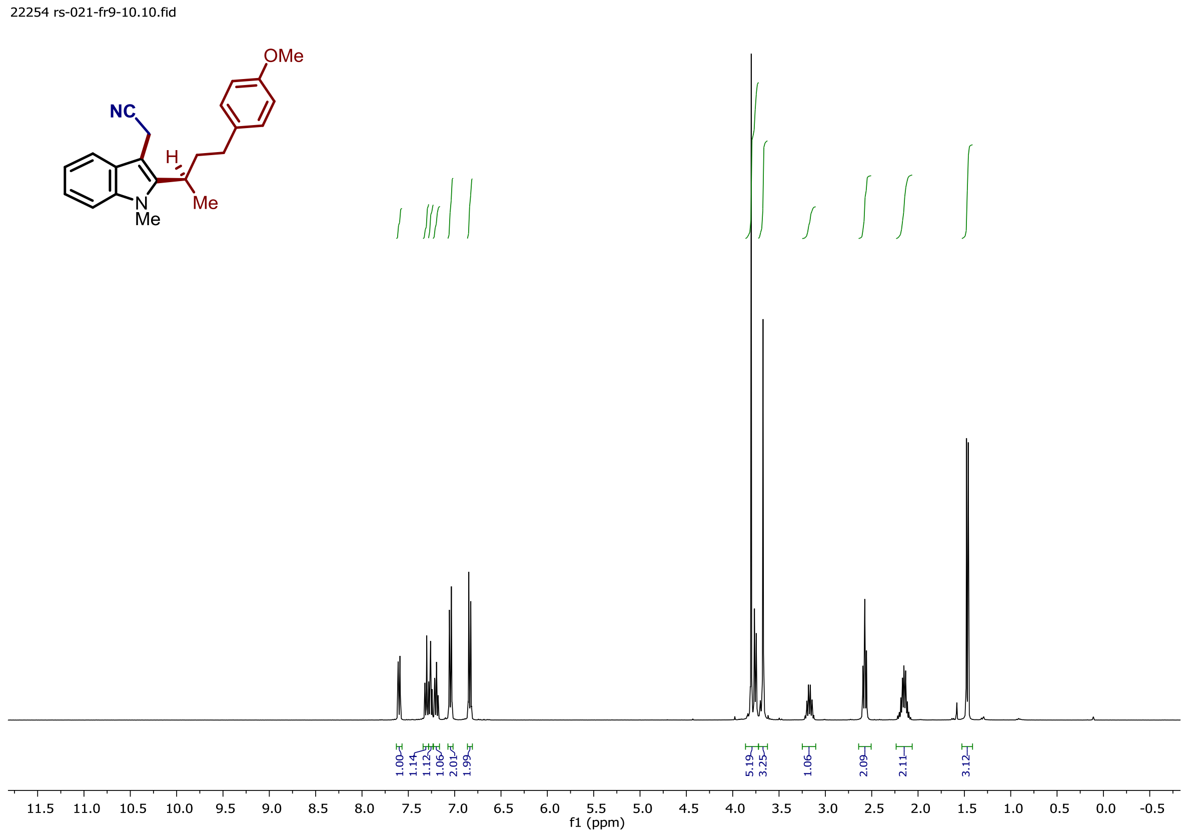

^{13}C NMR of compound **14g** (100 MHz, $CDCl_3$)

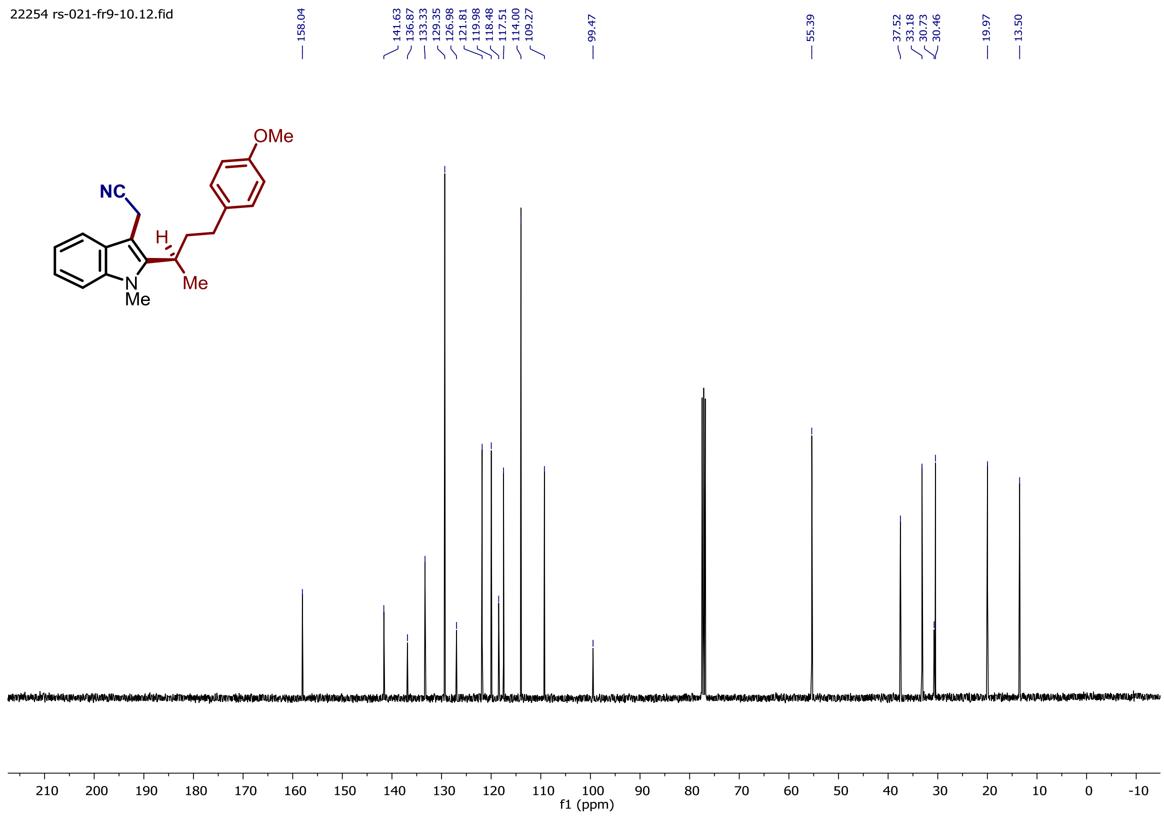

^{1}H NMR of compound **14h** (400 MHz, $CDCl_3$)

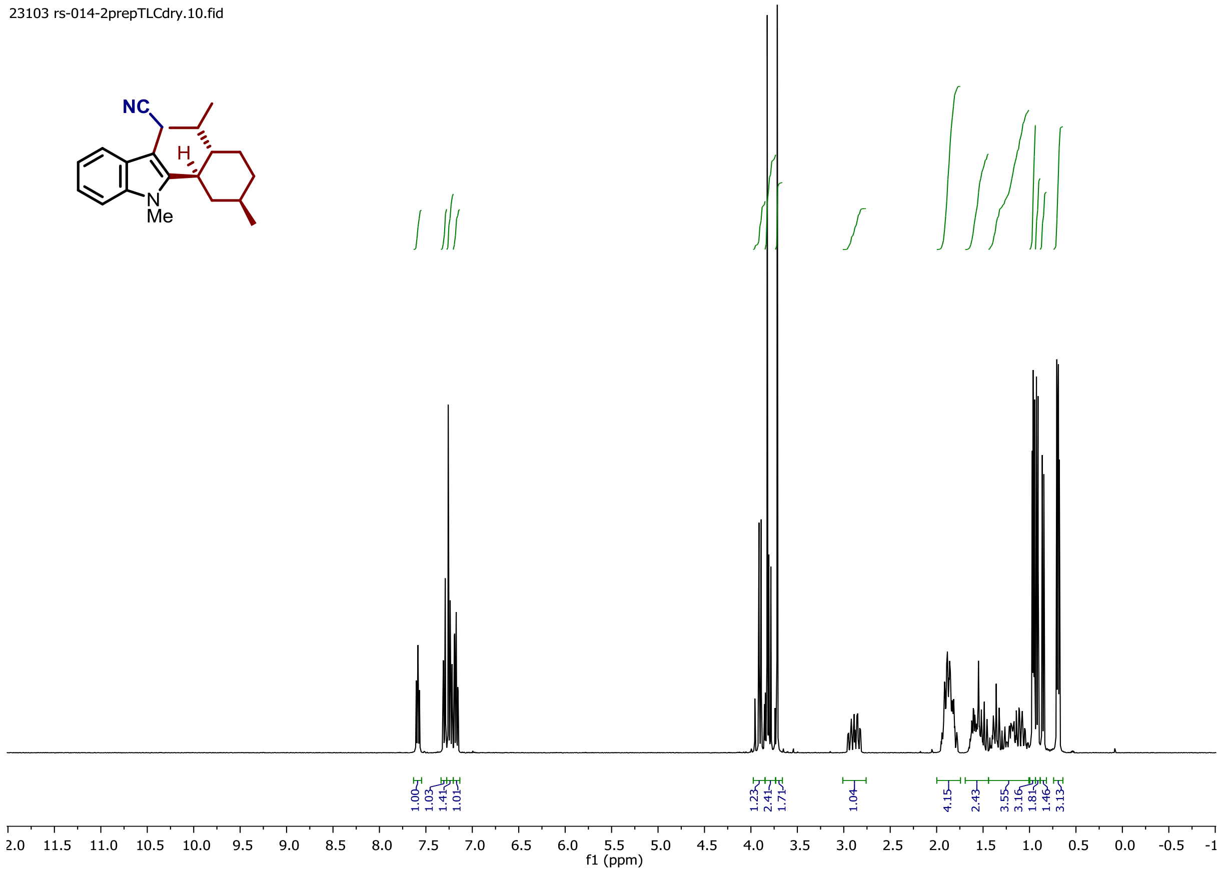

^{13}C NMR of compound **14h** (100 MHz, $CDCl_3$)

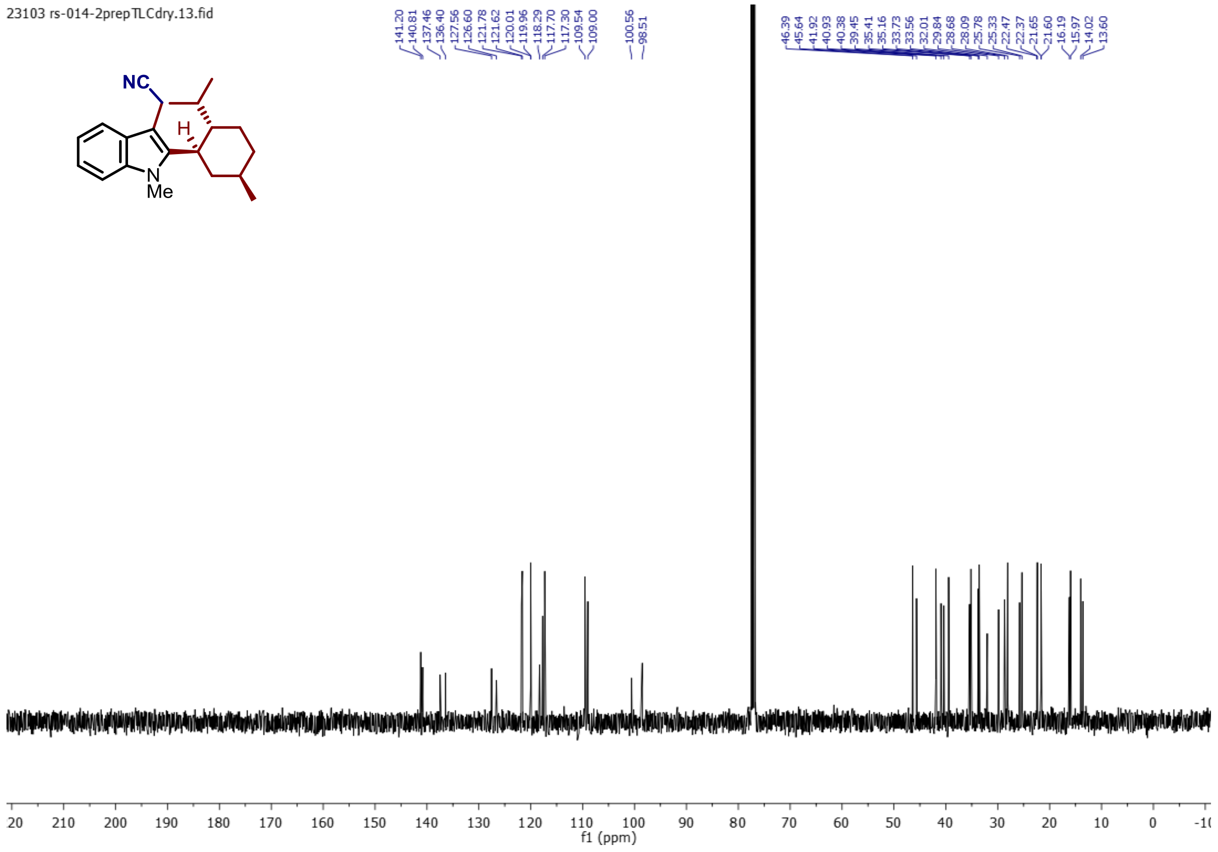

^{1}H NMR of compound **2a** (500 MHz, DMSO-d_6)

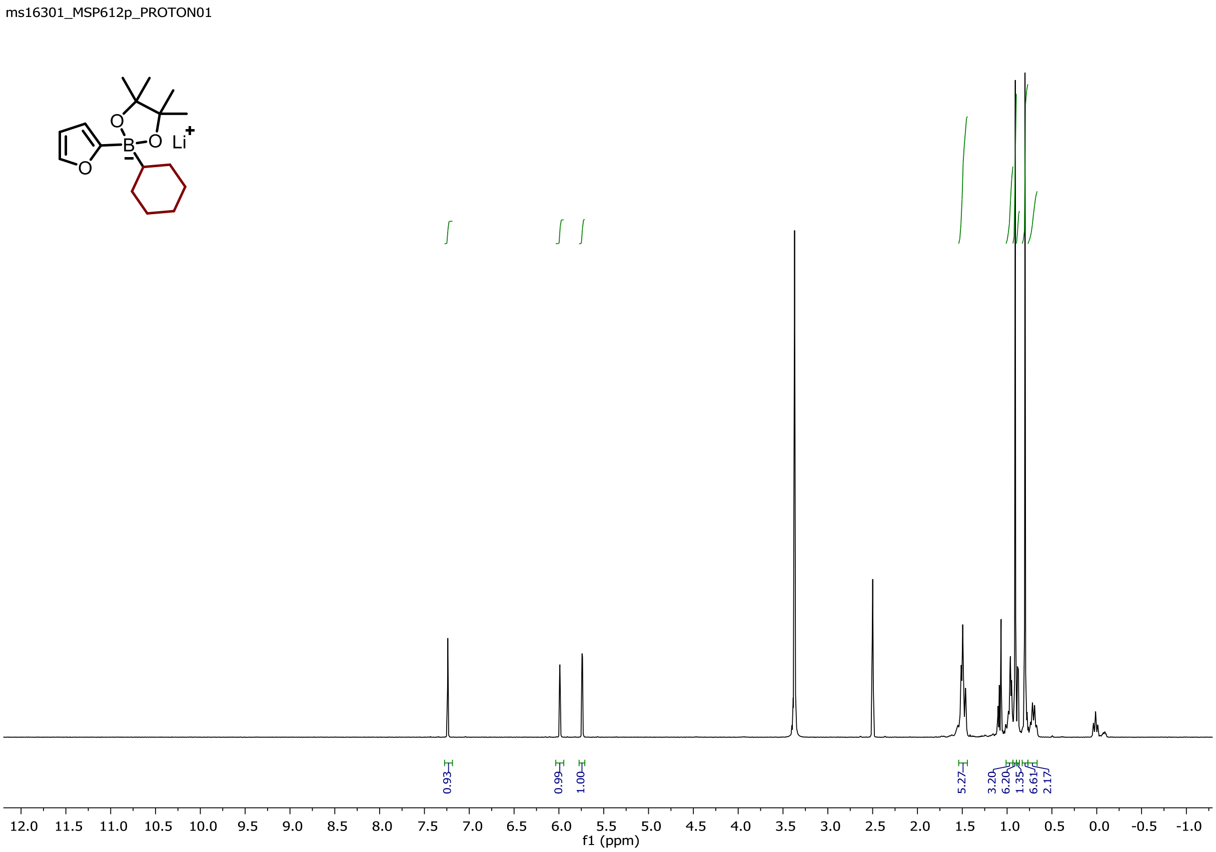

^{13}C NMR of compound **2a** (125 MHz, DMSO-d_6)

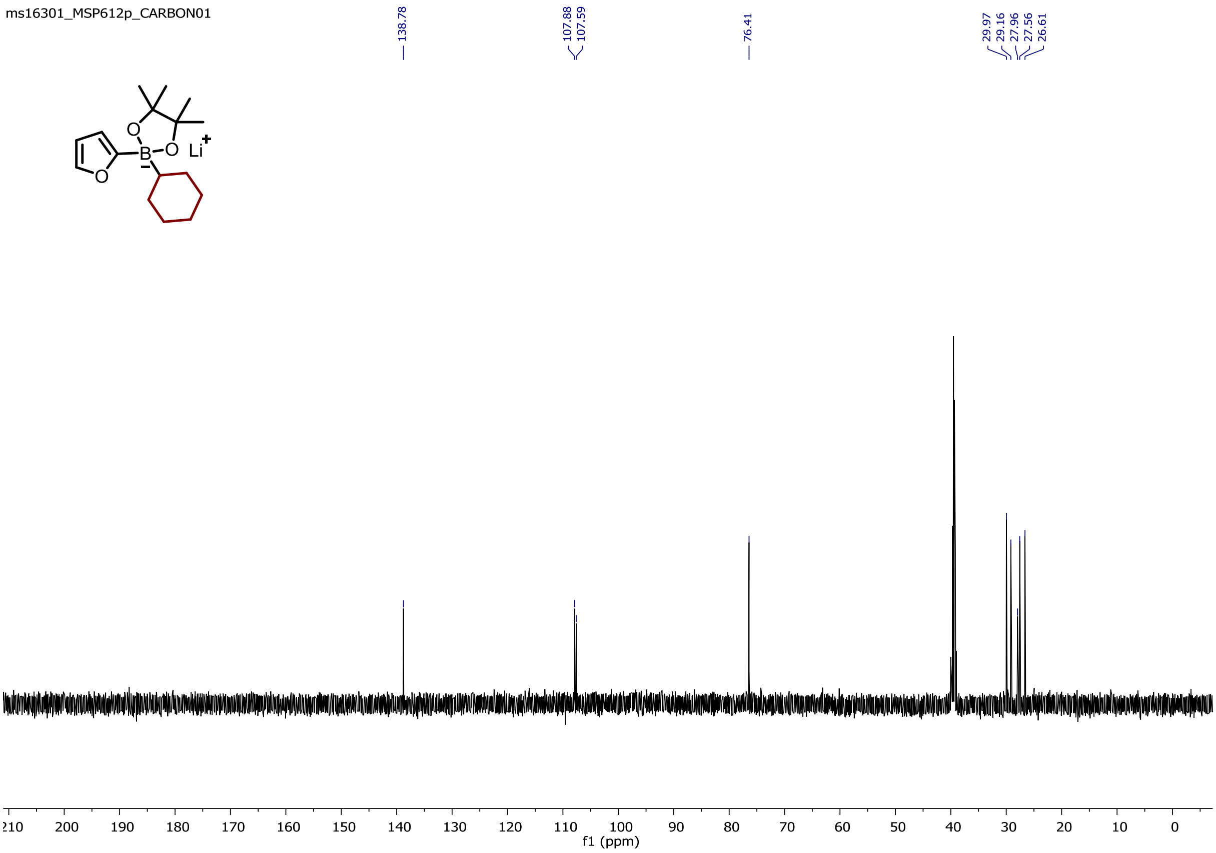

^{11}B NMR of compound **2a** (128 MHz, DMSO-d_6)

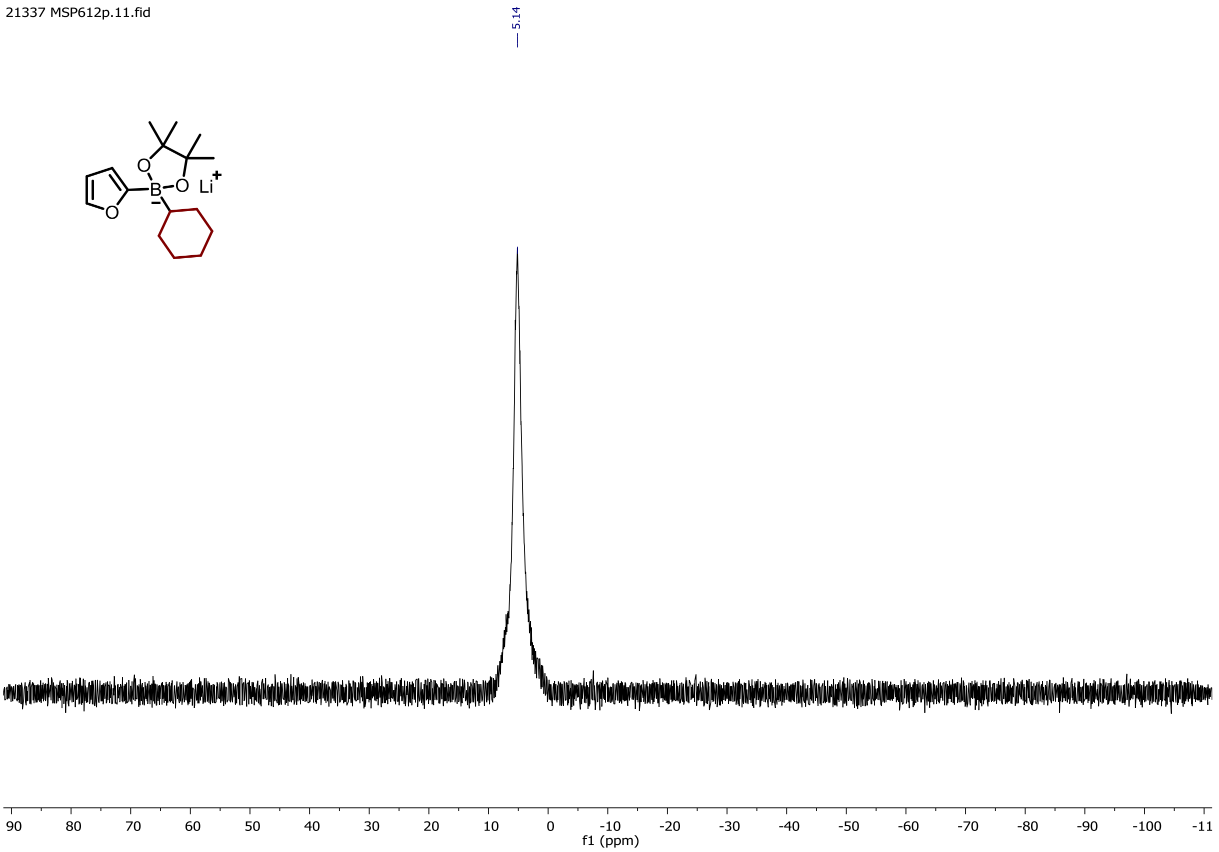

Supplement: Supplementary file 1 — Supplementary [file CHEM-24-4279-s001.pdf]
